# Online Processing of Temporal Agreement in a Grammatical Tone Language: An ERP Study

**DOI:** 10.3389/fpsyg.2021.638716

**Published:** 2021-05-21

**Authors:** Frank Tsiwah, Roelien Bastiaanse, Jacolien van Rij, Srđan Popov

**Affiliations:** ^1^Center for Language and Cognition Groningen, University of Groningen, Groningen, Netherlands; ^2^International Doctorate for Experimental Approaches to Language and Brain, University of Groningen, Newcastle upon Tyne, United Kingdom; ^3^Center for Language and Brain, National Research University Higher School of Economics, Moscow, Russia; ^4^Bernoulli Institute for Mathematics, Computer Science and Artificial Intelligence, University of Groningen, Groningen, Netherlands

**Keywords:** grammatical tone, tense, temporal agreement, event-related potentials, Akan

## Abstract

Previous electrophysiological studies that have examined temporal agreement violations in (Indo-European) languages that use grammatical affixes to mark time reference, have found a Left Anterior Negativity (LAN) and/or P600 ERP components, reflecting morpho-syntactic and syntactic processing, respectively. The current study investigates the electrophysiological processing of temporal relations in an African language (Akan) that uses grammatical tone, rather than morphological inflection, for time reference. Twenty-four native speakers of Akan listened to sentences with time reference violations. Our results demonstrate that a violation of a present context by a past verb yields a P600 time-locked to the verb. There was no such effect when a past context was violated by a present verb. In conclusion, while there are similarities in both Akan and Indo-European languages, as far as the modulation of the P600 effect is concerned, the nature of this effect seems to be different for these languages.

## Introduction

The notion of time is encoded differently across languages in the world. In many Indo-European languages such as English and Dutch, tense inflection on the verb is used to indicate whether the event happened in the past or is currently happening, whereas some Asian languages such as Chinese, Thai and Standard Indonesian use aspectual adverbs for this purpose. Interestingly, Akan, which belongs to the Niger-Congo family, uses grammatical tone to express time reference.

Most of the data on tense (dis)agreement studies come from Indo-European languages, and thus, make the study of time reference strongly biased toward certain devices (such as tense morphology) and certain languages (Klein, 2009). Therefore, it remains unclear whether findings from tense and/or time reference studies are specific to languages that use inflectional verb morphology (such as tense in Indo-European languages) or can be extended to languages that use other means of encoding time reference.

The focus of the current study is on the electrophysiological processing of time reference expressed through grammatical tone, rather than morphological inflection, using event-related potentials (ERP) brain imaging technique. The main question is whether the neural mechanism(s) related to time reference encoding in other languages are specific to those languages, or can be extended to Akan, which is a tonal language that uses tone, for temporal reference.

### Tense and Time Reference Processing

Tense can be defined as the “grammaticalized expression of location in time” (Comrie, 1985). Similarly, aspect is the grammatical specification on the verb that reflects the temporal boundary of an event by indicating whether it is completed (perfective aspect), ongoing (imperfective aspect) or repetitive (imperfective aspect) (Comrie, 1976). Many studies on aphasia have shown that the processing of tense and aspect, which indicate the time reference of an event, are problematic for agrammatic individuals (Hebrew: Friedmann and Grodzinsky, 1997; German: Wenzlaff and Clahsen, 2004, 2005; Burchert et al., 2005; Greek: Nanousi et al., 2006; Spanish: Gavarró and Martínez-Ferreiro, 2007; Dutch: Bastiaanse, 2008). Further, recent studies have demonstrated that not all time frames are equally impaired in agrammatism, and that the time frame referring to the past is usually more susceptible to being impaired than the present and usually also than the future (Bastiaanse, 2013; Dragoy and Bastiaanse, 2013; Martínez-Ferreiro and Bastiaanse, 2013; Bos and Bastiaanse, 2014; Tsiwah et al., 2020). A previous study has shown that Akan speakers with agrammatic aphasia show a dissociation between past and present habitual processing (Tsiwah et al., 2020). Moreover, reaction time studies on tense processing have shown that healthy adults show longer reaction times for time reference violations caused by a verb in past rather than present tense (Faroqi-Shah and Dickey, 2009; Jonkers et al., 2007).

Overall, the past and present time reference dissociation has been demonstrated in many languages (English and Turkish: Bastiaanse et al., 2011; Russian: Dragoy and Bastiaanse, 2013 (Dutch: Bastiaanse, 2008; Bos and Bastiaanse, 2014; Spanish and Catalan: Martínez-Ferreiro and Bastiaanse, 2013; Korean: Lee et al., 2013). Bastiaanse et al. (2011) and Bastiaanse (2013) capture the reason for this dissociation in the Past Discourse Linking Hypothesis (PADILIH), which claims that past time reference, whether expressed through tense and/or aspectual verb inflection, is selectively impaired because it requires discourse-linking. According to the PADILIH, past time reference requires a link between the time of speaking the event time, since they do not coincide, whereas for reference to the present, the time of speaking coincides with the time of the event, and hence, no discourse linking is required. Consequently, processing past time reference is more costly than present time reference.

### ERP Studies on Tense

There are only a few studies that have investigated temporal agreement violations using Event-Related-Potentials (ERPs), and most of them have focused on morphological processing of tense agreement. Using ERPs, Steinhauer and Ullman et al. (2002) presented sentences like (1a) and (1b) to native English speakers to test for tense violations. In their case, the tense violation elicited a left anterior negativity peaking around 300–500 ms after the onset of the verb, and this was followed by a centro-posterior positivity (P600), peaking around 600–900 ms. This biphasic LAN-P600 pattern observed by Steinhauer and Ullman et al. (2002) was taken as indicating morpho-syntactic processing of tense. Similar effect was observed by Newman et al. (2007), who reported LAN and P600 effects for regular past tense violations but only a P600 effect for irregular verb violations. On the basis of their results, the authors suggest that while the neurocognitive substrate involved in regular verb processing depend on compositional processing of complex forms across linguistic domains, including morphology and syntax, irregular verb are stored in lexical memory.

(1a) Yesterday, I sailed Diane’s boat to Boston.
(1b) ^∗^Yesterday, I sail Diane’s boat to Boston.

Baggio (2008) tested tense and temporal adverbs disagreement in Dutch sentences like ‘*Afgelopen zondag lakte/^∗^lakt Vincent de kozijnen van zijn landhuis*’ (‘Last Sunday Vincent painted/^∗^paints the window frames of his country house’), and replicated the biphasic LAN-P600 pattern that Steinhauer and Ullman et al. (2002) observed in English tense violations. However, Baggio (2008) took these ERP components as signatures of semantic rather than morpho-syntactic processing. Baggio (2008) suggested that the computation of temporal reference is entirely consigned to the semantic processor, and thus, the LAN effect observed for tense disagreement can be taken as reflecting a failure to simultaneously solve the temporal and/or semantic constraints set up by the adverbial and the verb.

Not only were all the above studies carried out for Indo-European languages, which are morphologically marked for tense, but also they used a word-by-word reading task and compared the ERP effect on verbs with different forms. Since the objective of the current study is to test tense processing violations, the choice of keeping the verb constant, but rather manipulating the temporal adverb, is more appropriate.

Dragoy et al. (2012), in a study in Dutch, used a more appropriate methodology and compared tense violations by measuring the ERP effect from the same verb forms, with the different temporal adverbs (in italics) causing the violations.

(2a) De kelner [die nu     de peper maalt] krijgt geen fooi.
  the waiter [who now the pepper grinds] gets no tip.
  The waiter who is now/a-moment-ago grinding the pepper doesn’t get a tip.
(2b) De kelner [die ^∗^zonet           de peper maalt] krijgt geen fooi.
  the waiter [who ^∗^a-moment-ago the pepper grinds] gets no tip.
  The waiter who is now/a-moment-ago grinding the pepper doesn’t get a tip.
(2c) De kelner [die zonet           de peper maalde] krijgt geen fooi.
  the waiter [who a-moment-ago the pepper ground] gets no tip.
  The waiter who is now/a-moment-ago grinding the pepper doesn’t get a tip.
(2d) De kelner [die ^∗^nu     de peper maalde] krijgt geen fooi.
  the waiter [who ^∗^now the pepper ground] gets no tip.
  The waiter who a-moment-ago/now has ground the pepper doesn’t get a tip.

Dragoy and colleagues (2012) found a P600 effect time-locked to the critical verb in present tense (by comparing 2a and 2b), but no ERP effect (between grammatical and ungrammatical sentences in past) was found time-locked to the critical verb in the past (by comparing 2c and 2d). That is, the ERP responses revealed a distinction between the past and the present. In Dutch, the phrase ‘the man now ground the pepper’ is not readily acceptable, although it can be grammatical in a narrative (e.g., ‘…*and now Cinderella jumped in the coach’*). For a detailed explanation of the difference between acceptability and grammaticality see Langsford et al. (2019).

The question is whether these ERP components observed in tense violation studies in Indo-European languages are restricted to tense morphology *per se*, or can be extended to tenseless languages such as Chinese or Thai, that use temporal and aspectual adverbs to make reference to time, or Akan, that expresses tense with grammatical tone. To address the former, Qiu and Zhou (2012) tested (dis)agreement between semantically enriched aspectual adverbs. These aspectual adverbs function like tense and aspect markers, but are free-standing morphemes, that are optional: they are only used when the time (course) of the event is not clear from the discourse. Qiu and Zhou (2012) used *jiangyao (indexing immediate future)* for future time reference, and *cengjing (indexing immediate past)* for past time reference. However, Chinese also has a bound grammatical morpheme for reference to the past, referred to as a grammaticalized aspectual particle (-*guo*). In their paradigm, they tested sentences in which the aspectual adverb and particle did not match the time frame, set at the beginning of the sentence by a lexical temporal adverbs and temporal noun phrases (*last month and next month*), using ERPs.

A mismatch between noun phrases and both the aspectual adverbs and the aspectual particle elicited a centro-parietal P600 effect, indicating a morphosyntactic violation. Interestingly, violations caused by aspectual adverbs also produced an N400 effect, and according to the authors this is due to the lexical nature of the aspectual adverbs. Apparently, the aspectual particle, as a bound morpheme, is more grammaticalized and is not processed at the lexical semantic level. Moreover, a sustained negativity effect was found after the target words and the final words for all types of temporal markers. This was interpreted as the brain’s attempt to repair and create a coherent representation of the sentence.

Summarizing, we can conclude that tense violations by grammatical morphemes elicits a P600 in Indo European languages (Dutch, English) and in Mandarin Chinese. When the set time frame is violated by an aspectual adverb, that is, a free-standing morpheme, a P600 is elicited, preceded by the N400 that is considered to be a lexical semantic component. This N400 component was also observed in another study of our group in Thai, a language that also uses free-standing aspectual adverbs to refer to the past (Siriboonpipattana et al., submitted.).

Only Steinhauer and Ullman et al. (2002) and Baggio (2008) report a LAN preceding the P600. This may be because, as noted above, the words on which they measured in the correct and violated conditions were not the same. For example, Steinhauer and Ullman et al. (2002) compared *Yesterday she sails*… and *Yesterday she sailed*. Such a paradigm is common, but may not be optimal. Dragoy et al. (2012) and Qiu and Zhou (2012) compared similar words in a similar context (…*the man who just/now the pepper ground*…) and did not find a LAN. Hence, the P600 is the common component in studies to time reference violations through grammatical morphology. Interestingly, in languages with aspectual adverbs like Chinese (Qiu and Zhou, 2012) and Thai (Siriboonpipattana et al., submitted.) an N400 is reported as well, suggesting that in these constructions, there is a lexical semantic component.

The interpretation of the P600 is slightly different between authors: According to Steinhauer and Ullman et al. (2002), the (biphasic LAN +) P600 represents a morphosyntactic repair process, whereas Baggio (2008) interpreted the biphasic response (LAN + P600) as a reflection of semantic processing. Dragoy et al. (2012) are somewhere in between, by relating the P600 to discourse linking, which is at the interface of syntax and semantics. Qiu and Zhou (2012) associated the P600 effect found for time reference violations and the bound particle with lexical semantic and morphosyntactic processes.

Therefore, the processing of tense using ERPs relies on two components, that is, the N400 and P600. These components used to be described as reflecting semantic and syntactic processing, respectively (e.g., Kutas and Hillyard, 1980; Hagoort et al., 1993). However, such a clear-cut dichotomy has been challenged, and alternative accounts have been proposed (e.g., Kolk et al., 2003; Brouwer et al., 2012). Instead of the well-established description of the P600 effect as a marker of syntactic repair and reanalysis (re-parsing), the Monitoring Theory (e.g., Kolk et al., 2003; Vissers et al., 2008) suggests that the role of the P600 is that of a sentence monitoring and a more general reanalysis process. Once the parser encounters an unexpected stimulus, the anticipated representation is in conflict with the input representation. If such conflict is strong, the reanalysis process takes place and results in the P600 effect (Van De Meerendonk et al., 2010). A mild conflict (e.g., a semantically mildly implausible noun in a sentence) would result in integration difficulty only, but would be easily resolved, resulting in the N400. A somewhat different view on the functional characteristics of the N400 and P600 is given by the Retrieval-Integration account (Brouwer et al., 2012, 2017). According to this model, the N400 reflects the retrieval of lexical information, whereas the P600 integrates the retrieved information into the utterance.

Nonetheless, among time referencing studies, the presence of the N400 is mainly understood as indicting tapping into the semantic nature of time reference, mostly in instances in which the tense is expressed via more lexical means. Generally, the P600 has been found to index syntactic anomalies that require reanalysis and/or repair (Osterhout and Mobley, 1995; Friederici et al., 2002; Kaan and Swaab, 2003). The P600 component has a long latency, with its effect starting from around 500 ms after the onset of a target word, and sometimes extending beyond 1000 ms (see Kaan and Swaab, 2003). Depending on the nature of the syntactic anomalies (pure syntactic violations vs. syntactic ambiguity), the P600 can have either a centro-parietal or a fronto-central scalp distribution (for review see Hagoort et al., 1999; Friederici et al., 2002). The P600, however, reflects the processing of tense violation, that is, temporal disagreement between the contextually given time reference (usually by an adverb) and inflectional tense morphology. Dillon et al. (2012) have shown that Hindi speakers react differently to the same morphological (verb) violation when the violation has different underlying causes. They reported that when semantic cues predicted verbal morphology, verbal forms causing violations elicited early posterior negativity followed by a weaker P600. However, when morphosyntactic cues predicted verbal morphology, the same verbal forms causing violations elicited a right-lateralised anterior negativity (RAN) followed by a stronger P600. According to Dillon and colleagues, how the parser is able to recover from incorrect structural analyses is largely influenced by the availability of information about the cause and the content of the error.

The contribution the current study makes is that it examines tense processing in an understudied African language in which time reference is expressed neither lexically (i.e., temporal adverbs) nor through inflectional morphology, but rather through grammatical tone.

### Features of Akan

The Akan is a tonal language, and has two basic tones: High tone (H) and Low tone (L), which are pronounced with pitch level (Dolphyne, 1988; Abakah, 2000, 2005). That is, the meaning of a sentence in Akan depends not only on the vowels and the consonants that make up the words, but also on the pitch with which each syllable of the sentence is produced (Dolphyne, 1988; Osam, 2003, 2008). Similar to Chinese, Akan tones have lexical functions (e.g., pàpá – ‘father’, pápá – ‘good’, pàpà – ‘fan’), but unlike Chinese, tones in Akan also serve grammatical functions (see examples 3a, 3b and 3c), and thus, certain grammatical categories such as verb forms (tense/aspect) can be distinguished by tone (Dolphyne, 1988). Below are examples of grammatical tone in Akan.

3a) Papa no   twèr$\overset{´}{\varepsilon }$        l$\overset{´}{\varepsilon }$t$\overset{`}{\varepsilon }$
  Man the   write-HAB   letter.
  The man writes letter(s)
3b) Papa no   twèr$\overset{`}{\varepsilon }$$\overset{`}{\varepsilon }$        l$\overset{´}{\varepsilon }$t$\overset{`}{\varepsilon }$
  Man the   write:PAST    letter
  The man wrote letter(s).
3c) Papa no   b$\overset{´}{\varepsilon }$twèr$\overset{`}{\varepsilon }$$\overset{`}{\varepsilon }$      l$\overset{´}{\varepsilon }$t$\overset{`}{\varepsilon }$
  Man the   write:FUT     letter
  The man will write letter(s).

The difference between the habitual and the past is indicated predominantly by tone. The present habitual is marked by a high tone on the final syllable of a verb, hence, tonal marking on the verb for the habitual aspect on a disyllabic verb is Low – High (3a). However, the past has a Low-Low tone, with a prolonged tone on the last syllable (as in 3b). Another area in which the Akan habitual and past differ has to do with locating specific event in time. Although the Akan habitual aspect does not locate a specific event in time, and thus, cannot be referred to as the present tense, it is used to express present time (Boadi, 2008). Boadi (2008) suggests that since Akan habitual aspect semantically connotes the idea of present time rather than the past, it is appropriate to refer to it as ‘Present habitual’. On the other hand, the Akan past asserts that the event described by the verb took place at a time earlier than the time of speaking (Boadi, 2008). That is, while the Akan present habitual does not refer to a specific event time, the Akan past does. Akan also has a Past habitual, which connotes an iterative event that occurred in the past. Akan past habitual is indicated by a clause-initial particle ‘na’, followed by the same form as the present habitual. Since the past habitual requires the use of the past particle, the verb remains habitual, as illustrated in (4).

(4) Na papa no    twèr$\overset{´}{\varepsilon }$       l$\overset{´}{\varepsilon }$t$\overset{`}{\varepsilon }$s da biaa
  PAST man the   write-HAB   letter day every
  The man wrote letters every day.

### ERPs in Tonal Languages

The majority of ERP studies in tonal languages have measured the neurophysiological correlates of lexical tone processing at the pre-attentive stage (Fritz et al., 2007), and focused on the inattentive ERP components, such as the Mismatch Negativity (MMN). The MMN is a scalp-recorded event-related brain potential that reflects detection of early cortical stages of auditory processing regardless of whether the participant is paying attention (Näätänen, 2001; Näätänen et al., 2005; Pulvermüller and Shtyrov, 2006; Yue et al., 2014). While the MMN is an index of pre-attentive processing, the P300 reflects an attentive stage of processing, and a more experience driven effects (Maiste et al., 1995; Frenck-Mestre et al., 2005). The P300 effect has been interpreted as an index of discrimination of speech stimuli by phonological information (Maiste et al., 1995; Frenck-Mestre et al., 2005; Zheng et al., 2012). Both the MMN and the P300 have been found in lexical tone processing in tone languages, but they have predominantly been examined at the level of categorical perception (Luo et al., 2006; Xi et al., 2010; Zheng et al., 2012; Yu et al., 2014, 2017).

Kung et al. (2014) examined the online interplay of tone and intonation in a larger context (rather than single word stimuli) in Cantonese Chinese. In this study, Cantonese participants were asked to perform a lexical-identification task, in which (critical) words with low tone were placed at the end of either a question or a statement. Note that questions in Chinese end with a rising intonation, and thus, the pitch contour of such words is analogous to the pitch contour of words with a high lexical tone in a regular statement sentence. Their findings indicated a low accuracy in lexical identification and a P600 effect for low tone in questions compared to the same words at the end of a statement. The P600 effect was taken as an indicator of reanalysis, when the listener resolves the conflict of two competing representations that are activated in questions ending with low tones. That is, for question-final words with a low tone, tonal and intonational information interact, and this interaction leads to a conflict between the two representations.

However, despite all these studies, previous ERP studies in tone languages have focused on lexical tones (Xi et al., 2010; Zheng et al., 2012). So far, no study has examined ERP responses when processing grammatical tone.

### The Current Study

The goal of the current study is twofold. First to address the question of whether the neural processing of temporal agreement in grammatical tone languages follows the patterns found in languages in which temporal reference is expressed through inflectional morphology, that is, through affixes. Our prediction is that there would be both similarities and differences between Akan and Indo-European languages in time reference processing. While temporal processing in Indo-European languages reflects morpho-syntactic processing, temporal processing in Akan would rely on a phono-syntactic processing, because of the presence of grammatical tone. The second goal was to examine whether brain responses for past and present habitual time violations will differ in a grammatical tone language. We compare Akan present habitual verb forms to past verb forms on the basis of the fact that the present habitual and the past verbs are most comparable as far as grammatical tone is concerned. While these are strictly marked by tone, the Akan future verbs are marked by tone in addition to the future morpheme *b*ε*/be*. Our aim is not only to measure the time reference component of Akan verbs but also to examine the possible role of tone, hence, our choice for present habitual and past verbs. Tsiwah et al. (2020) demonstrated that for Akan agrammatic speakers, in both production and comprehension, past verb forms are harder to process than present habitual verb forms. On the basis of this finding, we predict that Akan present habitual and past time violations would elicit different ERP responses. Precisely, Kaan and Swaab (2003) associate (a fronto-central) P600 with discourse level complexity. Since past time reference has been suggested to involve discourse level processing (Bastiaanse, 2013), we predict a frontocentral P600 when the parser encounters a past verb violation. In contrast, since the present habitual verb violations does not require discourse level revision, we predict that no such effect would be present. Should the P600 component be elicited, that would suggest that the expression of the time reference through grammatical tone is similar to inflectional tense morphology. That is, grammatical tone, even though superficially different from inflectional morphology, may be processed like inflectional morphology. The ERP component that is of particular interest in the present study is the P600, since this has been consistently observed in studies that have examined tense processing in other languages.

## Materials and Methods

### Participants

Twenty-seven native speakers of Akan who were residents of Amsterdam, Netherlands, participated in this experiment. Three (of the 27) participants were excluded from the ERP analysis due to excessive artifacts. All the remaining twenty-four participants (4 females and 20 males; range = 23-35 years; mean age = 24 years) were right-handed, and had normal vision and hearing. All participants had at least a high school level of education, and none had a psychology or linguistics background. Participants were multilingual (Akan, Dutch and English), with Akan being their first language. Prior to testing, we had conversations in Akan with participants to ensure native speakers were recruited. None of the participants reported any speech and/or language, neurological, psychiatric or cognitive disorders. They all signed an inform consent prior to the experiment, and each participant received €15 for their participation. This study was approved by the Research Ethical Review Committee (CETO) of the Faculties of Arts, Philosophy, and Theology and Religious Studies, University of Groningen.

### Materials and Procedure

The experimental and the filler sentences were created by using 60 and 30 suitable transitive verbs, respectively. Four variants of sentences were created for each verb. Thus, a total of 240 experimental sentences and 120 fillers were included. The experimental sentences were distributed over four conditions, as illustrated in Table 1: (1) a grammatical present condition in which a temporal adverb referring to a habitual situation matched with a present habitual verb (PresPres); (2) an ungrammatical present time condition in which a temporal adverb referring to the past mismatched with a present habitual verb (PastPres); (3) a grammatical past condition in which a past temporal adverb matched with a past verb (PastPast); (4) an ungrammatical past condition in which a present temporal adverb mismatched with a past verb (PresPast). PresPres and PastPres constituted present condition, whereas PastPast and PresPast constituted the Past condition. Da biaa (“every day) and Ennora (“yesterday”) were the only adverbials used in this study since we focused on past and present habitual in Akan.

**TABLE 1:** Examples of experimental sentences, filler sentences and content questions used in the experiment.

	Experimental Sentences			
**Condition**	**Temporal adverb**	**NP Subject**	**Target Verb**	**NP Object**

**1) PresPres**	*Da biaa*	*maame no*	*pèpá*	*famu h*
	Day every	woman the	mop:Hab	floor the

	**Every day, the woman mops the floor**			

2) *PastPres	*Ennora*,	*maame no*	*pèpá*	*famu h*
	Yesterday,	woman the	mop:Hab	floor the

	*Yesterday, the woman mops the floor			

**3) PastPast**	*Ennora*,	*maame no*	*pèpàà*	*famu h*
	Yesterday,	woman the	mop:PAST	floor the

	**Yesterday, the woman mopped the floor**			

4) *PresPast	*Da biaa*	*maame no*	*pèpàà*	*famu h*
	Day every	woman the	mop:PAST	floor the

	*^1^Every day, the woman mopped the floor			

	**Filler Sentences**			

**Congruent**	*Ennora*	*papa no*	*tènàà*	*agua no so*
	Yesterday	man the	sit:Past	chair the on

	**Yesterday, the man sat on the chair**			

**Incongruent**	*Ennora*	*papa no*	*tènàà*	*εpo no so* sea the on
	Yesterday	man the	sit:Past	

	*Yesterday the man sat on the sea			

	**Content Questions**			

	*Maame no na*	*tènàà*	*agua no so?*	
	Woman the it was	she:sit:Past	chair the on	
**Ex. Question**	Was it the woman who sat on the chair?			

*^1^In contrast to English, this sentence is ungrammatical in Akan. This was also confirmed in the acceptability ratings where Akan native speakers judged these sentences to be unacceptable. Comparison between PresPres and PastPres constituted the Present condition, whereas PastPast and PresPast constituted the Past condition.*

Since time reference violations are semantic in nature, the filler sentences, as presented in Table 1, contained equal numbers of semantically congruent and incongruent sentences for both past and present habitual. This was done in order to mask the experimental sentences from the participants. Semantic anomaly was created by choosing a noun that was not semantically plausible in combination with the event (see examples in Table 1). There were 60 semantically plausible filler sentences, and 60 semantically implausible filler sentences. Half of the fillers had present habitual verb forms and the other half past verb forms, with their corresponding temporal adverbs.

All items (both experimental and fillers) were divided into two lists, and each participant was exposed to one list. Each list contained a verb inflected for the two verb forms (present habitual and past), with one being a grammatical version, and the other an ungrammatical version, preceded by the same temporal adverb (ennora or da biaa). Thus, each participant listened to a total of 180 sentences: 120 experimental items, equally divided into 4 conditions and 30 stimuli each, and 60 filler sentences.

All sentences were spoken by a female native speaker of Akan, and were recorded in a professional recording studio, using the Audacity audio recording and editing software (Audacity: Free Audio Editor and Recorder, 2015). Only grammatical sentences were recorded. The ungrammatical sentences were created from the grammatical sentences through cross splicing, in order to avoid potential confounds such as intonation and loudness. This was achieved by cutting the temporal adverb (at the initial position of each sentence) of a grammatical sentence in a particular time frame (e.g., PresPres), and replacing it with the temporal adverb of a grammatical sentence in another time frame (e.g., PastPast) to create either a PastPres or a PresPast. Finally, volume normalization was applied to all audio files. Since recoding was done in a soundproof booth, recorded sounds files were of high quality and so there was no need to use Audacity’s built-in Noise Reduction function.

**FIGURE 1:**
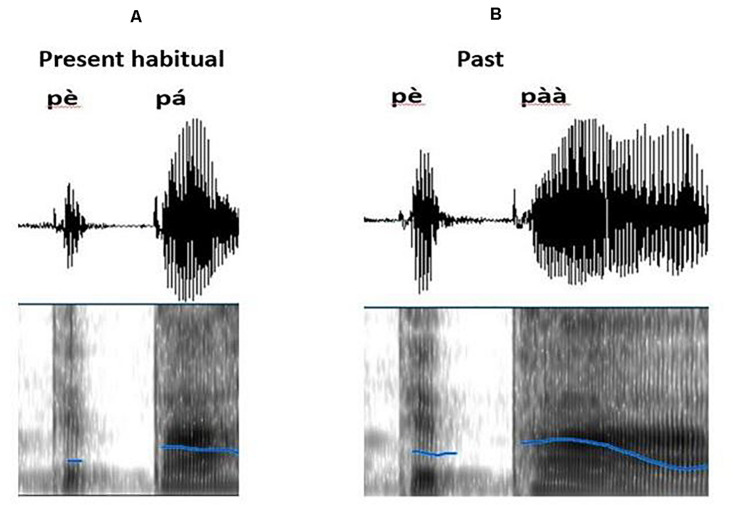
Spectrogram showing the pitch information (blue lines) of a present habitual **(A)** p p (mops) and past **(B)** p p (mopped) verbs.

A spectrographic analysis of the verb conducted on Praat software (Boersma and Weenink, 2014) showed that although the first syllable of both the present habitual and past verbs (compare examples 1 and 3 in Table 1) look identical in terms of tonal height (both have low tone), they differ in terms of duration. Past has a longer tone duration than the present habitual on both the first and the second syllables. This is illustrated on Figure 1.

#### Acceptability of Materials

All items, both experimental stimuli and fillers, were rated by 28 native Akan speakers for acceptability/unacceptability. None of the raters participated in the ERP experiment. The survey was conducted online using Qualtrics Survey software (version XM, Qualtrics., 2005), and the presentation was done auditorily. There were 360 sentences in total which were sub-divided into two lists, with 180 sentences in each list (including 60 fillers). Each participant saw only one list. Six practice sentences preceded each list. Participants were asked to judge whether each sentence they heard was acceptable or not, by indicating ‘yes’ or ‘no’. All the sentences that were included in the experimental set had a consensus rating of at least 80%. Present grammatical and present ungrammatical had acceptability ratings of 94% (sd = 1.86) and 92% (sd = 1.71), respectively, while past grammatical and past ungrammatical had 95% (sd = 1.55) and 91% (sd = 2.22), respectively. Therefore, all items were used in the main experiment.

#### Procedure

The experimental design was programed and stimuli presented using E-Prime (version 2.0, Psychology Software Tools, Inc). Participants were seated in front of a computer screen at a distance of 70 to 80 cm, in a dimly lit sound-proof cabin. All stimuli were presented auditorily, and participants had to passively listen to the sentences presented through headphones. Before each sentence, a fixation cross lasting for 500 ms appeared on the screen, prompting the participants of an incoming sentence. Randomly, once in every 3 to 5 items (either experimental or filler), a question was asked about the content of the sentence (e.g., Was it the woman who sat on the chair?). At the presentation of the random content questions, participants were required to actively respond by pressing a button on a game pad. Participants received both written and oral instructions to listen attentively to each sentence, and then answer a content question (if one was presented) by pressing a green or a red button for ‘yes’ and ‘no’ responses, respectively. After each content question, participants had 5 seconds to respond with the button press.

Prior to the experimental session, each list began with a practice session, with 6 practice sentences (including 2 content questions). This was to ensure that participants had understood the instructions. Each list was further divided into 4 blocks, with each block having a duration of 6 – 8 minutes, after which there was a break. The experiment lasted for a maximum of 40 minutes, including breaks.

#### EEG Recording

Continuous EEG data were recorded using the EEGO-Lab system (ANT Neuro Inc., Enschede, Netherlands) from 32 Ag/AgCl scalp electrodes fitted in an elastic cap (WaveGuard). Data were recorded at a sampling rate of 500 Hz, using the online common average reference, with impedances below 10 KΩ. After data recording, an initial offline data pre-processing was done using the Brain Vision Analyzer software (version2.0.4, Brain Products, GmbH, Munich, Germany), which included re-referencing to the average of the mastoid electrodes, and with highpass and lowpass filtering to cut-off frequencies below 0.1 Hz and above 30 Hz, respectively. Eye-blinks were corrected using an ICA-based eye-blink correction. The segmentation of the data was performed in epochs from 200 ms before the onset of the critical word (verb), until 1200 ms post-onset of the critical word. An automatic artifact rejection was then applied, and all epochs containing activity exceeding ± 100 μV were excluded. Overall, 6.5% of the trials contained artifacts, and, thus, were rejected. The percentage of the rejected trials did not significantly differ across conditions. Data were corrected relative to a baseline of 200 ms before the stimulus onset. The data of individual trials were exported to R (R Core Team, 2013) for further pre-processing. The data were down-sampled to 100 Hz, creating 140 time bins (per trial) of 10ms each. For analysis, we calculated the means per condition, participant, and time bin, aggregating over items.

### Statistical Analysis

For the data analysis, we used Generalized Additive Mixed Modeling (GAMM; Hastie and Tibshirani, 1990; Wood, 2017) as implemented in the package *mgcv* version 1.8-24 (Wood, 2017). The package *itsadug* 2.3 (Van Rij et al., 2017) in R version 3.4.4 (R Core Team, 2013) was used for interpretation and visualization of the analysis, and the package *eegkit* 1.0-4 for visualization of the scalp distribution. GAMM is a non-linear mixed-effects regression method, and thus, does not assume a linear relationship between the dependent variable and a covariate, but rather estimates a relationship using penalized regression splines. GAMM does not require the user to specify the shape of the regression line beforehand, but it is estimated based on the data.

The data of every single electrode in the full-time range of -200 ms to 1200 ms before and after stimulus onset were analyzed with the same model specification. The model included a non-linear effect of Time, to capture the change in ERP over time. This non-linear effect of Time interacts with condition, so that the model fits essentially four (potentially different) non-linear regression lines, capturing the change in ERP over time for each condition. Verb form (present and past) and Grammaticality (grammatical and ungrammatical) were converted into binary variables (i.e., either 0 or 1) named ISPresent (Present = 1; Past (reference) = 0), ISUngrammatical (Grammatical (reference) = 0; Ungrammatical = 1), and ISPresentUngrammatical (representing the additive difference between the difference between Present Grammatical and Present Ungrammatical in comparison to the difference between Past Grammatical and Past Ungrammatical). These binary predictors allowed us to model the non-linear differences over time between the reference level Past Grammatical and the other conditions. Table 2 shows how the treatment coding relates to the four conditions. On the top row all fixed-effects model terms are presented, with the function *s()* indicating a smooth function to fit a non-linear regression line. On the rows, the four experimental conditions are listed, and for each condition it is indicated which model terms contribute to the model’s estimation for that condition.

**TABLE 2:** Fixed-effects model terms and how they relate to each condition.

	Intercept + s (Time)	s (Time): ISPresent	s (Time):IS- Ungrammatical	s (Time): ISPresent- Ungrammatical
Past Grammatical (reference level)	1	0	0	0
Past Ungrammatical	1	0	1	0
Present Grammatical	1	1	0	0
Present Ungrammatical	1	1	1	1

For each single electrode analysis, the GAMM model included random effects for participants. For each combination of participant and condition (Tense and Grammaticality), we added a non-linear random effect with non-linear effect over time to account for variation in the time course by participants and conditions. We chose the maximal random effects structure to maximally reduce autocorrelation, which will result in the most conservative estimates (cf. van Rij et al., 2019). To account for the remaining autocorrelation in the residuals, we also included an Auto-regressive (AR1) model that corrected the confidence intervals of the model estimates accordingly. As the data were not normally distributed, we fitted the non-linear regression model with a link function for a scaled-t distribution. Although it is possible to take into account the spatial correlation of multiple electrodes in GAMMs analysis, the high computational requirements in GAMMs make this additional complexity not feasible, at present.

The model was fitted with the maximum likelihood (ML) as smoothing parameter estimation method (cf. Wieling, 2018). We used summary statistics and visualization of the model’s predictions to assess significance (cf. van Rij et al., 2019), but not model comparison. Because we included non-linear random effects for each individual time-varying event (i.e., participant-condition combination), the fixed-effect pattern was captured by the random effects when the fixed-effects smooths were excluded. As a result, the model comparison procedure did provide much information.

Although GAMMs have recently been used in a number of ERP studies on language processing (Kryuchkova et al., 2011; Baayen et al., 2015; Meulman et al., 2015; Nixon et al., 2015), it is still a relatively novel statistical method. Therefore, we also performed a more comparable ANOVA analysis as a supplementary analysis. Since the GAMM results showed that the ERP effect only started from around 400 ms and lasted until 1200 ms, the average amplitudes of the ERP waveforms in the time-windows of 400-600 ms, 600- 800 ms, 800-1000 ms and 1000–1200 ms after the onset of a stimulus were analyzed. For this analysis, electrodes were grouped according to Regions of Interest (ROIs): LA (F3, F7 & FC5), RA (F4, F8 & FC6), LC (C3 & CP5), RC (C4 & CP6), LP (O1, P3 & P7), RP (O2, P4 & P8), MA (FC1, FC2 & Fz), MC (CP1, CP2, Cz), MP (O2, POz & Pz). We ran two analyses on each time window. The first analysis was on the lateral regions of interest and consisted of the following factors: condition (present and past), grammaticality (grammatical and ungrammatical), hemisphere (left and right), and anteriority (anterior, central, and posterior). The second analysis was performed on the midline regions and included all the factors except hemisphere. In case of violation of sphericity, the Greenhouse-Geisser correction was applied.

## Results

**FIGURE 2:**
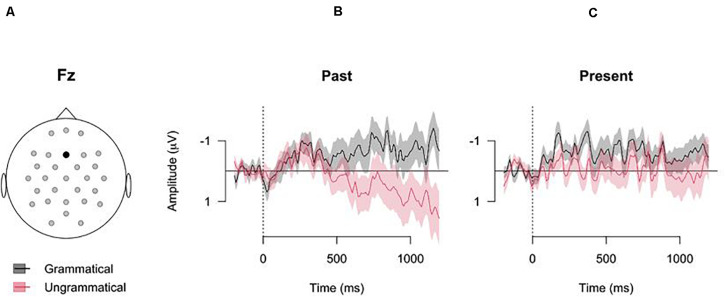
Grand averages of electrode Fz (error bars: ±1SE, based on participant means). The *x*-axes show the time (in ms) from verb onset, and the *y*-axes show the grand means of the recorded amplitudes (negative upward). **(A)** Electrode position. **(B)** Grand averages of the grammatical past tense sentences (black line) and the ungrammatical past tense sentences (red line). **(C)** Grand averages of the grammatical present tense sentences (black line) and the ungrammatical present tense sentences (red line).

Figure 2 shows the grand means for the four conditions (± standard error of participant means) for electrode Fz. For the past tense, the data shows a clear difference in the ERPs elicited by grammatical (black lines) and ungrammatical (red lines) verb forms. The amplitudes measured during the processing of ungrammatical verb forms (center panel of Figure 2) display a positive going trend from around 400 ms after verb onset, whereas the amplitudes measured during processing the grammatical verb forms stay negative. This difference is diminished for the present verb stimuli (right panel of Figure 2). See Supplementary Material 1 for the grand averages of all electrodes.

### GAMMs Analyses

The effects of Time, Verb form and Grammaticality were analyzed with GAMMs, as explained in the preceding section. Each electrode was analyzed separately. The correlation between the model fit and the data was on average 0.90 (range 0.83-0.97), and the adjusted *R*^2^ of the models (which was almost identical to the explained deviance for these models) was on average 0.81 (range 0.67-0.94), indicating an excellent fit of the data. The difference in response to the grammatical and ungrammatical verb forms in the past tense condition was found to be significant in the fronto-central electrodes: The summary statistics (see Supplementary Figure 2) indicated that the difference between grammatical past verb form and ungrammatical past verb form (captured by s(Time, by = *IsUngrammatical*)) was significant in the electrodes C3 (F(2.001, 12523.975) = 4.65; *p* < 0.01), CP1 (F(2.001, 12512.465) = 4.86; *p* < 0.01), CP2 (F(2.000, 12510.917) = 4.15; *p* = 0.016), Cz (F(2.001, 12416.119) = 4.98; *p* < 0.01), FC1 (F(2.001, 12442.790) = 3.86; *p* = 0.021), Fz (F(2.001, 12422.084) = 4.68; *p* < 0.01), and P8 (F(2.000, 12787.877) = 3.91; *p* = 0.020). The electrodes FC2 (F(2.001, 12399.588) = 2.40; *p* = 0.091) and P7 (F(2.000, 12724.398) = 2.60; *p* = 0.075) showed marginally significant effects. The difference between Past Grammatical and Present Grammatical (captured by s(Time, by = *IsPresent*), see Supplementary Material 3) was only significant for electrode POz (F(6.511, 12669.473) = 2.28; *p* = 0.022). Finally, the difference between the effect of Grammaticality in the Past verb form and in the Present verb form was significant for electrode CP1 (F(2.002, 12512.465) = 3.10; *p* = 0.045) and marginally significant for electrodes C3 (F(2.000, 12523.975) = 2.97; *p* = 0.051), CP2 (F(2.000, 12510.917) = 2.70; *p* = 0.067), and P8 (F(2.002, 12787.877) = 2.70; *p* = 0.067).

**FIGURE 3:**
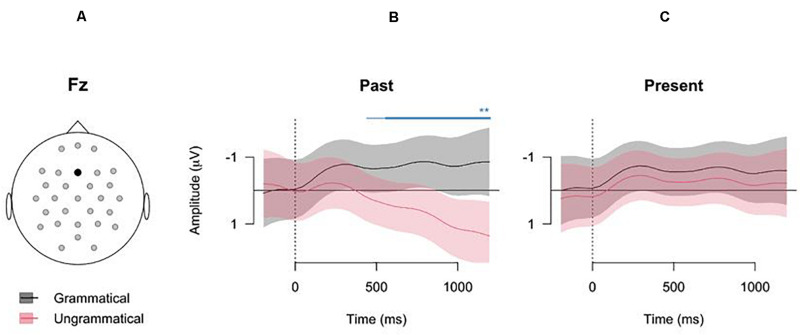
Model estimates (i.e., summed effects, with random effects excluded) for the GAMM fitting the data of electrode Fz (error bars: pointwise CI, 95%). The *x*-axes show the time (in ms) from verb onset, and the *y*-axes show the estimated amplitudes (negative upward). **(A)** Electrode position. **(B)** Odel estimates of the grammatical past tense sentences (black line) and the ungrammatical past tense sentences (red line). **(C)** Model estimates of the grammatical present tense sentences (black line) and the ungrammatical present tense sentences (red line). The blue line in the center panel indicates the time window in which the model predicts a significant difference between the two grammaticality conditions. (The thin line is the estimated difference based on pointwise confidence intervals, whereas the thick lines are the estimated differences based on the simultaneous confidence intervals).

**FIGURE 4:**
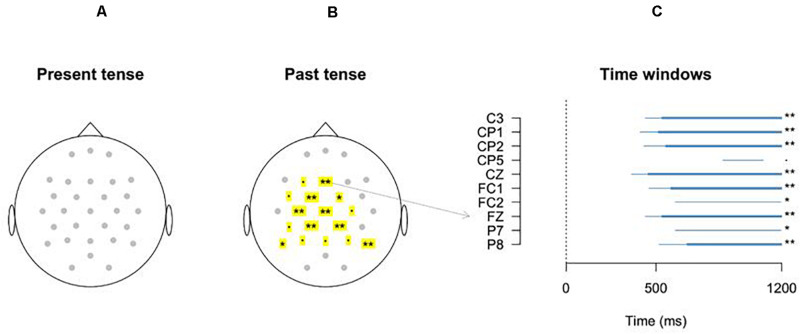
Predicted differences of the GAMM analyses per electrode. **(A,B)** For the Present tense and Past tense conditions, respectively, the significant differences between grammatical and ungrammatical verbs are indicated with significant stars: ***p < 0.001; **p < 0.01; *p<0.05; *p* < 0.1. Significant differences are only predicted for the Past tense conditions. **(C)** Represents the time windows in which the GAMM analyses predict the difference to occur. (The thin lines are the estimated differences based on pointwise confidence intervals, whereas the thick lines are the estimated differences based on the simultaneous confidence intervals).

To inspect the interaction between Time, Verb forms, and Grammaticality as estimated by the model, we visualized the summed fixed-effect predictions, as in Figure 3 for electrode Fz (see Supplementary Material 2 for model estimates for all electrodes). Note that these summed effects additionally include the intercept and its variance, but no random effects. The summed effects showed significant differences in Grammaticality for the Past verb form in the central electrodes (see Figure 3), but no significant differences in Grammaticality for the Present verb form. Most differences between the grammaticality conditions for the Past verb form started around 500 ms after verb onset (see Figure 4, Right panel) and lasted until the end of the analysis window.

### Comparable Anova Analysis

**FIGURE 5:**
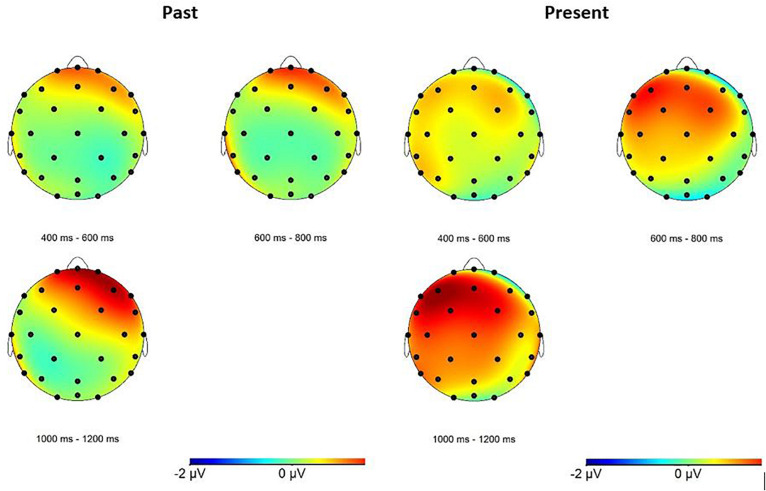
Topographic maps indicating the ERP positive effect for past and present verb violations.

Since the GAMMs results showed that the ERP effect only started from around 500 ms and lasted until 1200 ms, the average amplitudes of the ERP waveforms in this time-window after the onset of a stimulus were used for the ANOVA analysis (see Figure 5 for topographic maps). In the first time window (400-600 ms), only a four-way interaction between condition, grammaticality, hemisphere, and anteriority was close-to-significant (F (2, 46) = 3.2, p = 0.079, η_*p*_^2^ = 0.12) in the lateral analysis. However, the *post hoc* tests did not reveal any significant effect (all *p*s > 0.1).

In the following time window (600-800 ms), the only significant result was an interaction between grammaticality and anteriority in the midline (F (2, 46) = 3.65, p = 0.048, η_*p*_^2^ = 0.14). The follow-up tests showed that ungrammatical sentences elicited a more positive response in the anterior (t (23) = -2.28, p = 0.032) and central (t (23) = -2.19, p = 0.039) region.

In the third time window (800-1000 ms), there was a marginally significant effect of grammaticality (F (1, 23) = 4.132, p = 0.054, η_*p*_^2^ = 0.15), and a marginally significant three-way interaction between condition, grammaticality and anteriority (F (2, 46) = 3.27, p = 0.068, η_*p*_^2^ = 0.125), both in the midline analysis. The follow-up of the three-way interaction revealed that ungrammatical sentences elicited a more positive waveform only in the past tense condition in the anterior (t (23) = -2.46, p = 0.022) and central (t (23) = -2.91, p = 0.008) region.

In the last time window (1000-1200 ms), there was a close-to-significant effect of grammaticality (F (1, 23) = 3.05, p = 0.094, η_*p*_^2^ = 0.12) in the lateral analysis. In the midline, there was a main effect of grammaticality (F (1, 23) = 4.97, p = 0.036, η_*p*_^2^ = 1.8), as well as an interaction between condition and grammaticality (F (1, 23) = 3.51, p = 0.074, η_*p*_^2^ = 0.13). The follow-up on the latter interaction showed that ungrammatical sentences elicited a more positive waveform than grammatical sentences in the past tense condition only (t (23) = -2.85, p = 0.009).

In summary, both the GAMMs and the ANOVA analyses revealed a P600-like effect in the past verb violation condition whereas there was no such effect in the present verb violation condition. However, while the GAMM analyses was sensitive to capture the interaction between grammaticality and verb forms from around 450 ms, the ANOVA analyses could only capture this interaction from around 800 ms.

## Discussion

The current study investigated the effect of grammatical tone on the processing of temporal (dis)agreement in Akan, using event-related potentials (ERP) brain imaging technique. Our aim was, first to examine the electrophysiological processes involved in temporal (dis)agreement in a grammatical tone language, and how they relate to the morphosyntactic processing of tense reported for Indo-European languages. The second goal was to examine whether the brain responses revealed by the ERP components for past and present habitual time violations differ in a grammatical tone language. In this section, we will first discuss the ERP findings in relation to the previous ERP studies on tense in Indo-European languages (Steinhauer and Ullman et al., 2002; Baggio, 2008; Dragoy et al., 2012), and then the differences in neural mechanism that underlie past and present habitual verbs.

### Tense and Grammatical Tone in Temporal Agreement Processing

Cross-linguistics studies on temporal (dis)agreement in Indo European languages have found that tense violations elicit a centro-posterior positivity (P600), peaking around 600–900 ms, sometimes preceded by a negativity with a clear anterior distribution or with a more posterior distribution (peaking around 300–500 ms after the onset of the verb). This biphasic LAN-P600 pattern has been observed by a number of studies that looked at tense in Indo-European languages such English (Steinhauer and Ullman et al., 2002) and Dutch (Baggio, 2008). While Steinhauer and Ullman et al. (2002) took this LAN-P600 pattern as indices of morpho-syntactic processing of tense, Baggio (2008) treated these ERP components as a signature of temporal-semantic processing. Dragoy et al. (2012) replicated only the P600 ERP component in sentences where a past context was violated by a present verb forms in Dutch compared to sentences with both the context and the verb referring to present. Dragoy and colleagues found no effect when the present context was violated by past tense compared to sentences with both the context and the verb referring to past.

Our findings are that temporal disagreement in Akan also elicits a P600-like ERP component. Nonetheless, this ERP effect was evident only when temporal violations were caused by past verb forms. This positive ERP effect observed in the current study differs in some ways from the P600 observed by Steinhauer and Ullman et al. (2002) and Baggio (2008). This may be the result of differences in materials and study designs. Note, that the above-mentioned studies, with exception of Dragoy et al. (2012), only tested temporal disagreement caused by present verbs, whereas the current study tested both past and present verb violations. Furthermore, these studies measured the ERP effect on different verb forms. Therefore, the elicitation of the ERP components may not necessarily have been associated with temporal disagreement *per se*, but rather due to differences in factors such as length (or differences in forms) and frequency between the word forms that were used. However, like our study, Dragoy and colleagues’ (2012) compared true tense mismatch mismatches by using the same verb forms with differences only in the temporal contexts (e.g., *De kelner [die nu/zonet de peper maalt] krijgt geen fooi*: ‘The waiter who is now/a-moment-ago grinds the pepper doesn’t get a tip’). The fact that, in contrast to our study, Dragoy and colleagues did not find an effect when violations were caused by past verb forms will be further discussed later.

One of the ways in which the P600 component observed in the current study differs from previous reports of this component in Indo-European languages is its latency. The positivity observed in this study was earlier and lasted longer than the P600 reported by Steinhauer and Ullman et al. (2002) and Baggio (2008). In our case, the P600-like effect emerged from around 400 ms and lasted until around 1200 ms. Generally, this is consistent with the long-lasting nature of the P600 component whose effect usually extends beyond the end of the target word (see Osterhout and Mobley, 1995; Kaan and Swaab, 2003). Furthermore, the longer-lasting positive effect is consistent with the results of Dragoy et al. (2012) who also tested pure time reference violations and showed a longer lasting P600-like effect peaking around 1200 ms after stimulus onset. This suggest that pure time reference violations (such as in Dragoy et al., 2012 and the current study) evoke a longer-lasting P600 effect. However, further investigation is needed to establish this tentative interpretation.

There are two more possible explanations for the long lasting P600 latency found in this study. First, the early emergence of the P600 in the current study can be attributed to the presence of tone in general, which results in ‘phono-syntactic’ rather than the morpho-syntactic processing in Indo-European languages. Previous ERP studies that have investigated (lexical) tone processing in tonal languages have observed an early positivity (such as the P300) which has been taken to be an index of discrimination of speech stimuli by phonological information (Maiste et al., 1995; Frenck-Mestre et al., 2005; Zheng et al., 2012). Note that these studies measured lexical tone processing, and thus, the nature of the positivity may not be the same as grammatical level tone processing. Secondly, a spectrographic analysis of the verbs (e.g., past: *pèpàà* ‘mopped’; present habitual: *pèpá* ‘mops’) indicated that although the tonal height of the first syllable of both past and present habitual verbs are identical, they tend to differ in length, with the past having a relatively longer tone duration than the present habitual (see Figure 1 above). That is, the past and the present habitual do not only differ (in height and duration) at the last syllable but also (in duration) at the first syllable. This finding has not been described in the literature before (Akan is a very understudied language), nonetheless, we suggest that it is due to an assimilation process in the Akan past verbs, resulting from the prolonged tonal duration of the last syllable. Consequently, the parser recognizes two disambiguating points when processing past verbs violating present context, with the first disambiguation point occurring at an early stage of processing. Contrarily, in Indo-European languages, there is only one point of disambiguation on the target occurring at the end of the verb (Dutch: Baggio, 2008; Dragoy et al., 2012; English: Steinhauer and Ullman et al., 2002). Taken together, the latency of the P600-like ERP component in the current study portrays the uniqueness of Akan verb morphology in which tone rather than affixes is used to indicate time.

Another way in which the observed positive effect in this study differs from that found in other languages is the scalp distribution of the P600. The P600 effect elicited by the past verb violations was localized fronto-centrally, and stronger centrally, as opposed to the centro-posterior P600 effect observed in Indo-European languages (Steinhauer and Ullman et al., 2002; Baggio, 2008; Dragoy et al., 2012). These distributional differences may be due to the differences in the type of violations used in these studies. In their review of P600 studies, Hagoort et al. (1999), argue that the distribution of the P600 evoked by ambiguity resolution (reanalysis–related P600) is different from P600 elicited by pure syntactic violations (repair-related P600). Molinaro et al. (2011) reported that these distributional differences can also be associated with latency, and thus, indicating two functionally different processing stages with early (about 500 to 750 ms) and late (750 – 1000 ms) stages having a frontal distribution and a posterior distribution, respectively. Building on this, Friederici et al. (2002) found a P600 with a more fronto-central distribution for violations which were syntactically ambiguous in nature (such as garden path sentences), and a central-posterior P600 for sentences with morphosyntactic violations. While Friederici et al. (2002) attribute a fronto-central P600 to a general syntactic revision, Kaan and Swaab (2003) specifically associate it with discourse level complexity which requires revision. We will revisit the discussion on discourse level revision in the next sub-section.

### Differences in the Processing of Past and Present Habitual

The results of the current study show that the processing of present habitual and past verb forms in Akan involve different neuronal processes. While past verb violations elicited a P600-like ERP component, there was no such effect when violations were caused by present verbs. This is consistent with the dissociation found between the past and the present (habitual) in clinical population studies (Bastiaanse, 2013; Bos and Bastiaanse, 2014; Tsiwah et al., 2020). Tsiwah and colleagues (2020) demonstrated that agrammatic aphasic speakers of Akan showed a dissociation in processing past and present habitual verbs in Akan, with the former being more difficult than the latter. The differences in processing between past and present verb forms has also been reported in studies that used reaction times as a reflection of temporal reference violations (Faroqi-Shah and Dickey, 2009; Jonkers et al., 2007). This shows that regardless of how time reference is expressed, whether through affixes (as in Indo-European languages) or tone (as in Akan), the processing of present and the past times show different underlying neural processes.

However, the direction of the past and present habitual difference in the current study is not in line with what was observed by Dragoy and colleagues (2012) in Dutch, an Indo-European language. In fact, an opposite pattern was found in the current study. Dragoy and colleagues found the P600-like effect when violations were caused by present tense verbs, but there was no effect when past verb forms caused the violations. This inconsistency can be attributed to the differences in the usage of present (habitual) and past verb forms in these languages (Akan and Dutch). First, the absence of an effect in the Akan present habitual may have been caused by the extra aspectual information on top of the temporal violation itself, unlike the present tense verbs in Indo-European languages which strictly represent reference to the moment of speaking. It is not clear cut whether the Akan present habitual locates a specific event in time or is pure aspectual in nature, or both. While some researchers argue that the habitual is a present tense, since it can locate actions in the present time, indefinite time, or at all times (see Christaller, 1875:59; and also Dolphyne, 1971), others have argued that the semantic function of the habitual marker is purely aspectual (see Osam, 1994; Boadi, 2008). We speculate that the use of the Akan (present) habitual itself may have both aspectual and tense characteristics, and thus the use of the present habitual with a past tense adverb (such as yesterday) may not strictly represent a temporal violation. The time frame covered by the habitual is (usually) past, present and future, and from the perspective of aspect, it tells us that an event is either ongoing or that it is iterative. Akan uses both the present and the past habitual (represented by a clause initial particle ‘na’ indicating a past context), with the difference being whether the situation is current (still happens: present habitual) or used to happen and will not happen again (past habitual). In a way, there is a temporal delineation, in the sense that while the present habitual encompasses present, and implies both the past and the future, the past habitual encompasses only the past until the present moment. This creates a possibility to use the present habitual with ‘yesterday’. In this way, there is no real temporal violation, as the past action can be included in the span of the present habitual. Therefore, it remains a possibility that the participants understood the use of the present habitual with past tense temporal adverbs in this way. This makes it incomparable to the present tense used in Dragoy and colleagues’ (2012) study.

The presence of the P600-like effect in past verb form violations, even though is inconsistent with what was reported by Dragoy and colleagues (2012) in an Indo-European language, Dutch, this is consistent with the findings of Qiu and Zhou (2012) in Chinese and Siriboonpipattana et al. (Submitted) in Thai. For instance, Qui and Zhou found that both past adverb *cengjing* and past aspectual particle *-guo* in Mandarin Chinese elicited a P600 (preceded by N400 in the case of past aspectual adverb) when they violated future context. According to Kaan and Swaab, the P600 effect, especially when distributed fronto-centrally, is indicative of access to discourse level processing. We argue that this is the case when a violation is caused by a past verb. The Past Discourse Linking Hypothesis (PADILIH: Bastiaanse, 2013; Bastiaanse et al., 2011) claims that past time reference, whether expressed through tense and/or aspectual verb inflection requires discourse-linking. According to the PADILIH, past time reference requires a link between the time of speaking the event time, since they do not coincide, whereas for reference to the present, the time of speaking coincides with the time of the event, and hence, no discourse linking is required. As a result, processing past time reference is more complex than present time reference. In the current study, the time frame was set up by a present habitual temporal adverb, which was then violated by a past verb at a later point. According to Dillon et al. (2012), the cause of a violation is a powerful source of information that is needed for diagnosis and repair of a violation. This may suggest that the parser first recognizes the violation (past verb), and then makes an attempt to fine-tune the time frame indicated by the temporal adverb. We suggest that this process of reanalysis is what gives rise to the P600.

Overall, the existence of the P600 effect in only past violations indicate that the present habitual and past in Akan involve different neuronal processes. This is consistent with the dissociation found between the past and the present (habitual) among Akan agrammatic aphasic speakers, who showed that the former was more difficult to process than the latter.

In conclusion, the P600-like effect observed in Akan sentences with grammatical tone violations shows both similarities and differences with results from studies on the same topic in Indo-European languages and in languages that use aspectual adverbs. The similarity is the P600 that arises in all languages in at least one condition, no matter whether time reference is achieved through bound morphemes, free-standing morphemes or grammatical tone. The fact that this component is not found in all conditions, may be due to language-specific characteristics as well as methodological issues. This may also explain why a LAN is reported in some studies, but not in others.

### Limitations of the Study

To further examine the absence of an ERP component in the present habitual, an important thing to consider for future research will be to use acceptability or grammaticality judgment (after each stimulus presentation) during the experiment instead of the random content questions adopted for the current study. Although the offline grammaticality judgment task revealed that native Akan speakers judge a past context violated by a present habitual verb (eg. *Ennora, papa no hwàné ankaa*; ‘Yesterday, the man peels oranges) to be ungrammatical, this effect of ungrammaticality was not reflected in the ERP experiment. Note, however, that the Akan speakers who participated in the offline grammaticality judgment task were different from those who participated in the ERP experiment. Additionally, because the participants of the ERP experiments were all multilingual (although they were all native speakers of Akan), there is a possibility of transfer effect from their knowledge of L2 to L1. We recommend that future studies collect data about the participants L2 experience.

Furthermore, we have argued that the absence of an ERP effect in the present habitual verb form violations may be due to the extra aspectual information (such as truth proposition and state) contained in the Akan present habitual. That is, on top of the notion of present time carried by the Akan present habitual verb, there is also background eventuality (Boadi, 2008) which may overlay the effect of the present time. To resolve this, future studies can consider using the Akan present progressive (e.g., *Sesiaa papa no (è)twìtwá brood no*; ‘Right now, the man is cutting the bread’) which carries only the notion of time (an ongoing event). Although the Akan present progressive is indicated by a prefix (in addition to the tonal marking) in written form, this prefix becomes covert when used verbally. Thus, in an auditory paradigm, such as used in the current study, the use of the Akan present progressive will be comparable to some extent to the Akan past which is purely marked by tone and duration. Also, since we could not make a direct comparison between the Akan present habitual and the present tense used in the study of Dragoy et al. (2012), the use of the Akan present progressive would make a fairer comparison.

Additionally, our GAMM and ANOVA analyses indicated that while the former was able to capture the interaction effect around 500 ms, the latter could only capture this effect around 700 ms. Discussing the advantages of GAMM over ANOVA was outside the scope of this study, and thus recommend this for future studies.

Lastly, we have barely scratched the surface of tone processing in Akan. The current study focused on grammatical tone processing using Akan verbs. However, like Chinese, Akan also has lexical tone. That is, two words can be differentiated in meaning on the basis of their tonal markings (eg. *pàpá* ‘father’; *pàpà* ‘fan’). Therefore, it is interesting for future studies to systematically compare lexical and grammatical tone processing in one experimental paradigm, in the same language, and with the same participants. This will give more insight into the distinct electrophysiological mechanism(s) that underlie tone processing both at the word and sentence level.

## Data Availability Statement

The raw data supporting the conclusions of this article will be made available by the authors, without undue reservation.

## Ethics Statement

The studies involving human participants were reviewed and approved by Research Ethical Review Committee (CETO), Faculties of Arts, Philosophy, and Theology and Religious Studies, University of Groningen. The patients/participants provided their written informed consent to participate in this study.

## Author Contributions

RB, FT, and SP contributed to the conception and design of the study. FT collected the data. SP supervised data collection. JR performed data analysis. FT wrote the original draft. SP and RB supervised the writing of the manuscript. All authors contributed to the manuscript revision, read, and approved the submitted version.

## Conflict of Interest

The authors declare that the research was conducted in the absence of any commercial or financial relationships that could be construed as a potential conflict of interest.
