# Supplementary material for: Online Processing of Temporal Agreement in a Grammatical Tone Language: An ERP Study
Source: Front Psychol. 2021 May 21;12:638716. doi: 10.3389/fpsyg.2021.638716 (PMC8176019; doi:10.3389/fpsyg.2021.638716)
Supplement: Supplementary file 1 [file Data_Sheet_1.ZIP › Supplementary files/Supplementary Material 1.html]

Online processing of temporal agreement in a grammatical tone language: An ERP study. Supplementary Material 2: Grand Averages for all electrodes


# Online processing of temporal agreement in a grammatical tone language: An ERP study. Supplementary Material 2: Grand Averages for all electrodes

#### Frank Tsiwah, Roelien Bastiaanse, Jacolien van Rij and Srđan Popov

#### 3/30/2021

#Functions

```
library(plyr)
library(plotfunctions)
```

```
## Warning: package 'plotfunctions' was built under R version 3.6.1
```

#Load data

```
load("./R  output/data.rda")
head(dat)
```

```
##   Subject Condition Grammaticality Channel Time      value
## 1 Frank01 Past_gram    Grammatical     Fp1 -195 -1.5544846
## 2 Frank01 Past_gram    Grammatical     Fp1 -185  0.4002179
## 3 Frank01 Past_gram    Grammatical     Fp1 -175  1.7535096
## 4 Frank01 Past_gram    Grammatical     Fp1 -165  1.0395769
## 5 Frank01 Past_gram    Grammatical     Fp1 -155 -0.1107179
## 6 Frank01 Past_gram    Grammatical     Fp1 -145 -0.4320673
```

#AVERAGES AND DATA PLOTTING

```
#Averages
avg <- ddply(dat, c("Condition", "Time", "Channel"), summarise,
             subject.mean = mean(value, na.rm=TRUE),
             subject.se    = se(value, na.rm=TRUE))

## DATA PLOTTING

par(mfrow=c(1,2))

#Electrode F3
#Past
emptyPlot(range(avg$Time), c(-2,2), v0=0, h0=0,
          eegAxis = TRUE, main="Past: F3 electrode")
with(avg[avg$Condition=="Past_ungram" & avg$Channel=="F3",], { 
  plot_error(Time, subject.mean, subject.se, col=2, shade=TRUE)
})
with(avg[avg$Condition=="Past_gram" & avg$Channel=="F3",], { 
  plot_error(Time, subject.mean, subject.se, col=1, shade=TRUE)
})

#Present
emptyPlot(range(avg$Time), c(-2,2), v0=0, h0=0,
          eegAxis = TRUE, main="Present: F3 electrode")
with(avg[avg$Condition=="Present_ungram" & avg$Channel=="F3",], { 
  plot_error(Time, subject.mean, subject.se, col=2, shade=TRUE)
})
with(avg[avg$Condition=="Present_gram" & avg$Channel=="F3",], {  
  plot_error(Time, subject.mean, subject.se, col=1, shade=TRUE)
})
```

##Electrode Fp2

```
par(mfrow=c(1,2))
#Past
emptyPlot(range(avg$Time), c(-2,2), v0=0, h0=0,
          eegAxis = TRUE, main="Past: Fp2 electrode")
with(avg[avg$Condition=="Past_ungram" & avg$Channel=="Fp2",], { 
  plot_error(Time, subject.mean, subject.se, col=2, shade=TRUE)
})
with(avg[avg$Condition=="Past_gram" & avg$Channel=="Fp2",], { 
  plot_error(Time, subject.mean, subject.se, col=1, shade=TRUE)
})

#Present
emptyPlot(range(avg$Time), c(-2,2), v0=0, h0=0,
          eegAxis = TRUE, main="Present: Fp2 electrode")
with(avg[avg$Condition=="Present_ungram" & avg$Channel=="Fp2",], { 
  plot_error(Time, subject.mean, subject.se, col=2, shade=TRUE)
})
with(avg[avg$Condition=="Present_gram" & avg$Channel=="Fp2",], {  
  plot_error(Time, subject.mean, subject.se, col=1, shade=TRUE)
})
```

##Electrode F7

```
par(mfrow=c(1,2))
#Past
emptyPlot(range(avg$Time), c(-2,2), v0=0, h0=0,
          eegAxis = TRUE, main="Past: F7 electrode")
with(avg[avg$Condition=="Past_ungram" & avg$Channel=="F7",], { 
  plot_error(Time, subject.mean, subject.se, col=2, shade=TRUE)
})
with(avg[avg$Condition=="Past_gram" & avg$Channel=="F7",], { 
  plot_error(Time, subject.mean, subject.se, col=1, shade=TRUE)
})

#Present
emptyPlot(range(avg$Time), c(-2,2), v0=0, h0=0,
          eegAxis = TRUE, main="Present: F7 electrode")
with(avg[avg$Condition=="Present_ungram" & avg$Channel=="F7",], { 
  plot_error(Time, subject.mean, subject.se, col=2, shade=TRUE)
})
with(avg[avg$Condition=="Present_gram" & avg$Channel=="F7",], {  
  plot_error(Time, subject.mean, subject.se, col=1, shade=TRUE)
})
```

##Electrode Fp1

```
par(mfrow=c(1,2))
#Past
emptyPlot(range(avg$Time), c(-2,2), v0=0, h0=0,
          eegAxis = TRUE, main="Past: Fp1 electrode")
with(avg[avg$Condition=="Past_ungram" & avg$Channel=="Fp1",], { 
  plot_error(Time, subject.mean, subject.se, col=2, shade=TRUE)
})
with(avg[avg$Condition=="Past_gram" & avg$Channel=="Fp1",], { 
  plot_error(Time, subject.mean, subject.se, col=1, shade=TRUE)
})

#Present
emptyPlot(range(avg$Time), c(-2,2), v0=0, h0=0,
          eegAxis = TRUE, main="Present: Fp1 electrode")
with(avg[avg$Condition=="Present_ungram" & avg$Channel=="Fp1",], { 
  plot_error(Time, subject.mean, subject.se, col=2, shade=TRUE)
})
with(avg[avg$Condition=="Present_gram" & avg$Channel=="Fp1",], {  
  plot_error(Time, subject.mean, subject.se, col=1, shade=TRUE)
})
```

##Electrode F4

```
par(mfrow=c(1,2))
#Past
emptyPlot(range(avg$Time), c(-2,2), v0=0, h0=0,
          eegAxis = TRUE, main="Past: F4 electrode")
with(avg[avg$Condition=="Past_ungram" & avg$Channel=="F4",], { 
  plot_error(Time, subject.mean, subject.se, col=2, shade=TRUE)
})
with(avg[avg$Condition=="Past_gram" & avg$Channel=="F4",], { 
  plot_error(Time, subject.mean, subject.se, col=1, shade=TRUE)
})

#Present
emptyPlot(range(avg$Time), c(-2,2), v0=0, h0=0,
          eegAxis = TRUE, main="Present: F4 electrode")
with(avg[avg$Condition=="Present_ungram" & avg$Channel=="F4",], { 
  plot_error(Time, subject.mean, subject.se, col=2, shade=TRUE)
})
with(avg[avg$Condition=="Present_gram" & avg$Channel=="F4",], {  
  plot_error(Time, subject.mean, subject.se, col=1, shade=TRUE)
})
```

##Electrode F8

```
par(mfrow=c(1,2))
#Past
emptyPlot(range(avg$Time), c(-2,2), v0=0, h0=0,
          eegAxis = TRUE, main="Past: F8 electrode")
with(avg[avg$Condition=="Past_ungram" & avg$Channel=="F8",], { 
  plot_error(Time, subject.mean, subject.se, col=2, shade=TRUE)
})
with(avg[avg$Condition=="Past_gram" & avg$Channel=="F8",], { 
  plot_error(Time, subject.mean, subject.se, col=1, shade=TRUE)
})

#Present
emptyPlot(range(avg$Time), c(-2,2), v0=0, h0=0,
          eegAxis = TRUE, main="Present: F8 electrode")
with(avg[avg$Condition=="Present_ungram" & avg$Channel=="F8",], { 
  plot_error(Time, subject.mean, subject.se, col=2, shade=TRUE)
})
with(avg[avg$Condition=="Present_gram" & avg$Channel=="F8",], {  
  plot_error(Time, subject.mean, subject.se, col=1, shade=TRUE)
})
```

##Electrode Fpz

```
par(mfrow=c(1,2))
#Past
emptyPlot(range(avg$Time), c(-2,2), v0=0, h0=0,
          eegAxis = TRUE, main="Past: Fpz electrode")
with(avg[avg$Condition=="Past_ungram" & avg$Channel=="Fpz",], { 
  plot_error(Time, subject.mean, subject.se, col=2, shade=TRUE)
})
with(avg[avg$Condition=="Past_gram" & avg$Channel=="Fpz",], { 
  plot_error(Time, subject.mean, subject.se, col=1, shade=TRUE)
})

#Present
emptyPlot(range(avg$Time), c(-2,2), v0=0, h0=0,
          eegAxis = TRUE, main="Present: Fpz electrode")
with(avg[avg$Condition=="Present_ungram" & avg$Channel=="Fpz",], { 
  plot_error(Time, subject.mean, subject.se, col=2, shade=TRUE)
})
with(avg[avg$Condition=="Present_gram" & avg$Channel=="Fpz",], {  
  plot_error(Time, subject.mean, subject.se, col=1, shade=TRUE)
})
```

##Electrode FC5

```
par(mfrow=c(1,2))
#Past
emptyPlot(range(avg$Time), c(-2,2), v0=0, h0=0,
          eegAxis = TRUE, main="Past: FC5 electrode")
with(avg[avg$Condition=="Past_ungram" & avg$Channel=="FC5",], { 
  plot_error(Time, subject.mean, subject.se, col=2, shade=TRUE)
})
with(avg[avg$Condition=="Past_gram" & avg$Channel=="FC5",], { 
  plot_error(Time, subject.mean, subject.se, col=1, shade=TRUE)
})

#Present
emptyPlot(range(avg$Time), c(-2,2), v0=0, h0=0,
          eegAxis = TRUE, main="Present: FC5 electrode")
with(avg[avg$Condition=="Present_ungram" & avg$Channel=="FC5",], { 
  plot_error(Time, subject.mean, subject.se, col=2, shade=TRUE)
})
with(avg[avg$Condition=="Present_gram" & avg$Channel=="FC5",], {  
  plot_error(Time, subject.mean, subject.se, col=1, shade=TRUE)
})
```

##Electrode FC1

```
par(mfrow=c(1,2))
#Past
emptyPlot(range(avg$Time), c(-2,2), v0=0, h0=0,
          eegAxis = TRUE, main="Past: FC1 electrode")
with(avg[avg$Condition=="Past_ungram" & avg$Channel=="FC1",], { 
  plot_error(Time, subject.mean, subject.se, col=2, shade=TRUE)
})
with(avg[avg$Condition=="Past_gram" & avg$Channel=="FC1",], { 
  plot_error(Time, subject.mean, subject.se, col=1, shade=TRUE)
})

#Present
emptyPlot(range(avg$Time), c(-2,2), v0=0, h0=0,
          eegAxis = TRUE, main="Present: FC1 electrode")
with(avg[avg$Condition=="Present_ungram" & avg$Channel=="FC1",], { 
  plot_error(Time, subject.mean, subject.se, col=2, shade=TRUE)
})
with(avg[avg$Condition=="Present_gram" & avg$Channel=="FC1",], {  
  plot_error(Time, subject.mean, subject.se, col=1, shade=TRUE)
})
```

##Electrode FC2

```
par(mfrow=c(1,2))
#Past
emptyPlot(range(avg$Time), c(-2,2), v0=0, h0=0,
          eegAxis = TRUE, main="Past: FC2 electrode")
with(avg[avg$Condition=="Past_ungram" & avg$Channel=="FC2",], { 
  plot_error(Time, subject.mean, subject.se, col=2, shade=TRUE)
})
with(avg[avg$Condition=="Past_gram" & avg$Channel=="FC2",], { 
  plot_error(Time, subject.mean, subject.se, col=1, shade=TRUE)
})

#Present
emptyPlot(range(avg$Time), c(-2,2), v0=0, h0=0,
          eegAxis = TRUE, main="Present: FC2 electrode")
with(avg[avg$Condition=="Present_ungram" & avg$Channel=="FC2",], { 
  plot_error(Time, subject.mean, subject.se, col=2, shade=TRUE)
})
with(avg[avg$Condition=="Present_gram" & avg$Channel=="FC2",], {  
  plot_error(Time, subject.mean, subject.se, col=1, shade=TRUE)
})
```

##Electrode FC6

```
par(mfrow=c(1,2))
#Past
emptyPlot(range(avg$Time), c(-2,2), v0=0, h0=0,
          eegAxis = TRUE, main="Past: FC6 electrode")
with(avg[avg$Condition=="Past_ungram" & avg$Channel=="FC6",], { 
  plot_error(Time, subject.mean, subject.se, col=2, shade=TRUE)
})
with(avg[avg$Condition=="Past_gram" & avg$Channel=="FC6",], { 
  plot_error(Time, subject.mean, subject.se, col=1, shade=TRUE)
})

#Present
emptyPlot(range(avg$Time), c(-2,2), v0=0, h0=0,
          eegAxis = TRUE, main="Present: FC6 electrode")
with(avg[avg$Condition=="Present_ungram" & avg$Channel=="FC6",], { 
  plot_error(Time, subject.mean, subject.se, col=2, shade=TRUE)
})
with(avg[avg$Condition=="Present_gram" & avg$Channel=="FC6",], {  
  plot_error(Time, subject.mean, subject.se, col=1, shade=TRUE)
})
```

##Electrode T7

```
par(mfrow=c(1,2))
#Past
emptyPlot(range(avg$Time), c(-2,2), v0=0, h0=0,
          eegAxis = TRUE, main="Past: T7 electrode")
with(avg[avg$Condition=="Past_ungram" & avg$Channel=="T7",], { 
  plot_error(Time, subject.mean, subject.se, col=2, shade=TRUE)
})
with(avg[avg$Condition=="Past_gram" & avg$Channel=="T7",], { 
  plot_error(Time, subject.mean, subject.se, col=1, shade=TRUE)
})

#Present
emptyPlot(range(avg$Time), c(-2,2), v0=0, h0=0,
          eegAxis = TRUE, main="Present: T7 electrode")
with(avg[avg$Condition=="Present_ungram" & avg$Channel=="T7",], { 
  plot_error(Time, subject.mean, subject.se, col=2, shade=TRUE)
})
with(avg[avg$Condition=="Present_gram" & avg$Channel=="T7",], {  
  plot_error(Time, subject.mean, subject.se, col=1, shade=TRUE)
})
```

##Electrode C3

```
par(mfrow=c(1,2))
#Past
emptyPlot(range(avg$Time), c(-2,2), v0=0, h0=0,
          eegAxis = TRUE, main="Past: C3 electrode")
with(avg[avg$Condition=="Past_ungram" & avg$Channel=="C3",], { 
  plot_error(Time, subject.mean, subject.se, col=2, shade=TRUE)
})
with(avg[avg$Condition=="Past_gram" & avg$Channel=="C3",], { 
  plot_error(Time, subject.mean, subject.se, col=1, shade=TRUE)
})

#Present
emptyPlot(range(avg$Time), c(-2,2), v0=0, h0=0,
          eegAxis = TRUE, main="Present: C3 electrode")
with(avg[avg$Condition=="Present_ungram" & avg$Channel=="C3",], { 
  plot_error(Time, subject.mean, subject.se, col=2, shade=TRUE)
})
with(avg[avg$Condition=="Present_gram" & avg$Channel=="C3",], {  
  plot_error(Time, subject.mean, subject.se, col=1, shade=TRUE)
})
```

##Electrode Cz

```
par(mfrow=c(1,2))
#Past
emptyPlot(range(avg$Time), c(-2,2), v0=0, h0=0,
          eegAxis = TRUE, main="Past: Cz electrode")
with(avg[avg$Condition=="Past_ungram" & avg$Channel=="Cz",], { 
  plot_error(Time, subject.mean, subject.se, col=2, shade=TRUE)
})
with(avg[avg$Condition=="Past_gram" & avg$Channel=="Cz",], { 
  plot_error(Time, subject.mean, subject.se, col=1, shade=TRUE)
})

#Present
emptyPlot(range(avg$Time), c(-2,2), v0=0, h0=0,
          eegAxis = TRUE, main="Present: Cz electrode")
with(avg[avg$Condition=="Present_ungram" & avg$Channel=="Cz",], { 
  plot_error(Time, subject.mean, subject.se, col=2, shade=TRUE)
})
with(avg[avg$Condition=="Present_gram" & avg$Channel=="Cz",], {  
  plot_error(Time, subject.mean, subject.se, col=1, shade=TRUE)
})
```

##Electrode C4

```
par(mfrow=c(1,2))
#Past
emptyPlot(range(avg$Time), c(-2,2), v0=0, h0=0,
          eegAxis = TRUE, main="Past: C4 electrode")
with(avg[avg$Condition=="Past_ungram" & avg$Channel=="C4",], { 
  plot_error(Time, subject.mean, subject.se, col=2, shade=TRUE)
})
with(avg[avg$Condition=="Past_gram" & avg$Channel=="C4",], { 
  plot_error(Time, subject.mean, subject.se, col=1, shade=TRUE)
})

#Present
emptyPlot(range(avg$Time), c(-2,2), v0=0, h0=0,
          eegAxis = TRUE, main="Present: C4 electrode")
with(avg[avg$Condition=="Present_ungram" & avg$Channel=="C4",], { 
  plot_error(Time, subject.mean, subject.se, col=2, shade=TRUE)
})
with(avg[avg$Condition=="Present_gram" & avg$Channel=="C4",], {  
  plot_error(Time, subject.mean, subject.se, col=1, shade=TRUE)
})
```

##Electrode T8

```
par(mfrow=c(1,2))
#Past
emptyPlot(range(avg$Time), c(-2,2), v0=0, h0=0,
          eegAxis = TRUE, main="Past: T8 electrode")
with(avg[avg$Condition=="Past_ungram" & avg$Channel=="T8",], { 
  plot_error(Time, subject.mean, subject.se, col=2, shade=TRUE)
})
with(avg[avg$Condition=="Past_gram" & avg$Channel=="T8",], { 
  plot_error(Time, subject.mean, subject.se, col=1, shade=TRUE)
})

#Present
emptyPlot(range(avg$Time), c(-2,2), v0=0, h0=0,
          eegAxis = TRUE, main="Present: T8 electrode")
with(avg[avg$Condition=="Present_ungram" & avg$Channel=="T8",], { 
  plot_error(Time, subject.mean, subject.se, col=2, shade=TRUE)
})
with(avg[avg$Condition=="Present_gram" & avg$Channel=="T8",], {  
  plot_error(Time, subject.mean, subject.se, col=1, shade=TRUE)
})
```

##Electrode CP5

```
par(mfrow=c(1,2))
#Past
emptyPlot(range(avg$Time), c(-2,2), v0=0, h0=0,
          eegAxis = TRUE, main="Past: CP5 electrode")
with(avg[avg$Condition=="Past_ungram" & avg$Channel=="CP5",], { 
  plot_error(Time, subject.mean, subject.se, col=2, shade=TRUE)
})
with(avg[avg$Condition=="Past_gram" & avg$Channel=="CP5",], { 
  plot_error(Time, subject.mean, subject.se, col=1, shade=TRUE)
})

#Present
emptyPlot(range(avg$Time), c(-2,2), v0=0, h0=0,
          eegAxis = TRUE, main="Present: CP5 electrode")
with(avg[avg$Condition=="Present_ungram" & avg$Channel=="CP5",], { 
  plot_error(Time, subject.mean, subject.se, col=2, shade=TRUE)
})
with(avg[avg$Condition=="Present_gram" & avg$Channel=="CP5",], {  
  plot_error(Time, subject.mean, subject.se, col=1, shade=TRUE)
})
```

##Electrode CP1

```
par(mfrow=c(1,2))
#Past
emptyPlot(range(avg$Time), c(-2,2), v0=0, h0=0,
          eegAxis = TRUE, main="Past: CP1 electrode")
with(avg[avg$Condition=="Past_ungram" & avg$Channel=="CP1",], { 
  plot_error(Time, subject.mean, subject.se, col=2, shade=TRUE)
})
with(avg[avg$Condition=="Past_gram" & avg$Channel=="CP1",], { 
  plot_error(Time, subject.mean, subject.se, col=1, shade=TRUE)
})

#Present
emptyPlot(range(avg$Time), c(-2,2), v0=0, h0=0,
          eegAxis = TRUE, main="Present: CP1 electrode")
with(avg[avg$Condition=="Present_ungram" & avg$Channel=="CP1",], { 
  plot_error(Time, subject.mean, subject.se, col=2, shade=TRUE)
})
with(avg[avg$Condition=="Present_gram" & avg$Channel=="CP1",], {  
  plot_error(Time, subject.mean, subject.se, col=1, shade=TRUE)
})
```

##Electrode CP2

```
par(mfrow=c(1,2))
#Past
emptyPlot(range(avg$Time), c(-2,2), v0=0, h0=0,
          eegAxis = TRUE, main="Past: CP2 electrode")
with(avg[avg$Condition=="Past_ungram" & avg$Channel=="CP2",], { 
  plot_error(Time, subject.mean, subject.se, col=2, shade=TRUE)
})
with(avg[avg$Condition=="Past_gram" & avg$Channel=="CP2",], { 
  plot_error(Time, subject.mean, subject.se, col=1, shade=TRUE)
})

#Present
emptyPlot(range(avg$Time), c(-2,2), v0=0, h0=0,
          eegAxis = TRUE, main="Present: CP2 electrode")
with(avg[avg$Condition=="Present_ungram" & avg$Channel=="CP2",], { 
  plot_error(Time, subject.mean, subject.se, col=2, shade=TRUE)
})
with(avg[avg$Condition=="Present_gram" & avg$Channel=="CP2",], {  
  plot_error(Time, subject.mean, subject.se, col=1, shade=TRUE)
})
```

##Electrode CP6

```
par(mfrow=c(1,2))
#Past
emptyPlot(range(avg$Time), c(-2,2), v0=0, h0=0,
          eegAxis = TRUE, main="Past: CP6 electrode")
with(avg[avg$Condition=="Past_ungram" & avg$Channel=="CP6",], { 
  plot_error(Time, subject.mean, subject.se, col=2, shade=TRUE)
})
with(avg[avg$Condition=="Past_gram" & avg$Channel=="CP6",], { 
  plot_error(Time, subject.mean, subject.se, col=1, shade=TRUE)
})

#Present
emptyPlot(range(avg$Time), c(-2,2), v0=0, h0=0,
          eegAxis = TRUE, main="Present: CP6 electrode")
with(avg[avg$Condition=="Present_ungram" & avg$Channel=="CP6",], { 
  plot_error(Time, subject.mean, subject.se, col=2, shade=TRUE)
})
with(avg[avg$Condition=="Present_gram" & avg$Channel=="CP6",], {  
  plot_error(Time, subject.mean, subject.se, col=1, shade=TRUE)
})
```

##Electrode P7

```
par(mfrow=c(1,2))
#Past
emptyPlot(range(avg$Time), c(-2,2), v0=0, h0=0,
          eegAxis = TRUE, main="Past: P7 electrode")
with(avg[avg$Condition=="Past_ungram" & avg$Channel=="P7",], { 
  plot_error(Time, subject.mean, subject.se, col=2, shade=TRUE)
})
with(avg[avg$Condition=="Past_gram" & avg$Channel=="P7",], { 
  plot_error(Time, subject.mean, subject.se, col=1, shade=TRUE)
})

#Present
emptyPlot(range(avg$Time), c(-2,2), v0=0, h0=0,
          eegAxis = TRUE, main="Present: P7 electrode")
with(avg[avg$Condition=="Present_ungram" & avg$Channel=="P7",], { 
  plot_error(Time, subject.mean, subject.se, col=2, shade=TRUE)
})
with(avg[avg$Condition=="Present_gram" & avg$Channel=="P7",], {  
  plot_error(Time, subject.mean, subject.se, col=1, shade=TRUE)
})
```

##Electrode P3

```
par(mfrow=c(1,2))
#Past
emptyPlot(range(avg$Time), c(-2,2), v0=0, h0=0,
          eegAxis = TRUE, main="Past: P3 electrode")
with(avg[avg$Condition=="Past_ungram" & avg$Channel=="P3",], { 
  plot_error(Time, subject.mean, subject.se, col=2, shade=TRUE)
})
with(avg[avg$Condition=="Past_gram" & avg$Channel=="P3",], { 
  plot_error(Time, subject.mean, subject.se, col=1, shade=TRUE)
})

#Present
emptyPlot(range(avg$Time), c(-2,2), v0=0, h0=0,
          eegAxis = TRUE, main="Present: P3 electrode")
with(avg[avg$Condition=="Present_ungram" & avg$Channel=="P3",], { 
  plot_error(Time, subject.mean, subject.se, col=2, shade=TRUE)
})
with(avg[avg$Condition=="Present_gram" & avg$Channel=="P3",], {  
  plot_error(Time, subject.mean, subject.se, col=1, shade=TRUE)
})
```

##Electrode Pz

```
par(mfrow=c(1,2))
#Past
emptyPlot(range(avg$Time), c(-2,2), v0=0, h0=0,
          eegAxis = TRUE, main="Past: Pz electrode")
with(avg[avg$Condition=="Past_ungram" & avg$Channel=="Pz",], { 
  plot_error(Time, subject.mean, subject.se, col=2, shade=TRUE)
})
with(avg[avg$Condition=="Past_gram" & avg$Channel=="Pz",], { 
  plot_error(Time, subject.mean, subject.se, col=1, shade=TRUE)
})

#Present
emptyPlot(range(avg$Time), c(-2,2), v0=0, h0=0,
          eegAxis = TRUE, main="Present: Pz electrode")
with(avg[avg$Condition=="Present_ungram" & avg$Channel=="Pz",], { 
  plot_error(Time, subject.mean, subject.se, col=2, shade=TRUE)
})
with(avg[avg$Condition=="Present_gram" & avg$Channel=="Pz",], {  
  plot_error(Time, subject.mean, subject.se, col=1, shade=TRUE)
})
```

##Electrode P4

```
par(mfrow=c(1,2))
#Past
emptyPlot(range(avg$Time), c(-2,2), v0=0, h0=0,
          eegAxis = TRUE, main="Past: P4 electrode")
with(avg[avg$Condition=="Past_ungram" & avg$Channel=="P4",], { 
  plot_error(Time, subject.mean, subject.se, col=2, shade=TRUE)
})
with(avg[avg$Condition=="Past_gram" & avg$Channel=="P4",], { 
  plot_error(Time, subject.mean, subject.se, col=1, shade=TRUE)
})

#Present
emptyPlot(range(avg$Time), c(-2,2), v0=0, h0=0,
          eegAxis = TRUE, main="Present: P4 electrode")
with(avg[avg$Condition=="Present_ungram" & avg$Channel=="P4",], { 
  plot_error(Time, subject.mean, subject.se, col=2, shade=TRUE)
})
with(avg[avg$Condition=="Present_gram" & avg$Channel=="P4",], {  
  plot_error(Time, subject.mean, subject.se, col=1, shade=TRUE)
})
```

##Electrode P8

```
par(mfrow=c(1,2))
#Past
emptyPlot(range(avg$Time), c(-2,2), v0=0, h0=0,
          eegAxis = TRUE, main="Past: P8 electrode")
with(avg[avg$Condition=="Past_ungram" & avg$Channel=="P8",], { 
  plot_error(Time, subject.mean, subject.se, col=2, shade=TRUE)
})
with(avg[avg$Condition=="Past_gram" & avg$Channel=="P8",], { 
  plot_error(Time, subject.mean, subject.se, col=1, shade=TRUE)
})

#Present
emptyPlot(range(avg$Time), c(-2,2), v0=0, h0=0,
          eegAxis = TRUE, main="Present: P8 electrode")
with(avg[avg$Condition=="Present_ungram" & avg$Channel=="P8",], { 
  plot_error(Time, subject.mean, subject.se, col=2, shade=TRUE)
})
with(avg[avg$Condition=="Present_gram" & avg$Channel=="P8",], {  
  plot_error(Time, subject.mean, subject.se, col=1, shade=TRUE)
})
```

##Electrode POz

```
par(mfrow=c(1,2))
#Past
emptyPlot(range(avg$Time), c(-2,2), v0=0, h0=0,
          eegAxis = TRUE, main="Past: POz electrode")
with(avg[avg$Condition=="Past_ungram" & avg$Channel=="POz",], { 
  plot_error(Time, subject.mean, subject.se, col=2, shade=TRUE)
})
with(avg[avg$Condition=="Past_gram" & avg$Channel=="POz",], { 
  plot_error(Time, subject.mean, subject.se, col=1, shade=TRUE)
})

#Present
emptyPlot(range(avg$Time), c(-2,2), v0=0, h0=0,
          eegAxis = TRUE, main="Present: POz electrode")
with(avg[avg$Condition=="Present_ungram" & avg$Channel=="POz",], { 
  plot_error(Time, subject.mean, subject.se, col=2, shade=TRUE)
})
with(avg[avg$Condition=="Present_gram" & avg$Channel=="POz",], {  
  plot_error(Time, subject.mean, subject.se, col=1, shade=TRUE)
})
```

##Electrode O1

```
par(mfrow=c(1,2))
#Past
emptyPlot(range(avg$Time), c(-2,2), v0=0, h0=0,
          eegAxis = TRUE, main="Past: O1 electrode")
with(avg[avg$Condition=="Past_ungram" & avg$Channel=="O1",], { 
  plot_error(Time, subject.mean, subject.se, col=2, shade=TRUE)
})
with(avg[avg$Condition=="Past_gram" & avg$Channel=="O1",], { 
  plot_error(Time, subject.mean, subject.se, col=1, shade=TRUE)
})

#Present
emptyPlot(range(avg$Time), c(-2,2), v0=0, h0=0,
          eegAxis = TRUE, main="Present: O1 electrode")
with(avg[avg$Condition=="Present_ungram" & avg$Channel=="O1",], { 
  plot_error(Time, subject.mean, subject.se, col=2, shade=TRUE)
})
with(avg[avg$Condition=="Present_gram" & avg$Channel=="O1",], {  
  plot_error(Time, subject.mean, subject.se, col=1, shade=TRUE)
})
```

##Electrode O2

```
par(mfrow=c(1,2))
#Past
emptyPlot(range(avg$Time), c(-2,2), v0=0, h0=0,
          eegAxis = TRUE, main="Past: O2 electrode")
with(avg[avg$Condition=="Past_ungram" & avg$Channel=="O2",], { 
  plot_error(Time, subject.mean, subject.se, col=2, shade=TRUE)
})
with(avg[avg$Condition=="Past_gram" & avg$Channel=="O2",], { 
  plot_error(Time, subject.mean, subject.se, col=1, shade=TRUE)
})

#Present
emptyPlot(range(avg$Time), c(-2,2), v0=0, h0=0,
          eegAxis = TRUE, main="Present: O2 electrode")
with(avg[avg$Condition=="Present_ungram" & avg$Channel=="O2",], { 
  plot_error(Time, subject.mean, subject.se, col=2, shade=TRUE)
})
with(avg[avg$Condition=="Present_gram" & avg$Channel=="O2",], {  
  plot_error(Time, subject.mean, subject.se, col=1, shade=TRUE)
})
```
