# Supplementary material for: Online Processing of Temporal Agreement in a Grammatical Tone Language: An ERP Study
Source: Front Psychol. 2021 May 21;12:638716. doi: 10.3389/fpsyg.2021.638716 (PMC8176019; doi:10.3389/fpsyg.2021.638716)
Supplement: Supplementary file 1 [file Data_Sheet_1.ZIP › Supplementary files/Supplementary Material 2.html]

Online processing of temporal agreement in a grammatical tone language: An ERP study. Supplementary Material 2: GAMMs Analysis for all electrodes


# Online processing of temporal agreement in a grammatical tone language: An ERP study. Supplementary Material 2: GAMMs Analysis for all electrodes

#### Frank Tsiwah, Roelien Bastiaanse, Jacolien van Rij and Srđan Popov

#### 3/30/2021

#Functions

```
library(plotfunctions) # for descriptives
```

```
## Warning: package 'plotfunctions' was built under R version 3.6.1
```

```
library(plyr)
library(mgcv) #for GAMM analyses
```

```
## Warning: package 'mgcv' was built under R version 3.6.1
```

```
## Loading required package: nlme
```

```
## Warning: package 'nlme' was built under R version 3.6.3
```

```
## This is mgcv 1.8-31. For overview type 'help("mgcv-package")'.
```

```
library(itsadug) # for GAMM plotting
```

```
## Warning: package 'itsadug' was built under R version 3.6.1
```

```
## Loaded package itsadug 2.3 (see 'help("itsadug")' ).
```

#Load data

```
load("./R  output/data_gamms.rda")
head(dat)
```

```
##   Subject Condition Grammaticality Channel Time      value Tense ISPresent
## 1 Frank01 Past_gram    Grammatical     Fp1 -195 -1.5544846  Past         0
## 2 Frank01 Past_gram    Grammatical     Fp1 -185  0.4002179  Past         0
## 3 Frank01 Past_gram    Grammatical     Fp1 -175  1.7535096  Past         0
## 4 Frank01 Past_gram    Grammatical     Fp1 -165  1.0395769  Past         0
## 5 Frank01 Past_gram    Grammatical     Fp1 -155 -0.1107179  Past         0
## 6 Frank01 Past_gram    Grammatical     Fp1 -145 -0.4320673  Past         0
##   ISUngrammatical ISPresentUngrammatical
## 1               0                      0
## 2               0                      0
## 3               0                      0
## 4               0                      0
## 5               0                      0
## 6               0                      0
```

#Subsetting data with electrode: Cz

```
subdat <- droplevels(dat[dat$Channel== "Cz" & dat$Condition %in% c("Past_gram", "Past_ungram", "Present_gram", "Present_ungram"),]) 
head(subdat)
```

```
##      Subject Condition Grammaticality Channel Time       value Tense
## 1961 Frank01 Past_gram    Grammatical      Cz -195 -0.08246923  Past
## 1962 Frank01 Past_gram    Grammatical      Cz -185  0.62334615  Past
## 1963 Frank01 Past_gram    Grammatical      Cz -175  1.08404808  Past
## 1964 Frank01 Past_gram    Grammatical      Cz -165  0.58960000  Past
## 1965 Frank01 Past_gram    Grammatical      Cz -155  0.02027564  Past
## 1966 Frank01 Past_gram    Grammatical      Cz -145 -0.47047115  Past
##      ISPresent ISUngrammatical ISPresentUngrammatical
## 1961         0               0                      0
## 1962         0               0                      0
## 1963         0               0                      0
## 1964         0               0                      0
## 1965         0               0                      0
## 1966         0               0                      0
```

## Create EVENT (unique combination of Subject and Items)for random effects

```
subdat$Event <- interaction(subdat$Subject, subdat$Condition)

#   Determine starting point for each time series
subdat <- start_event(subdat,column = "Time",event = "Event")

# Make sure variables are factors
subdat$Condition <- as.factor(subdat$Condition) 
subdat$Subject <- as.factor(subdat$Subject)
```

# GAMM ANALYSIS

## Only two models are shown here for the sake of simplicity.

## The first model (Mod 1) was the model from which rho was calculated for Mod 2

## Note that several models were ran and compared before arriving at these models

```
Mod1 <- bam(value ~ s(Time, k=20) 
            + s(Time, by=ISPresent, k=20)
            + s(Time, by=ISUngrammatical, k=20)
            + s(Time, by=ISPresentUngrammatical, k=20)
            + s(Time,Event, bs='fs', m=1, k=20),
            data=subdat, discrete = TRUE, family="scat")
```

```
## Warning in gam.side(sm, X, tol = .Machine$double.eps^0.5): model has
## repeated 1-d smooths of same variable.
```

```
## Calculate rho value to account for autocorrelation in data.
myrho <- start_value_rho(Mod1)

#Fit a Model including rho
Mod2 <- bam(value ~ s(Time, k=20) 
            + s(Time, by=ISPresent, k=20)
            + s(Time, by=ISUngrammatical, k=20)
            + s(Time, by=ISPresentUngrammatical, k=20)
            + s(Time,Event, bs='fs', m=1, k=20),
            data=subdat, discrete = TRUE, family="scat", 
            AR.start=subdat$start.event, rho=myrho)
```

```
## Warning in gam.side(sm, X, tol = .Machine$double.eps^0.5): model has
## repeated 1-d smooths of same variable.
```

```
#Plot model residuals
qqnorm(resid(Mod2))
qqline(resid(Mod2))
```

#PLOTTING OF GAM model

```
par(mfcol =c(2,2))

#Plotting Past
##Plot past grammatical
plot_smooth(Mod2, view="Time", rm.ranef=TRUE, 
            cond=list(ISPresent=0, ISUngrammatical=0,ISPresentUngrammatical=0),
            col=1, xpd=TRUE, eegAxis = TRUE, ylim = c(2, -2), main="Past: Cz")
```

```
## Summary:
##  * Time : numeric predictor; with 30 values ranging from -195.000000 to 1195.000000. 
##  * ISPresent : numeric predictor; set to the value(s): 0. 
##  * ISUngrammatical : numeric predictor; set to the value(s): 0. 
##  * ISPresentUngrammatical : numeric predictor; set to the value(s): 0. 
##  * Event : factor; set to the value(s): Frank01.Past_gram. (Might be canceled as random effect, check below.) 
##  * NOTE : The following random effects columns are canceled: s(Time,Event)
##
```

```
##Plot past ungrammatical
plot_smooth(Mod2, view="Time", rm.ranef=TRUE, 
            cond=list(ISPresent=0, ISUngrammatical=1,ISPresentUngrammatical=0),
            h0=0,add=TRUE, v0=0,col=2)
```

```
## Summary:
##  * Time : numeric predictor; with 30 values ranging from -195.000000 to 1195.000000. 
##  * ISPresent : numeric predictor; set to the value(s): 0. 
##  * ISUngrammatical : numeric predictor; set to the value(s): 1. 
##  * ISPresentUngrammatical : numeric predictor; set to the value(s): 0. 
##  * Event : factor; set to the value(s): Frank01.Past_gram. (Might be canceled as random effect, check below.) 
##  * NOTE : The following random effects columns are canceled: s(Time,Event)
##
```

```
legend_margin('bottomleft', legend=c("grammatical", "ungrammatical"),
       fill=c(alpha(1), alpha(2)), border= c(alpha(1), alpha(2)),
         lwd=1.5, col=c(1,2), seg.len=1, merge=TRUE,
         bty='n')


## Plot differences (i.e. where the grammatical vs ungrammatical lines differ in this case for past)
plot_diff(Mod2, view="Time", cond=list(ISPresent=0, ISPresentUngrammatical=0), 
          comp=list(ISUngrammatical=c(0,1)), 
          rm.ranef = TRUE,
          main="Difference in Past grammaticality")
```

```
## Summary:
##  * Time : numeric predictor; with 100 values ranging from -195.000000 to 1195.000000. 
##  * ISPresent : numeric predictor; set to the value(s): 0. 
##  * ISPresentUngrammatical : numeric predictor; set to the value(s): 0. 
##  * Event : factor; set to the value(s): Frank01.Past_gram. (Might be canceled as random effect, check below.) 
##  * NOTE : The following random effects columns are canceled: s(Time,Event)
##
```

```
## 
## Time window(s) of significant difference(s):
##  380.656566 - 1195.000000
```

```
#Plotting for Present
##Plot present grammatical
plot_smooth(Mod2, view="Time", rm.ranef=TRUE, 
            cond=list(ISPresent=1, ISUngrammatical=0,ISPresentUngrammatical=0),
            col=1, xpd=TRUE, eegAxis = TRUE, ylim = c(2, -2), main="Present: Cz")
```

```
## Summary:
##  * Time : numeric predictor; with 30 values ranging from -195.000000 to 1195.000000. 
##  * ISPresent : numeric predictor; set to the value(s): 1. 
##  * ISUngrammatical : numeric predictor; set to the value(s): 0. 
##  * ISPresentUngrammatical : numeric predictor; set to the value(s): 0. 
##  * Event : factor; set to the value(s): Frank01.Past_gram. (Might be canceled as random effect, check below.) 
##  * NOTE : The following random effects columns are canceled: s(Time,Event)
##
```

```
##Plot present ungrammatical
plot_smooth(Mod2, view="Time", rm.ranef=TRUE, 
            cond=list(ISPresent=1, ISUngrammatical=1,ISPresentUngrammatical=1),
            add=TRUE, col=2, xpd=TRUE, main = "Present")
```

```
## Summary:
##  * Time : numeric predictor; with 30 values ranging from -195.000000 to 1195.000000. 
##  * ISPresent : numeric predictor; set to the value(s): 1. 
##  * ISUngrammatical : numeric predictor; set to the value(s): 1. 
##  * ISPresentUngrammatical : numeric predictor; set to the value(s): 1. 
##  * Event : factor; set to the value(s): Frank01.Past_gram. (Might be canceled as random effect, check below.) 
##  * NOTE : The following random effects columns are canceled: s(Time,Event)
##
```

```
## Plot differences (i.e. where the grammatical vs ungrammatical lines differ in this case for present)
plot_diff(Mod2, view="Time", cond=list(ISPresent=1), 
          comp=list(ISUngrammatical=c(0,1), ISPresentUngrammatical=c(0,1)), 
          rm.ranef = TRUE,
          main="Difference in Present grammaticality")
```

```
## Summary:
##  * Time : numeric predictor; with 100 values ranging from -195.000000 to 1195.000000. 
##  * ISPresent : numeric predictor; set to the value(s): 1. 
##  * Event : factor; set to the value(s): Frank01.Past_gram. (Might be canceled as random effect, check below.) 
##  * NOTE : The following random effects columns are canceled: s(Time,Event)
##
```

```
## 
## Difference is not significant.
```

#Subsetting data with electrode: Pz

```
subdat <- droplevels(dat[dat$Channel== "Pz" & dat$Condition %in% c("Past_gram", "Past_ungram", "Present_gram", "Present_ungram"),]) 
head(subdat)
```

```
##      Subject Condition Grammaticality Channel Time       value Tense
## 3221 Frank01 Past_gram    Grammatical      Pz -195  0.07748462  Past
## 3222 Frank01 Past_gram    Grammatical      Pz -185  0.62703846  Past
## 3223 Frank01 Past_gram    Grammatical      Pz -175  0.45320192  Past
## 3224 Frank01 Past_gram    Grammatical      Pz -165  0.07462308  Past
## 3225 Frank01 Past_gram    Grammatical      Pz -155 -0.06358333  Past
## 3226 Frank01 Past_gram    Grammatical      Pz -145 -0.24814423  Past
##      ISPresent ISUngrammatical ISPresentUngrammatical
## 3221         0               0                      0
## 3222         0               0                      0
## 3223         0               0                      0
## 3224         0               0                      0
## 3225         0               0                      0
## 3226         0               0                      0
```

## Create EVENT (unique combination of Subject and Items)for random effects

```
subdat$Event <- interaction(subdat$Subject, subdat$Condition)

#   Determine starting point for each time series
subdat <- start_event(subdat,column = "Time",event = "Event")

# Make sure variables are factors
subdat$Condition <- as.factor(subdat$Condition) 
subdat$Subject <- as.factor(subdat$Subject)
```

# GAMM ANALYSIS

```
Mod1 <- bam(value ~ s(Time, k=20) 
            + s(Time, by=ISPresent, k=20)
            + s(Time, by=ISUngrammatical, k=20)
            + s(Time, by=ISPresentUngrammatical, k=20)
            + s(Time,Event, bs='fs', m=1, k=20),
            data=subdat, discrete = TRUE, family="scat")
```

```
## Warning in gam.side(sm, X, tol = .Machine$double.eps^0.5): model has
## repeated 1-d smooths of same variable.
```

```
## Calculate rho value to account for autocorrelation in data.
myrho <- start_value_rho(Mod1)

#Fit a Model including rho
Mod2 <- bam(value ~ s(Time, k=20) 
            + s(Time, by=ISPresent, k=20)
            + s(Time, by=ISUngrammatical, k=20)
            + s(Time, by=ISPresentUngrammatical, k=20)
            + s(Time,Event, bs='fs', m=1, k=20),
            data=subdat, discrete = TRUE, family="scat", 
            AR.start=subdat$start.event, rho=myrho)
```

```
## Warning in gam.side(sm, X, tol = .Machine$double.eps^0.5): model has
## repeated 1-d smooths of same variable.
```

```
#Plot model residuals
qqnorm(resid(Mod2))
qqline(resid(Mod2))
```

#PLOTTING OF GAM model

```
par(mfcol =c(2,2))

#Plotting Past
##Plot past grammatical
plot_smooth(Mod2, view="Time", rm.ranef=TRUE, 
            cond=list(ISPresent=0, ISUngrammatical=0,ISPresentUngrammatical=0),
            col=1, xpd=TRUE, eegAxis = TRUE, ylim = c(2, -2), main="Past: Pz")
```

```
## Summary:
##  * Time : numeric predictor; with 30 values ranging from -195.000000 to 1195.000000. 
##  * ISPresent : numeric predictor; set to the value(s): 0. 
##  * ISUngrammatical : numeric predictor; set to the value(s): 0. 
##  * ISPresentUngrammatical : numeric predictor; set to the value(s): 0. 
##  * Event : factor; set to the value(s): Frank01.Past_gram. (Might be canceled as random effect, check below.) 
##  * NOTE : The following random effects columns are canceled: s(Time,Event)
##
```

```
##Plot past ungrammatical
plot_smooth(Mod2, view="Time", rm.ranef=TRUE, 
            cond=list(ISPresent=0, ISUngrammatical=1,ISPresentUngrammatical=0),
            h0=0,add=TRUE, v0=0,col=2)
```

```
## Summary:
##  * Time : numeric predictor; with 30 values ranging from -195.000000 to 1195.000000. 
##  * ISPresent : numeric predictor; set to the value(s): 0. 
##  * ISUngrammatical : numeric predictor; set to the value(s): 1. 
##  * ISPresentUngrammatical : numeric predictor; set to the value(s): 0. 
##  * Event : factor; set to the value(s): Frank01.Past_gram. (Might be canceled as random effect, check below.) 
##  * NOTE : The following random effects columns are canceled: s(Time,Event)
##
```

```
legend_margin('bottomleft', legend=c("grammatical", "ungrammatical"),
       fill=c(alpha(1), alpha(2)), border= c(alpha(1), alpha(2)),
         lwd=1.5, col=c(1,2), seg.len=1, merge=TRUE,
         bty='n')


## Plot differences (i.e. where the grammatical vs ungrammatical lines differ in this case for past)
plot_diff(Mod2, view="Time", cond=list(ISPresent=0, ISPresentUngrammatical=0), 
          comp=list(ISUngrammatical=c(0,1)), 
          rm.ranef = TRUE,
          main="Difference in Past grammaticality")
```

```
## Summary:
##  * Time : numeric predictor; with 100 values ranging from -195.000000 to 1195.000000. 
##  * ISPresent : numeric predictor; set to the value(s): 0. 
##  * ISPresentUngrammatical : numeric predictor; set to the value(s): 0. 
##  * Event : factor; set to the value(s): Frank01.Past_gram. (Might be canceled as random effect, check below.) 
##  * NOTE : The following random effects columns are canceled: s(Time,Event)
##
```

```
## 
## Difference is not significant.
```

```
#Plotting for Present
##Plot present grammatical
plot_smooth(Mod2, view="Time", rm.ranef=TRUE, 
            cond=list(ISPresent=1, ISUngrammatical=0,ISPresentUngrammatical=0),
            col=1, xpd=TRUE, eegAxis = TRUE, ylim = c(2, -2), main="Present: Pz")
```

```
## Summary:
##  * Time : numeric predictor; with 30 values ranging from -195.000000 to 1195.000000. 
##  * ISPresent : numeric predictor; set to the value(s): 1. 
##  * ISUngrammatical : numeric predictor; set to the value(s): 0. 
##  * ISPresentUngrammatical : numeric predictor; set to the value(s): 0. 
##  * Event : factor; set to the value(s): Frank01.Past_gram. (Might be canceled as random effect, check below.) 
##  * NOTE : The following random effects columns are canceled: s(Time,Event)
##
```

```
##Plot present ungrammatical
plot_smooth(Mod2, view="Time", rm.ranef=TRUE, 
            cond=list(ISPresent=1, ISUngrammatical=1,ISPresentUngrammatical=1),
            add=TRUE, col=2, xpd=TRUE, main = "Present")
```

```
## Summary:
##  * Time : numeric predictor; with 30 values ranging from -195.000000 to 1195.000000. 
##  * ISPresent : numeric predictor; set to the value(s): 1. 
##  * ISUngrammatical : numeric predictor; set to the value(s): 1. 
##  * ISPresentUngrammatical : numeric predictor; set to the value(s): 1. 
##  * Event : factor; set to the value(s): Frank01.Past_gram. (Might be canceled as random effect, check below.) 
##  * NOTE : The following random effects columns are canceled: s(Time,Event)
##
```

```
## Plot differences (i.e. where the grammatical vs ungrammatical lines differ in this case for present)
plot_diff(Mod2, view="Time", cond=list(ISPresent=1), 
          comp=list(ISUngrammatical=c(0,1), ISPresentUngrammatical=c(0,1)), 
          rm.ranef = TRUE,
          main="Difference in Present grammaticality")
```

```
## Summary:
##  * Time : numeric predictor; with 100 values ranging from -195.000000 to 1195.000000. 
##  * ISPresent : numeric predictor; set to the value(s): 1. 
##  * Event : factor; set to the value(s): Frank01.Past_gram. (Might be canceled as random effect, check below.) 
##  * NOTE : The following random effects columns are canceled: s(Time,Event)
##
```

```
## 
## Difference is not significant.
```

#Subsetting data with electrode: C3

```
subdat <- droplevels(dat[dat$Channel== "C3" & dat$Condition %in% c("Past_gram", "Past_ungram", "Present_gram", "Present_ungram"),]) 
head(subdat)
```

```
##      Subject Condition Grammaticality Channel Time      value Tense
## 1821 Frank01 Past_gram    Grammatical      C3 -195 0.12136923  Past
## 1822 Frank01 Past_gram    Grammatical      C3 -185 0.76665385  Past
## 1823 Frank01 Past_gram    Grammatical      C3 -175 1.36173077  Past
## 1824 Frank01 Past_gram    Grammatical      C3 -165 1.00170000  Past
## 1825 Frank01 Past_gram    Grammatical      C3 -155 0.38646795  Past
## 1826 Frank01 Past_gram    Grammatical      C3 -145 0.08028846  Past
##      ISPresent ISUngrammatical ISPresentUngrammatical
## 1821         0               0                      0
## 1822         0               0                      0
## 1823         0               0                      0
## 1824         0               0                      0
## 1825         0               0                      0
## 1826         0               0                      0
```

## Create EVENT (unique combination of Subject and Items)for random effects

```
subdat$Event <- interaction(subdat$Subject, subdat$Condition)

#   Determine starting point for each time series
subdat <- start_event(subdat,column = "Time",event = "Event")

# Make sure variables are factors
subdat$Condition <- as.factor(subdat$Condition) 
subdat$Subject <- as.factor(subdat$Subject)
```

# GAMM ANALYSIS

```
Mod1 <- bam(value ~ s(Time, k=20) 
            + s(Time, by=ISPresent, k=20)
            + s(Time, by=ISUngrammatical, k=20)
            + s(Time, by=ISPresentUngrammatical, k=20)
            + s(Time,Event, bs='fs', m=1, k=20),
            data=subdat, discrete = TRUE, family="scat")
```

```
## Warning in gam.side(sm, X, tol = .Machine$double.eps^0.5): model has
## repeated 1-d smooths of same variable.
```

```
## Calculate rho value to account for autocorrelation in data.
myrho <- start_value_rho(Mod1)

#Fit a Model including rho
Mod2 <- bam(value ~ s(Time, k=20) 
            + s(Time, by=ISPresent, k=20)
            + s(Time, by=ISUngrammatical, k=20)
            + s(Time, by=ISPresentUngrammatical, k=20)
            + s(Time,Event, bs='fs', m=1, k=20),
            data=subdat, discrete = TRUE, family="scat", 
            AR.start=subdat$start.event, rho=myrho)
```

```
## Warning in gam.side(sm, X, tol = .Machine$double.eps^0.5): model has
## repeated 1-d smooths of same variable.
```

```
#Plot model residuals
qqnorm(resid(Mod2))
qqline(resid(Mod2))
```

#PLOTTING OF GAM model

```
par(mfcol =c(2,2))

#Plotting Past
##Plot past grammatical
plot_smooth(Mod2, view="Time", rm.ranef=TRUE, 
            cond=list(ISPresent=0, ISUngrammatical=0,ISPresentUngrammatical=0),
            col=1, xpd=TRUE, eegAxis = TRUE, ylim = c(2, -2), main="Past: C3")
```

```
## Summary:
##  * Time : numeric predictor; with 30 values ranging from -195.000000 to 1195.000000. 
##  * ISPresent : numeric predictor; set to the value(s): 0. 
##  * ISUngrammatical : numeric predictor; set to the value(s): 0. 
##  * ISPresentUngrammatical : numeric predictor; set to the value(s): 0. 
##  * Event : factor; set to the value(s): Frank01.Past_gram. (Might be canceled as random effect, check below.) 
##  * NOTE : The following random effects columns are canceled: s(Time,Event)
##
```

```
##Plot past ungrammatical
plot_smooth(Mod2, view="Time", rm.ranef=TRUE, 
            cond=list(ISPresent=0, ISUngrammatical=1,ISPresentUngrammatical=0),
            h0=0,add=TRUE, v0=0,col=2)
```

```
## Summary:
##  * Time : numeric predictor; with 30 values ranging from -195.000000 to 1195.000000. 
##  * ISPresent : numeric predictor; set to the value(s): 0. 
##  * ISUngrammatical : numeric predictor; set to the value(s): 1. 
##  * ISPresentUngrammatical : numeric predictor; set to the value(s): 0. 
##  * Event : factor; set to the value(s): Frank01.Past_gram. (Might be canceled as random effect, check below.) 
##  * NOTE : The following random effects columns are canceled: s(Time,Event)
##
```

```
legend_margin('bottomleft', legend=c("grammatical", "ungrammatical"),
       fill=c(alpha(1), alpha(2)), border= c(alpha(1), alpha(2)),
         lwd=1.5, col=c(1,2), seg.len=1, merge=TRUE,
         bty='n')


## Plot differences (i.e. where the grammatical vs ungrammatical lines differ in this case for past)
plot_diff(Mod2, view="Time", cond=list(ISPresent=0, ISPresentUngrammatical=0), 
          comp=list(ISUngrammatical=c(0,1)), 
          rm.ranef = TRUE,
          main="Difference in Past grammaticality")
```

```
## Summary:
##  * Time : numeric predictor; with 100 values ranging from -195.000000 to 1195.000000. 
##  * ISPresent : numeric predictor; set to the value(s): 0. 
##  * ISPresentUngrammatical : numeric predictor; set to the value(s): 0. 
##  * Event : factor; set to the value(s): Frank01.Past_gram. (Might be canceled as random effect, check below.) 
##  * NOTE : The following random effects columns are canceled: s(Time,Event)
##
```

```
## 
## Time window(s) of significant difference(s):
##  464.898990 - 1195.000000
```

```
#Plotting for Present
##Plot present grammatical
plot_smooth(Mod2, view="Time", rm.ranef=TRUE, 
            cond=list(ISPresent=1, ISUngrammatical=0,ISPresentUngrammatical=0),
            col=1, xpd=TRUE, eegAxis = TRUE, ylim = c(2, -2), main="Present: C3")
```

```
## Summary:
##  * Time : numeric predictor; with 30 values ranging from -195.000000 to 1195.000000. 
##  * ISPresent : numeric predictor; set to the value(s): 1. 
##  * ISUngrammatical : numeric predictor; set to the value(s): 0. 
##  * ISPresentUngrammatical : numeric predictor; set to the value(s): 0. 
##  * Event : factor; set to the value(s): Frank01.Past_gram. (Might be canceled as random effect, check below.) 
##  * NOTE : The following random effects columns are canceled: s(Time,Event)
##
```

```
##Plot present ungrammatical
plot_smooth(Mod2, view="Time", rm.ranef=TRUE, 
            cond=list(ISPresent=1, ISUngrammatical=1,ISPresentUngrammatical=1),
            add=TRUE, col=2, xpd=TRUE, main = "Present")
```

```
## Summary:
##  * Time : numeric predictor; with 30 values ranging from -195.000000 to 1195.000000. 
##  * ISPresent : numeric predictor; set to the value(s): 1. 
##  * ISUngrammatical : numeric predictor; set to the value(s): 1. 
##  * ISPresentUngrammatical : numeric predictor; set to the value(s): 1. 
##  * Event : factor; set to the value(s): Frank01.Past_gram. (Might be canceled as random effect, check below.) 
##  * NOTE : The following random effects columns are canceled: s(Time,Event)
##
```

```
## Plot differences (i.e. where the grammatical vs ungrammatical lines differ in this case for present)
plot_diff(Mod2, view="Time", cond=list(ISPresent=1), 
          comp=list(ISUngrammatical=c(0,1), ISPresentUngrammatical=c(0,1)), 
          rm.ranef = TRUE,
          main="Difference in Present grammaticality")
```

```
## Summary:
##  * Time : numeric predictor; with 100 values ranging from -195.000000 to 1195.000000. 
##  * ISPresent : numeric predictor; set to the value(s): 1. 
##  * Event : factor; set to the value(s): Frank01.Past_gram. (Might be canceled as random effect, check below.) 
##  * NOTE : The following random effects columns are canceled: s(Time,Event)
##
```

```
## 
## Difference is not significant.
```

#Subsetting data with electrode: Fp1

```
subdat <- droplevels(dat[dat$Channel== "Fp1" & dat$Condition %in% c("Past_gram", "Past_ungram", "Present_gram", "Present_ungram"),]) 
head(subdat)
```

```
##   Subject Condition Grammaticality Channel Time      value Tense ISPresent
## 1 Frank01 Past_gram    Grammatical     Fp1 -195 -1.5544846  Past         0
## 2 Frank01 Past_gram    Grammatical     Fp1 -185  0.4002179  Past         0
## 3 Frank01 Past_gram    Grammatical     Fp1 -175  1.7535096  Past         0
## 4 Frank01 Past_gram    Grammatical     Fp1 -165  1.0395769  Past         0
## 5 Frank01 Past_gram    Grammatical     Fp1 -155 -0.1107179  Past         0
## 6 Frank01 Past_gram    Grammatical     Fp1 -145 -0.4320673  Past         0
##   ISUngrammatical ISPresentUngrammatical
## 1               0                      0
## 2               0                      0
## 3               0                      0
## 4               0                      0
## 5               0                      0
## 6               0                      0
```

## Create EVENT (unique combination of Subject and Items)for random effects

```
subdat$Event <- interaction(subdat$Subject, subdat$Condition)

#   Determine starting point for each time series
subdat <- start_event(subdat,column = "Time",event = "Event")

# Make sure variables are factors
subdat$Condition <- as.factor(subdat$Condition) 
subdat$Subject <- as.factor(subdat$Subject)
```

# GAMM ANALYSIS

```
Mod1 <- bam(value ~ s(Time, k=20) 
            + s(Time, by=ISPresent, k=20)
            + s(Time, by=ISUngrammatical, k=20)
            + s(Time, by=ISPresentUngrammatical, k=20)
            + s(Time,Event, bs='fs', m=1, k=20),
            data=subdat, discrete = TRUE, family="scat")
```

```
## Warning in gam.side(sm, X, tol = .Machine$double.eps^0.5): model has
## repeated 1-d smooths of same variable.
```

```
## Calculate rho value to account for autocorrelation in data.
myrho <- start_value_rho(Mod1)

#Fit a Model including rho
Mod2 <- bam(value ~ s(Time, k=20) 
            + s(Time, by=ISPresent, k=20)
            + s(Time, by=ISUngrammatical, k=20)
            + s(Time, by=ISPresentUngrammatical, k=20)
            + s(Time,Event, bs='fs', m=1, k=20),
            data=subdat, discrete = TRUE, family="scat", 
            AR.start=subdat$start.event, rho=myrho)
```

```
## Warning in gam.side(sm, X, tol = .Machine$double.eps^0.5): model has
## repeated 1-d smooths of same variable.
```

```
#Plot model residuals
qqnorm(resid(Mod2))
qqline(resid(Mod2))
```

#PLOTTING OF GAM model

```
par(mfcol =c(2,2))

#Plotting Past
##Plot past grammatical
plot_smooth(Mod2, view="Time", rm.ranef=TRUE, 
            cond=list(ISPresent=0, ISUngrammatical=0,ISPresentUngrammatical=0),
            col=1, xpd=TRUE, eegAxis = TRUE, ylim = c(2, -2), main="Past: Fp1")
```

```
## Summary:
##  * Time : numeric predictor; with 30 values ranging from -195.000000 to 1195.000000. 
##  * ISPresent : numeric predictor; set to the value(s): 0. 
##  * ISUngrammatical : numeric predictor; set to the value(s): 0. 
##  * ISPresentUngrammatical : numeric predictor; set to the value(s): 0. 
##  * Event : factor; set to the value(s): Frank01.Past_gram. (Might be canceled as random effect, check below.) 
##  * NOTE : The following random effects columns are canceled: s(Time,Event)
##
```

```
##Plot past ungrammatical
plot_smooth(Mod2, view="Time", rm.ranef=TRUE, 
            cond=list(ISPresent=0, ISUngrammatical=1,ISPresentUngrammatical=0),
            h0=0,add=TRUE, v0=0,col=2)
```

```
## Summary:
##  * Time : numeric predictor; with 30 values ranging from -195.000000 to 1195.000000. 
##  * ISPresent : numeric predictor; set to the value(s): 0. 
##  * ISUngrammatical : numeric predictor; set to the value(s): 1. 
##  * ISPresentUngrammatical : numeric predictor; set to the value(s): 0. 
##  * Event : factor; set to the value(s): Frank01.Past_gram. (Might be canceled as random effect, check below.) 
##  * NOTE : The following random effects columns are canceled: s(Time,Event)
##
```

```
legend_margin('bottomleft', legend=c("grammatical", "ungrammatical"),
       fill=c(alpha(1), alpha(2)), border= c(alpha(1), alpha(2)),
         lwd=1.5, col=c(1,2), seg.len=1, merge=TRUE,
         bty='n')


## Plot differences (i.e. where the grammatical vs ungrammatical lines differ in this case for past)
plot_diff(Mod2, view="Time", cond=list(ISPresent=0, ISPresentUngrammatical=0), 
          comp=list(ISUngrammatical=c(0,1)), 
          rm.ranef = TRUE,
          main="Difference in Past grammaticality")
```

```
## Summary:
##  * Time : numeric predictor; with 100 values ranging from -195.000000 to 1195.000000. 
##  * ISPresent : numeric predictor; set to the value(s): 0. 
##  * ISPresentUngrammatical : numeric predictor; set to the value(s): 0. 
##  * Event : factor; set to the value(s): Frank01.Past_gram. (Might be canceled as random effect, check below.) 
##  * NOTE : The following random effects columns are canceled: s(Time,Event)
##
```

```
## 
## Difference is not significant.
```

```
#Plotting for Present
##Plot present grammatical
plot_smooth(Mod2, view="Time", rm.ranef=TRUE, 
            cond=list(ISPresent=1, ISUngrammatical=0,ISPresentUngrammatical=0),
            col=1, xpd=TRUE, eegAxis = TRUE, ylim = c(2, -2), main="Present: Fp1")
```

```
## Summary:
##  * Time : numeric predictor; with 30 values ranging from -195.000000 to 1195.000000. 
##  * ISPresent : numeric predictor; set to the value(s): 1. 
##  * ISUngrammatical : numeric predictor; set to the value(s): 0. 
##  * ISPresentUngrammatical : numeric predictor; set to the value(s): 0. 
##  * Event : factor; set to the value(s): Frank01.Past_gram. (Might be canceled as random effect, check below.) 
##  * NOTE : The following random effects columns are canceled: s(Time,Event)
##
```

```
##Plot present ungrammatical
plot_smooth(Mod2, view="Time", rm.ranef=TRUE, 
            cond=list(ISPresent=1, ISUngrammatical=1,ISPresentUngrammatical=1),
            add=TRUE, col=2, xpd=TRUE, main = "Present")
```

```
## Summary:
##  * Time : numeric predictor; with 30 values ranging from -195.000000 to 1195.000000. 
##  * ISPresent : numeric predictor; set to the value(s): 1. 
##  * ISUngrammatical : numeric predictor; set to the value(s): 1. 
##  * ISPresentUngrammatical : numeric predictor; set to the value(s): 1. 
##  * Event : factor; set to the value(s): Frank01.Past_gram. (Might be canceled as random effect, check below.) 
##  * NOTE : The following random effects columns are canceled: s(Time,Event)
##
```

```
## Plot differences (i.e. where the grammatical vs ungrammatical lines differ in this case for present)
plot_diff(Mod2, view="Time", cond=list(ISPresent=1), 
          comp=list(ISUngrammatical=c(0,1), ISPresentUngrammatical=c(0,1)), 
          rm.ranef = TRUE,
          main="Difference in Present grammaticality")
```

```
## Summary:
##  * Time : numeric predictor; with 100 values ranging from -195.000000 to 1195.000000. 
##  * ISPresent : numeric predictor; set to the value(s): 1. 
##  * Event : factor; set to the value(s): Frank01.Past_gram. (Might be canceled as random effect, check below.) 
##  * NOTE : The following random effects columns are canceled: s(Time,Event)
##
```

```
## 
## Difference is not significant.
```

#Subsetting data with electrode: Fpz

```
subdat <- droplevels(dat[dat$Channel== "Fpz" & dat$Condition %in% c("Past_gram", "Past_ungram", "Present_gram", "Present_ungram"),]) 
head(subdat)
```

```
##     Subject Condition Grammaticality Channel Time      value Tense
## 141 Frank01 Past_gram    Grammatical     Fpz -195 -1.3970462  Past
## 142 Frank01 Past_gram    Grammatical     Fpz -185  0.8209231  Past
## 143 Frank01 Past_gram    Grammatical     Fpz -175  2.2695865  Past
## 144 Frank01 Past_gram    Grammatical     Fpz -165  1.1561462  Past
## 145 Frank01 Past_gram    Grammatical     Fpz -155 -0.1334487  Past
## 146 Frank01 Past_gram    Grammatical     Fpz -145 -0.3763365  Past
##     ISPresent ISUngrammatical ISPresentUngrammatical
## 141         0               0                      0
## 142         0               0                      0
## 143         0               0                      0
## 144         0               0                      0
## 145         0               0                      0
## 146         0               0                      0
```

## Create EVENT (unique combination of Subject and Items)for random effects

```
subdat$Event <- interaction(subdat$Subject, subdat$Condition)

#   Determine starting point for each time series
subdat <- start_event(subdat,column = "Time",event = "Event")

# Make sure variables are factors
subdat$Condition <- as.factor(subdat$Condition) 
subdat$Subject <- as.factor(subdat$Subject)
```

# GAMM ANALYSIS

```
Mod1 <- bam(value ~ s(Time, k=20) 
            + s(Time, by=ISPresent, k=20)
            + s(Time, by=ISUngrammatical, k=20)
            + s(Time, by=ISPresentUngrammatical, k=20)
            + s(Time,Event, bs='fs', m=1, k=20),
            data=subdat, discrete = TRUE, family="scat")
```

```
## Warning in gam.side(sm, X, tol = .Machine$double.eps^0.5): model has
## repeated 1-d smooths of same variable.
```

```
## Calculate rho value to account for autocorrelation in data.
myrho <- start_value_rho(Mod1)

#Fit a Model including rho
Mod2 <- bam(value ~ s(Time, k=20) 
            + s(Time, by=ISPresent, k=20)
            + s(Time, by=ISUngrammatical, k=20)
            + s(Time, by=ISPresentUngrammatical, k=20)
            + s(Time,Event, bs='fs', m=1, k=20),
            data=subdat, discrete = TRUE, family="scat", 
            AR.start=subdat$start.event, rho=myrho)
```

```
## Warning in gam.side(sm, X, tol = .Machine$double.eps^0.5): model has
## repeated 1-d smooths of same variable.
```

```
#Plot model residuals
qqnorm(resid(Mod2))
qqline(resid(Mod2))
```

#PLOTTING OF GAM model

```
par(mfcol =c(2,2))

#Plotting Past
##Plot past grammatical
plot_smooth(Mod2, view="Time", rm.ranef=TRUE, 
            cond=list(ISPresent=0, ISUngrammatical=0,ISPresentUngrammatical=0),
            col=1, xpd=TRUE, eegAxis = TRUE, ylim = c(2, -2), main="Past: Fpz")
```

```
## Summary:
##  * Time : numeric predictor; with 30 values ranging from -195.000000 to 1195.000000. 
##  * ISPresent : numeric predictor; set to the value(s): 0. 
##  * ISUngrammatical : numeric predictor; set to the value(s): 0. 
##  * ISPresentUngrammatical : numeric predictor; set to the value(s): 0. 
##  * Event : factor; set to the value(s): Frank01.Past_gram. (Might be canceled as random effect, check below.) 
##  * NOTE : The following random effects columns are canceled: s(Time,Event)
##
```

```
##Plot past ungrammatical
plot_smooth(Mod2, view="Time", rm.ranef=TRUE, 
            cond=list(ISPresent=0, ISUngrammatical=1,ISPresentUngrammatical=0),
            h0=0,add=TRUE, v0=0,col=2)
```

```
## Summary:
##  * Time : numeric predictor; with 30 values ranging from -195.000000 to 1195.000000. 
##  * ISPresent : numeric predictor; set to the value(s): 0. 
##  * ISUngrammatical : numeric predictor; set to the value(s): 1. 
##  * ISPresentUngrammatical : numeric predictor; set to the value(s): 0. 
##  * Event : factor; set to the value(s): Frank01.Past_gram. (Might be canceled as random effect, check below.) 
##  * NOTE : The following random effects columns are canceled: s(Time,Event)
##
```

```
legend_margin('bottomleft', legend=c("grammatical", "ungrammatical"),
       fill=c(alpha(1), alpha(2)), border= c(alpha(1), alpha(2)),
         lwd=1.5, col=c(1,2), seg.len=1, merge=TRUE,
         bty='n')


## Plot differences (i.e. where the grammatical vs ungrammatical lines differ in this case for past)
plot_diff(Mod2, view="Time", cond=list(ISPresent=0, ISPresentUngrammatical=0), 
          comp=list(ISUngrammatical=c(0,1)), 
          rm.ranef = TRUE,
          main="Difference in Past grammaticality")
```

```
## Summary:
##  * Time : numeric predictor; with 100 values ranging from -195.000000 to 1195.000000. 
##  * ISPresent : numeric predictor; set to the value(s): 0. 
##  * ISPresentUngrammatical : numeric predictor; set to the value(s): 0. 
##  * Event : factor; set to the value(s): Frank01.Past_gram. (Might be canceled as random effect, check below.) 
##  * NOTE : The following random effects columns are canceled: s(Time,Event)
##
```

```
## 
## Difference is not significant.
```

```
#Plotting for Present
##Plot present grammatical
plot_smooth(Mod2, view="Time", rm.ranef=TRUE, 
            cond=list(ISPresent=1, ISUngrammatical=0,ISPresentUngrammatical=0),
            col=1, xpd=TRUE, eegAxis = TRUE, ylim = c(2, -2), main="Present: Fpz")
```

```
## Summary:
##  * Time : numeric predictor; with 30 values ranging from -195.000000 to 1195.000000. 
##  * ISPresent : numeric predictor; set to the value(s): 1. 
##  * ISUngrammatical : numeric predictor; set to the value(s): 0. 
##  * ISPresentUngrammatical : numeric predictor; set to the value(s): 0. 
##  * Event : factor; set to the value(s): Frank01.Past_gram. (Might be canceled as random effect, check below.) 
##  * NOTE : The following random effects columns are canceled: s(Time,Event)
##
```

```
##Plot present ungrammatical
plot_smooth(Mod2, view="Time", rm.ranef=TRUE, 
            cond=list(ISPresent=1, ISUngrammatical=1,ISPresentUngrammatical=1),
            add=TRUE, col=2, xpd=TRUE, main = "Present")
```

```
## Summary:
##  * Time : numeric predictor; with 30 values ranging from -195.000000 to 1195.000000. 
##  * ISPresent : numeric predictor; set to the value(s): 1. 
##  * ISUngrammatical : numeric predictor; set to the value(s): 1. 
##  * ISPresentUngrammatical : numeric predictor; set to the value(s): 1. 
##  * Event : factor; set to the value(s): Frank01.Past_gram. (Might be canceled as random effect, check below.) 
##  * NOTE : The following random effects columns are canceled: s(Time,Event)
##
```

```
## Plot differences (i.e. where the grammatical vs ungrammatical lines differ in this case for present)
plot_diff(Mod2, view="Time", cond=list(ISPresent=1), 
          comp=list(ISUngrammatical=c(0,1), ISPresentUngrammatical=c(0,1)), 
          rm.ranef = TRUE,
          main="Difference in Present grammaticality")
```

```
## Summary:
##  * Time : numeric predictor; with 100 values ranging from -195.000000 to 1195.000000. 
##  * ISPresent : numeric predictor; set to the value(s): 1. 
##  * Event : factor; set to the value(s): Frank01.Past_gram. (Might be canceled as random effect, check below.) 
##  * NOTE : The following random effects columns are canceled: s(Time,Event)
##
```

```
## 
## Difference is not significant.
```

#Subsetting data with electrode: Fp2

```
subdat <- droplevels(dat[dat$Channel== "Fp2" & dat$Condition %in% c("Past_gram", "Past_ungram", "Present_gram", "Present_ungram"),]) 
head(subdat)
```

```
##     Subject Condition Grammaticality Channel Time      value Tense
## 281 Frank01 Past_gram    Grammatical     Fp2 -195 -1.1255308  Past
## 282 Frank01 Past_gram    Grammatical     Fp2 -185  1.1452885  Past
## 283 Frank01 Past_gram    Grammatical     Fp2 -175  2.7015096  Past
## 284 Frank01 Past_gram    Grammatical     Fp2 -165  2.1040692  Past
## 285 Frank01 Past_gram    Grammatical     Fp2 -155  0.7362051  Past
## 286 Frank01 Past_gram    Grammatical     Fp2 -145 -0.2749038  Past
##     ISPresent ISUngrammatical ISPresentUngrammatical
## 281         0               0                      0
## 282         0               0                      0
## 283         0               0                      0
## 284         0               0                      0
## 285         0               0                      0
## 286         0               0                      0
```

## Create EVENT (unique combination of Subject and Items)for random effects

```
subdat$Event <- interaction(subdat$Subject, subdat$Condition)

#   Determine starting point for each time series
subdat <- start_event(subdat,column = "Time",event = "Event")

# Make sure variables are factors
subdat$Condition <- as.factor(subdat$Condition) 
subdat$Subject <- as.factor(subdat$Subject)
```

# GAMM ANALYSIS

```
Mod1 <- bam(value ~ s(Time, k=20) 
            + s(Time, by=ISPresent, k=20)
            + s(Time, by=ISUngrammatical, k=20)
            + s(Time, by=ISPresentUngrammatical, k=20)
            + s(Time,Event, bs='fs', m=1, k=20),
            data=subdat, discrete = TRUE, family="scat")
```

```
## Warning in gam.side(sm, X, tol = .Machine$double.eps^0.5): model has
## repeated 1-d smooths of same variable.
```

```
## Calculate rho value to account for autocorrelation in data.
myrho <- start_value_rho(Mod1)

#Fit a Model including rho
Mod2 <- bam(value ~ s(Time, k=20) 
            + s(Time, by=ISPresent, k=20)
            + s(Time, by=ISUngrammatical, k=20)
            + s(Time, by=ISPresentUngrammatical, k=20)
            + s(Time,Event, bs='fs', m=1, k=20),
            data=subdat, discrete = TRUE, family="scat", 
            AR.start=subdat$start.event, rho=myrho)
```

```
## Warning in gam.side(sm, X, tol = .Machine$double.eps^0.5): model has
## repeated 1-d smooths of same variable.
```

```
#Plot model residuals
qqnorm(resid(Mod2))
qqline(resid(Mod2))
```

#PLOTTING OF GAM model

```
par(mfcol =c(2,2))

#Plotting Past
##Plot past grammatical
plot_smooth(Mod2, view="Time", rm.ranef=TRUE, 
            cond=list(ISPresent=0, ISUngrammatical=0,ISPresentUngrammatical=0),
            col=1, xpd=TRUE, eegAxis = TRUE, ylim = c(2, -2), main="Past: Fp2")
```

```
## Summary:
##  * Time : numeric predictor; with 30 values ranging from -195.000000 to 1195.000000. 
##  * ISPresent : numeric predictor; set to the value(s): 0. 
##  * ISUngrammatical : numeric predictor; set to the value(s): 0. 
##  * ISPresentUngrammatical : numeric predictor; set to the value(s): 0. 
##  * Event : factor; set to the value(s): Frank01.Past_gram. (Might be canceled as random effect, check below.) 
##  * NOTE : The following random effects columns are canceled: s(Time,Event)
##
```

```
##Plot past ungrammatical
plot_smooth(Mod2, view="Time", rm.ranef=TRUE, 
            cond=list(ISPresent=0, ISUngrammatical=1,ISPresentUngrammatical=0),
            h0=0,add=TRUE, v0=0,col=2)
```

```
## Summary:
##  * Time : numeric predictor; with 30 values ranging from -195.000000 to 1195.000000. 
##  * ISPresent : numeric predictor; set to the value(s): 0. 
##  * ISUngrammatical : numeric predictor; set to the value(s): 1. 
##  * ISPresentUngrammatical : numeric predictor; set to the value(s): 0. 
##  * Event : factor; set to the value(s): Frank01.Past_gram. (Might be canceled as random effect, check below.) 
##  * NOTE : The following random effects columns are canceled: s(Time,Event)
##
```

```
legend_margin('bottomleft', legend=c("grammatical", "ungrammatical"),
       fill=c(alpha(1), alpha(2)), border= c(alpha(1), alpha(2)),
         lwd=1.5, col=c(1,2), seg.len=1, merge=TRUE,
         bty='n')


## Plot differences (i.e. where the grammatical vs ungrammatical lines differ in this case for past)
plot_diff(Mod2, view="Time", cond=list(ISPresent=0, ISPresentUngrammatical=0), 
          comp=list(ISUngrammatical=c(0,1)), 
          rm.ranef = TRUE,
          main="Difference in Past grammaticality")
```

```
## Summary:
##  * Time : numeric predictor; with 100 values ranging from -195.000000 to 1195.000000. 
##  * ISPresent : numeric predictor; set to the value(s): 0. 
##  * ISPresentUngrammatical : numeric predictor; set to the value(s): 0. 
##  * Event : factor; set to the value(s): Frank01.Past_gram. (Might be canceled as random effect, check below.) 
##  * NOTE : The following random effects columns are canceled: s(Time,Event)
##
```

```
## 
## Difference is not significant.
```

```
#Plotting for Present
##Plot present grammatical
plot_smooth(Mod2, view="Time", rm.ranef=TRUE, 
            cond=list(ISPresent=1, ISUngrammatical=0,ISPresentUngrammatical=0),
            col=1, xpd=TRUE, eegAxis = TRUE, ylim = c(2, -2), main="Present: Fp2")
```

```
## Summary:
##  * Time : numeric predictor; with 30 values ranging from -195.000000 to 1195.000000. 
##  * ISPresent : numeric predictor; set to the value(s): 1. 
##  * ISUngrammatical : numeric predictor; set to the value(s): 0. 
##  * ISPresentUngrammatical : numeric predictor; set to the value(s): 0. 
##  * Event : factor; set to the value(s): Frank01.Past_gram. (Might be canceled as random effect, check below.) 
##  * NOTE : The following random effects columns are canceled: s(Time,Event)
##
```

```
##Plot present ungrammatical
plot_smooth(Mod2, view="Time", rm.ranef=TRUE, 
            cond=list(ISPresent=1, ISUngrammatical=1,ISPresentUngrammatical=1),
            add=TRUE, col=2, xpd=TRUE, main = "Present")
```

```
## Summary:
##  * Time : numeric predictor; with 30 values ranging from -195.000000 to 1195.000000. 
##  * ISPresent : numeric predictor; set to the value(s): 1. 
##  * ISUngrammatical : numeric predictor; set to the value(s): 1. 
##  * ISPresentUngrammatical : numeric predictor; set to the value(s): 1. 
##  * Event : factor; set to the value(s): Frank01.Past_gram. (Might be canceled as random effect, check below.) 
##  * NOTE : The following random effects columns are canceled: s(Time,Event)
##
```

```
## Plot differences (i.e. where the grammatical vs ungrammatical lines differ in this case for present)
plot_diff(Mod2, view="Time", cond=list(ISPresent=1), 
          comp=list(ISUngrammatical=c(0,1), ISPresentUngrammatical=c(0,1)), 
          rm.ranef = TRUE,
          main="Difference in Present grammaticality")
```

```
## Summary:
##  * Time : numeric predictor; with 100 values ranging from -195.000000 to 1195.000000. 
##  * ISPresent : numeric predictor; set to the value(s): 1. 
##  * Event : factor; set to the value(s): Frank01.Past_gram. (Might be canceled as random effect, check below.) 
##  * NOTE : The following random effects columns are canceled: s(Time,Event)
##
```

```
## 
## Difference is not significant.
```

#Subsetting data with electrode: F7

```
subdat <- droplevels(dat[dat$Channel== "F7" & dat$Condition %in% c("Past_gram", "Past_ungram", "Present_gram", "Present_ungram"),]) 
head(subdat)
```

```
##     Subject Condition Grammaticality Channel Time        value Tense
## 421 Frank01 Past_gram    Grammatical      F7 -195 -0.422138462  Past
## 422 Frank01 Past_gram    Grammatical      F7 -185  0.580262821  Past
## 423 Frank01 Past_gram    Grammatical      F7 -175  0.718000000  Past
## 424 Frank01 Past_gram    Grammatical      F7 -165 -0.056915385  Past
## 425 Frank01 Past_gram    Grammatical      F7 -155 -0.367147436  Past
## 426 Frank01 Past_gram    Grammatical      F7 -145  0.009528846  Past
##     ISPresent ISUngrammatical ISPresentUngrammatical
## 421         0               0                      0
## 422         0               0                      0
## 423         0               0                      0
## 424         0               0                      0
## 425         0               0                      0
## 426         0               0                      0
```

## Create EVENT (unique combination of Subject and Items)for random effects

```
subdat$Event <- interaction(subdat$Subject, subdat$Condition)

#   Determine starting point for each time series
subdat <- start_event(subdat,column = "Time",event = "Event")

# Make sure variables are factors
subdat$Condition <- as.factor(subdat$Condition) 
subdat$Subject <- as.factor(subdat$Subject)
```

# GAMM ANALYSIS

```
Mod1 <- bam(value ~ s(Time, k=20) 
            + s(Time, by=ISPresent, k=20)
            + s(Time, by=ISUngrammatical, k=20)
            + s(Time, by=ISPresentUngrammatical, k=20)
            + s(Time,Event, bs='fs', m=1, k=20),
            data=subdat, discrete = TRUE, family="scat")
```

```
## Warning in gam.side(sm, X, tol = .Machine$double.eps^0.5): model has
## repeated 1-d smooths of same variable.
```

```
## Calculate rho value to account for autocorrelation in data.
myrho <- start_value_rho(Mod1)

#Fit a Model including rho
Mod2 <- bam(value ~ s(Time, k=20) 
            + s(Time, by=ISPresent, k=20)
            + s(Time, by=ISUngrammatical, k=20)
            + s(Time, by=ISPresentUngrammatical, k=20)
            + s(Time,Event, bs='fs', m=1, k=20),
            data=subdat, discrete = TRUE, family="scat", 
            AR.start=subdat$start.event, rho=myrho)
```

```
## Warning in gam.side(sm, X, tol = .Machine$double.eps^0.5): model has
## repeated 1-d smooths of same variable.
```

```
#Plot model residuals
qqnorm(resid(Mod2))
qqline(resid(Mod2))
```

#PLOTTING OF GAM model

```
par(mfcol =c(2,2))

#Plotting Past
##Plot past grammatical
plot_smooth(Mod2, view="Time", rm.ranef=TRUE, 
            cond=list(ISPresent=0, ISUngrammatical=0,ISPresentUngrammatical=0),
            col=1, xpd=TRUE, eegAxis = TRUE, ylim = c(2, -2), main="Past: F7")
```

```
## Summary:
##  * Time : numeric predictor; with 30 values ranging from -195.000000 to 1195.000000. 
##  * ISPresent : numeric predictor; set to the value(s): 0. 
##  * ISUngrammatical : numeric predictor; set to the value(s): 0. 
##  * ISPresentUngrammatical : numeric predictor; set to the value(s): 0. 
##  * Event : factor; set to the value(s): Frank01.Past_gram. (Might be canceled as random effect, check below.) 
##  * NOTE : The following random effects columns are canceled: s(Time,Event)
##
```

```
##Plot past ungrammatical
plot_smooth(Mod2, view="Time", rm.ranef=TRUE, 
            cond=list(ISPresent=0, ISUngrammatical=1,ISPresentUngrammatical=0),
            h0=0,add=TRUE, v0=0,col=2)
```

```
## Summary:
##  * Time : numeric predictor; with 30 values ranging from -195.000000 to 1195.000000. 
##  * ISPresent : numeric predictor; set to the value(s): 0. 
##  * ISUngrammatical : numeric predictor; set to the value(s): 1. 
##  * ISPresentUngrammatical : numeric predictor; set to the value(s): 0. 
##  * Event : factor; set to the value(s): Frank01.Past_gram. (Might be canceled as random effect, check below.) 
##  * NOTE : The following random effects columns are canceled: s(Time,Event)
##
```

```
legend_margin('bottomleft', legend=c("grammatical", "ungrammatical"),
       fill=c(alpha(1), alpha(2)), border= c(alpha(1), alpha(2)),
         lwd=1.5, col=c(1,2), seg.len=1, merge=TRUE,
         bty='n')


## Plot differences (i.e. where the grammatical vs ungrammatical lines differ in this case for past)
plot_diff(Mod2, view="Time", cond=list(ISPresent=0, ISPresentUngrammatical=0), 
          comp=list(ISUngrammatical=c(0,1)), 
          rm.ranef = TRUE,
          main="Difference in Past grammaticality")
```

```
## Summary:
##  * Time : numeric predictor; with 100 values ranging from -195.000000 to 1195.000000. 
##  * ISPresent : numeric predictor; set to the value(s): 0. 
##  * ISPresentUngrammatical : numeric predictor; set to the value(s): 0. 
##  * Event : factor; set to the value(s): Frank01.Past_gram. (Might be canceled as random effect, check below.) 
##  * NOTE : The following random effects columns are canceled: s(Time,Event)
##
```

```
## 
## Difference is not significant.
```

```
#Plotting for Present
##Plot present grammatical
plot_smooth(Mod2, view="Time", rm.ranef=TRUE, 
            cond=list(ISPresent=1, ISUngrammatical=0,ISPresentUngrammatical=0),
            col=1, xpd=TRUE, eegAxis = TRUE, ylim = c(2, -2), main="Present: F7")
```

```
## Summary:
##  * Time : numeric predictor; with 30 values ranging from -195.000000 to 1195.000000. 
##  * ISPresent : numeric predictor; set to the value(s): 1. 
##  * ISUngrammatical : numeric predictor; set to the value(s): 0. 
##  * ISPresentUngrammatical : numeric predictor; set to the value(s): 0. 
##  * Event : factor; set to the value(s): Frank01.Past_gram. (Might be canceled as random effect, check below.) 
##  * NOTE : The following random effects columns are canceled: s(Time,Event)
##
```

```
##Plot present ungrammatical
plot_smooth(Mod2, view="Time", rm.ranef=TRUE, 
            cond=list(ISPresent=1, ISUngrammatical=1,ISPresentUngrammatical=1),
            add=TRUE, col=2, xpd=TRUE, main = "Present")
```

```
## Summary:
##  * Time : numeric predictor; with 30 values ranging from -195.000000 to 1195.000000. 
##  * ISPresent : numeric predictor; set to the value(s): 1. 
##  * ISUngrammatical : numeric predictor; set to the value(s): 1. 
##  * ISPresentUngrammatical : numeric predictor; set to the value(s): 1. 
##  * Event : factor; set to the value(s): Frank01.Past_gram. (Might be canceled as random effect, check below.) 
##  * NOTE : The following random effects columns are canceled: s(Time,Event)
##
```

```
## Plot differences (i.e. where the grammatical vs ungrammatical lines differ in this case for present)
plot_diff(Mod2, view="Time", cond=list(ISPresent=1), 
          comp=list(ISUngrammatical=c(0,1), ISPresentUngrammatical=c(0,1)), 
          rm.ranef = TRUE,
          main="Difference in Present grammaticality")
```

```
## Summary:
##  * Time : numeric predictor; with 100 values ranging from -195.000000 to 1195.000000. 
##  * ISPresent : numeric predictor; set to the value(s): 1. 
##  * Event : factor; set to the value(s): Frank01.Past_gram. (Might be canceled as random effect, check below.) 
##  * NOTE : The following random effects columns are canceled: s(Time,Event)
##
```

```
## 
## Difference is not significant.
```

#Subsetting data with electrode: F3

```
subdat <- droplevels(dat[dat$Channel== "F3" & dat$Condition %in% c("Past_gram", "Past_ungram", "Present_gram", "Present_ungram"),]) 
head(subdat)
```

```
##     Subject Condition Grammaticality Channel Time      value Tense
## 561 Frank01 Past_gram    Grammatical      F3 -195 -0.6265231  Past
## 562 Frank01 Past_gram    Grammatical      F3 -185  0.4853141  Past
## 563 Frank01 Past_gram    Grammatical      F3 -175  1.3311346  Past
## 564 Frank01 Past_gram    Grammatical      F3 -165  0.6907692  Past
## 565 Frank01 Past_gram    Grammatical      F3 -155 -0.3948333  Past
## 566 Frank01 Past_gram    Grammatical      F3 -145 -0.8902788  Past
##     ISPresent ISUngrammatical ISPresentUngrammatical
## 561         0               0                      0
## 562         0               0                      0
## 563         0               0                      0
## 564         0               0                      0
## 565         0               0                      0
## 566         0               0                      0
```

## Create EVENT (unique combination of Subject and Items)for random effects

```
subdat$Event <- interaction(subdat$Subject, subdat$Condition)

#   Determine starting point for each time series
subdat <- start_event(subdat,column = "Time",event = "Event")

# Make sure variables are factors
subdat$Condition <- as.factor(subdat$Condition) 
subdat$Subject <- as.factor(subdat$Subject)
```

# GAMM ANALYSIS

```
Mod1 <- bam(value ~ s(Time, k=20) 
            + s(Time, by=ISPresent, k=20)
            + s(Time, by=ISUngrammatical, k=20)
            + s(Time, by=ISPresentUngrammatical, k=20)
            + s(Time,Event, bs='fs', m=1, k=20),
            data=subdat, discrete = TRUE, family="scat")
```

```
## Warning in gam.side(sm, X, tol = .Machine$double.eps^0.5): model has
## repeated 1-d smooths of same variable.
```

```
## Calculate rho value to account for autocorrelation in data.
myrho <- start_value_rho(Mod1)

#Fit a Model including rho
Mod2 <- bam(value ~ s(Time, k=20) 
            + s(Time, by=ISPresent, k=20)
            + s(Time, by=ISUngrammatical, k=20)
            + s(Time, by=ISPresentUngrammatical, k=20)
            + s(Time,Event, bs='fs', m=1, k=20),
            data=subdat, discrete = TRUE, family="scat", 
            AR.start=subdat$start.event, rho=myrho)
```

```
## Warning in gam.side(sm, X, tol = .Machine$double.eps^0.5): model has
## repeated 1-d smooths of same variable.
```

```
#Plot model residuals
qqnorm(resid(Mod2))
qqline(resid(Mod2))
```

#PLOTTING OF GAM model

```
par(mfcol =c(2,2))

#Plotting Past
##Plot past grammatical
plot_smooth(Mod2, view="Time", rm.ranef=TRUE, 
            cond=list(ISPresent=0, ISUngrammatical=0,ISPresentUngrammatical=0),
            col=1, xpd=TRUE, eegAxis = TRUE, ylim = c(2, -2), main="Past: F3")
```

```
## Summary:
##  * Time : numeric predictor; with 30 values ranging from -195.000000 to 1195.000000. 
##  * ISPresent : numeric predictor; set to the value(s): 0. 
##  * ISUngrammatical : numeric predictor; set to the value(s): 0. 
##  * ISPresentUngrammatical : numeric predictor; set to the value(s): 0. 
##  * Event : factor; set to the value(s): Frank01.Past_gram. (Might be canceled as random effect, check below.) 
##  * NOTE : The following random effects columns are canceled: s(Time,Event)
##
```

```
##Plot past ungrammatical
plot_smooth(Mod2, view="Time", rm.ranef=TRUE, 
            cond=list(ISPresent=0, ISUngrammatical=1,ISPresentUngrammatical=0),
            h0=0,add=TRUE, v0=0,col=2)
```

```
## Summary:
##  * Time : numeric predictor; with 30 values ranging from -195.000000 to 1195.000000. 
##  * ISPresent : numeric predictor; set to the value(s): 0. 
##  * ISUngrammatical : numeric predictor; set to the value(s): 1. 
##  * ISPresentUngrammatical : numeric predictor; set to the value(s): 0. 
##  * Event : factor; set to the value(s): Frank01.Past_gram. (Might be canceled as random effect, check below.) 
##  * NOTE : The following random effects columns are canceled: s(Time,Event)
##
```

```
legend_margin('bottomleft', legend=c("grammatical", "ungrammatical"),
       fill=c(alpha(1), alpha(2)), border= c(alpha(1), alpha(2)),
         lwd=1.5, col=c(1,2), seg.len=1, merge=TRUE,
         bty='n')


## Plot differences (i.e. where the grammatical vs ungrammatical lines differ in this case for past)
plot_diff(Mod2, view="Time", cond=list(ISPresent=0, ISPresentUngrammatical=0), 
          comp=list(ISUngrammatical=c(0,1)), 
          rm.ranef = TRUE,
          main="Difference in Past grammaticality")
```

```
## Summary:
##  * Time : numeric predictor; with 100 values ranging from -195.000000 to 1195.000000. 
##  * ISPresent : numeric predictor; set to the value(s): 0. 
##  * ISPresentUngrammatical : numeric predictor; set to the value(s): 0. 
##  * Event : factor; set to the value(s): Frank01.Past_gram. (Might be canceled as random effect, check below.) 
##  * NOTE : The following random effects columns are canceled: s(Time,Event)
##
```

```
## 
## Difference is not significant.
```

```
#Plotting for Present
##Plot present grammatical
plot_smooth(Mod2, view="Time", rm.ranef=TRUE, 
            cond=list(ISPresent=1, ISUngrammatical=0,ISPresentUngrammatical=0),
            col=1, xpd=TRUE, eegAxis = TRUE, ylim = c(2, -2), main="Present: F3")
```

```
## Summary:
##  * Time : numeric predictor; with 30 values ranging from -195.000000 to 1195.000000. 
##  * ISPresent : numeric predictor; set to the value(s): 1. 
##  * ISUngrammatical : numeric predictor; set to the value(s): 0. 
##  * ISPresentUngrammatical : numeric predictor; set to the value(s): 0. 
##  * Event : factor; set to the value(s): Frank01.Past_gram. (Might be canceled as random effect, check below.) 
##  * NOTE : The following random effects columns are canceled: s(Time,Event)
##
```

```
##Plot present ungrammatical
plot_smooth(Mod2, view="Time", rm.ranef=TRUE, 
            cond=list(ISPresent=1, ISUngrammatical=1,ISPresentUngrammatical=1),
            add=TRUE, col=2, xpd=TRUE, main = "Present")
```

```
## Summary:
##  * Time : numeric predictor; with 30 values ranging from -195.000000 to 1195.000000. 
##  * ISPresent : numeric predictor; set to the value(s): 1. 
##  * ISUngrammatical : numeric predictor; set to the value(s): 1. 
##  * ISPresentUngrammatical : numeric predictor; set to the value(s): 1. 
##  * Event : factor; set to the value(s): Frank01.Past_gram. (Might be canceled as random effect, check below.) 
##  * NOTE : The following random effects columns are canceled: s(Time,Event)
##
```

```
## Plot differences (i.e. where the grammatical vs ungrammatical lines differ in this case for present)
plot_diff(Mod2, view="Time", cond=list(ISPresent=1), 
          comp=list(ISUngrammatical=c(0,1), ISPresentUngrammatical=c(0,1)), 
          rm.ranef = TRUE,
          main="Difference in Present grammaticality")
```

```
## Summary:
##  * Time : numeric predictor; with 100 values ranging from -195.000000 to 1195.000000. 
##  * ISPresent : numeric predictor; set to the value(s): 1. 
##  * Event : factor; set to the value(s): Frank01.Past_gram. (Might be canceled as random effect, check below.) 
##  * NOTE : The following random effects columns are canceled: s(Time,Event)
##
```

```
## 
## Difference is not significant.
```

#Subsetting data with electrode: F4

```
subdat <- droplevels(dat[dat$Channel== "F4" & dat$Condition %in% c("Past_gram", "Past_ungram", "Present_gram", "Present_ungram"),]) 
head(subdat)
```

```
##     Subject Condition Grammaticality Channel Time      value Tense
## 841 Frank01 Past_gram    Grammatical      F4 -195 -0.8289308  Past
## 842 Frank01 Past_gram    Grammatical      F4 -185  0.6650128  Past
## 843 Frank01 Past_gram    Grammatical      F4 -175  1.8301635  Past
## 844 Frank01 Past_gram    Grammatical      F4 -165  1.0202385  Past
## 845 Frank01 Past_gram    Grammatical      F4 -155 -0.3058333  Past
## 846 Frank01 Past_gram    Grammatical      F4 -145 -1.2457212  Past
##     ISPresent ISUngrammatical ISPresentUngrammatical
## 841         0               0                      0
## 842         0               0                      0
## 843         0               0                      0
## 844         0               0                      0
## 845         0               0                      0
## 846         0               0                      0
```

## Create EVENT (unique combination of Subject and Items)for random effects

```
subdat$Event <- interaction(subdat$Subject, subdat$Condition)

#   Determine starting point for each time series
subdat <- start_event(subdat,column = "Time",event = "Event")

# Make sure variables are factors
subdat$Condition <- as.factor(subdat$Condition) 
subdat$Subject <- as.factor(subdat$Subject)
```

# GAMM ANALYSIS

```
Mod1 <- bam(value ~ s(Time, k=20) 
            + s(Time, by=ISPresent, k=20)
            + s(Time, by=ISUngrammatical, k=20)
            + s(Time, by=ISPresentUngrammatical, k=20)
            + s(Time,Event, bs='fs', m=1, k=20),
            data=subdat, discrete = TRUE, family="scat")
```

```
## Warning in gam.side(sm, X, tol = .Machine$double.eps^0.5): model has
## repeated 1-d smooths of same variable.
```

```
## Calculate rho value to account for autocorrelation in data.
myrho <- start_value_rho(Mod1)

#Fit a Model including rho
Mod2 <- bam(value ~ s(Time, k=20) 
            + s(Time, by=ISPresent, k=20)
            + s(Time, by=ISUngrammatical, k=20)
            + s(Time, by=ISPresentUngrammatical, k=20)
            + s(Time,Event, bs='fs', m=1, k=20),
            data=subdat, discrete = TRUE, family="scat", 
            AR.start=subdat$start.event, rho=myrho)
```

```
## Warning in gam.side(sm, X, tol = .Machine$double.eps^0.5): model has
## repeated 1-d smooths of same variable.
```

```
#Plot model residuals
qqnorm(resid(Mod2))
qqline(resid(Mod2))
```

#PLOTTING OF GAM model

```
par(mfcol =c(2,2))

#Plotting Past
##Plot past grammatical
plot_smooth(Mod2, view="Time", rm.ranef=TRUE, 
            cond=list(ISPresent=0, ISUngrammatical=0,ISPresentUngrammatical=0),
            col=1, xpd=TRUE, eegAxis = TRUE, ylim = c(2, -2), main="Past: F4")
```

```
## Summary:
##  * Time : numeric predictor; with 30 values ranging from -195.000000 to 1195.000000. 
##  * ISPresent : numeric predictor; set to the value(s): 0. 
##  * ISUngrammatical : numeric predictor; set to the value(s): 0. 
##  * ISPresentUngrammatical : numeric predictor; set to the value(s): 0. 
##  * Event : factor; set to the value(s): Frank01.Past_gram. (Might be canceled as random effect, check below.) 
##  * NOTE : The following random effects columns are canceled: s(Time,Event)
##
```

```
##Plot past ungrammatical
plot_smooth(Mod2, view="Time", rm.ranef=TRUE, 
            cond=list(ISPresent=0, ISUngrammatical=1,ISPresentUngrammatical=0),
            h0=0,add=TRUE, v0=0,col=2)
```

```
## Summary:
##  * Time : numeric predictor; with 30 values ranging from -195.000000 to 1195.000000. 
##  * ISPresent : numeric predictor; set to the value(s): 0. 
##  * ISUngrammatical : numeric predictor; set to the value(s): 1. 
##  * ISPresentUngrammatical : numeric predictor; set to the value(s): 0. 
##  * Event : factor; set to the value(s): Frank01.Past_gram. (Might be canceled as random effect, check below.) 
##  * NOTE : The following random effects columns are canceled: s(Time,Event)
##
```

```
legend_margin('bottomleft', legend=c("grammatical", "ungrammatical"),
       fill=c(alpha(1), alpha(2)), border= c(alpha(1), alpha(2)),
         lwd=1.5, col=c(1,2), seg.len=1, merge=TRUE,
         bty='n')


## Plot differences (i.e. where the grammatical vs ungrammatical lines differ in this case for past)
plot_diff(Mod2, view="Time", cond=list(ISPresent=0, ISPresentUngrammatical=0), 
          comp=list(ISUngrammatical=c(0,1)), 
          rm.ranef = TRUE,
          main="Difference in Past grammaticality")
```

```
## Summary:
##  * Time : numeric predictor; with 100 values ranging from -195.000000 to 1195.000000. 
##  * ISPresent : numeric predictor; set to the value(s): 0. 
##  * ISPresentUngrammatical : numeric predictor; set to the value(s): 0. 
##  * Event : factor; set to the value(s): Frank01.Past_gram. (Might be canceled as random effect, check below.) 
##  * NOTE : The following random effects columns are canceled: s(Time,Event)
##
```

```
## 
## Difference is not significant.
```

```
#Plotting for Present
##Plot present grammatical
plot_smooth(Mod2, view="Time", rm.ranef=TRUE, 
            cond=list(ISPresent=1, ISUngrammatical=0,ISPresentUngrammatical=0),
            col=1, xpd=TRUE, eegAxis = TRUE, ylim = c(2, -2), main="Present: F4")
```

```
## Summary:
##  * Time : numeric predictor; with 30 values ranging from -195.000000 to 1195.000000. 
##  * ISPresent : numeric predictor; set to the value(s): 1. 
##  * ISUngrammatical : numeric predictor; set to the value(s): 0. 
##  * ISPresentUngrammatical : numeric predictor; set to the value(s): 0. 
##  * Event : factor; set to the value(s): Frank01.Past_gram. (Might be canceled as random effect, check below.) 
##  * NOTE : The following random effects columns are canceled: s(Time,Event)
##
```

```
##Plot present ungrammatical
plot_smooth(Mod2, view="Time", rm.ranef=TRUE, 
            cond=list(ISPresent=1, ISUngrammatical=1,ISPresentUngrammatical=1),
            add=TRUE, col=2, xpd=TRUE, main = "Present")
```

```
## Summary:
##  * Time : numeric predictor; with 30 values ranging from -195.000000 to 1195.000000. 
##  * ISPresent : numeric predictor; set to the value(s): 1. 
##  * ISUngrammatical : numeric predictor; set to the value(s): 1. 
##  * ISPresentUngrammatical : numeric predictor; set to the value(s): 1. 
##  * Event : factor; set to the value(s): Frank01.Past_gram. (Might be canceled as random effect, check below.) 
##  * NOTE : The following random effects columns are canceled: s(Time,Event)
##
```

```
## Plot differences (i.e. where the grammatical vs ungrammatical lines differ in this case for present)
plot_diff(Mod2, view="Time", cond=list(ISPresent=1), 
          comp=list(ISUngrammatical=c(0,1), ISPresentUngrammatical=c(0,1)), 
          rm.ranef = TRUE,
          main="Difference in Present grammaticality")
```

```
## Summary:
##  * Time : numeric predictor; with 100 values ranging from -195.000000 to 1195.000000. 
##  * ISPresent : numeric predictor; set to the value(s): 1. 
##  * Event : factor; set to the value(s): Frank01.Past_gram. (Might be canceled as random effect, check below.) 
##  * NOTE : The following random effects columns are canceled: s(Time,Event)
##
```

```
## 
## Difference is not significant.
```

#Subsetting data with electrode: F8

```
subdat <- droplevels(dat[dat$Channel== "F8" & dat$Condition %in% c("Past_gram", "Past_ungram", "Present_gram", "Present_ungram"),]) 
head(subdat)
```

```
##     Subject Condition Grammaticality Channel Time       value Tense
## 981 Frank01 Past_gram    Grammatical      F8 -195 -0.20158462  Past
## 982 Frank01 Past_gram    Grammatical      F8 -185  1.05760256  Past
## 983 Frank01 Past_gram    Grammatical      F8 -175  1.49192308  Past
## 984 Frank01 Past_gram    Grammatical      F8 -165  0.61856923  Past
## 985 Frank01 Past_gram    Grammatical      F8 -155 -0.05273077  Past
## 986 Frank01 Past_gram    Grammatical      F8 -145 -0.51407692  Past
##     ISPresent ISUngrammatical ISPresentUngrammatical
## 981         0               0                      0
## 982         0               0                      0
## 983         0               0                      0
## 984         0               0                      0
## 985         0               0                      0
## 986         0               0                      0
```

## Create EVENT (unique combination of Subject and Items)for random effects

```
subdat$Event <- interaction(subdat$Subject, subdat$Condition)

#   Determine starting point for each time series
subdat <- start_event(subdat,column = "Time",event = "Event")

# Make sure variables are factors
subdat$Condition <- as.factor(subdat$Condition) 
subdat$Subject <- as.factor(subdat$Subject)
```

# GAMM ANALYSIS

```
Mod1 <- bam(value ~ s(Time, k=20) 
            + s(Time, by=ISPresent, k=20)
            + s(Time, by=ISUngrammatical, k=20)
            + s(Time, by=ISPresentUngrammatical, k=20)
            + s(Time,Event, bs='fs', m=1, k=20),
            data=subdat, discrete = TRUE, family="scat")
```

```
## Warning in gam.side(sm, X, tol = .Machine$double.eps^0.5): model has
## repeated 1-d smooths of same variable.
```

```
## Calculate rho value to account for autocorrelation in data.
myrho <- start_value_rho(Mod1)

#Fit a Model including rho
Mod2 <- bam(value ~ s(Time, k=20) 
            + s(Time, by=ISPresent, k=20)
            + s(Time, by=ISUngrammatical, k=20)
            + s(Time, by=ISPresentUngrammatical, k=20)
            + s(Time,Event, bs='fs', m=1, k=20),
            data=subdat, discrete = TRUE, family="scat", 
            AR.start=subdat$start.event, rho=myrho)
```

```
## Warning in gam.side(sm, X, tol = .Machine$double.eps^0.5): model has
## repeated 1-d smooths of same variable.
```

```
#Plot model residuals
qqnorm(resid(Mod2))
qqline(resid(Mod2))
```

#PLOTTING OF GAM model

```
par(mfcol =c(2,2))

#Plotting Past
##Plot past grammatical
plot_smooth(Mod2, view="Time", rm.ranef=TRUE, 
            cond=list(ISPresent=0, ISUngrammatical=0,ISPresentUngrammatical=0),
            col=1, xpd=TRUE, eegAxis = TRUE, ylim = c(2, -2), main="Past: F8")
```

```
## Summary:
##  * Time : numeric predictor; with 30 values ranging from -195.000000 to 1195.000000. 
##  * ISPresent : numeric predictor; set to the value(s): 0. 
##  * ISUngrammatical : numeric predictor; set to the value(s): 0. 
##  * ISPresentUngrammatical : numeric predictor; set to the value(s): 0. 
##  * Event : factor; set to the value(s): Frank01.Past_gram. (Might be canceled as random effect, check below.) 
##  * NOTE : The following random effects columns are canceled: s(Time,Event)
##
```

```
##Plot past ungrammatical
plot_smooth(Mod2, view="Time", rm.ranef=TRUE, 
            cond=list(ISPresent=0, ISUngrammatical=1,ISPresentUngrammatical=0),
            h0=0,add=TRUE, v0=0,col=2)
```

```
## Summary:
##  * Time : numeric predictor; with 30 values ranging from -195.000000 to 1195.000000. 
##  * ISPresent : numeric predictor; set to the value(s): 0. 
##  * ISUngrammatical : numeric predictor; set to the value(s): 1. 
##  * ISPresentUngrammatical : numeric predictor; set to the value(s): 0. 
##  * Event : factor; set to the value(s): Frank01.Past_gram. (Might be canceled as random effect, check below.) 
##  * NOTE : The following random effects columns are canceled: s(Time,Event)
##
```

```
legend_margin('bottomleft', legend=c("grammatical", "ungrammatical"),
       fill=c(alpha(1), alpha(2)), border= c(alpha(1), alpha(2)),
         lwd=1.5, col=c(1,2), seg.len=1, merge=TRUE,
         bty='n')


## Plot differences (i.e. where the grammatical vs ungrammatical lines differ in this case for past)
plot_diff(Mod2, view="Time", cond=list(ISPresent=0, ISPresentUngrammatical=0), 
          comp=list(ISUngrammatical=c(0,1)), 
          rm.ranef = TRUE,
          main="Difference in Past grammaticality")
```

```
## Summary:
##  * Time : numeric predictor; with 100 values ranging from -195.000000 to 1195.000000. 
##  * ISPresent : numeric predictor; set to the value(s): 0. 
##  * ISPresentUngrammatical : numeric predictor; set to the value(s): 0. 
##  * Event : factor; set to the value(s): Frank01.Past_gram. (Might be canceled as random effect, check below.) 
##  * NOTE : The following random effects columns are canceled: s(Time,Event)
##
```

```
## 
## Difference is not significant.
```

```
#Plotting for Present
##Plot present grammatical
plot_smooth(Mod2, view="Time", rm.ranef=TRUE, 
            cond=list(ISPresent=1, ISUngrammatical=0,ISPresentUngrammatical=0),
            col=1, xpd=TRUE, eegAxis = TRUE, ylim = c(2, -2), main="Present: F8")
```

```
## Summary:
##  * Time : numeric predictor; with 30 values ranging from -195.000000 to 1195.000000. 
##  * ISPresent : numeric predictor; set to the value(s): 1. 
##  * ISUngrammatical : numeric predictor; set to the value(s): 0. 
##  * ISPresentUngrammatical : numeric predictor; set to the value(s): 0. 
##  * Event : factor; set to the value(s): Frank01.Past_gram. (Might be canceled as random effect, check below.) 
##  * NOTE : The following random effects columns are canceled: s(Time,Event)
##
```

```
##Plot present ungrammatical
plot_smooth(Mod2, view="Time", rm.ranef=TRUE, 
            cond=list(ISPresent=1, ISUngrammatical=1,ISPresentUngrammatical=1),
            add=TRUE, col=2, xpd=TRUE, main = "Present")
```

```
## Summary:
##  * Time : numeric predictor; with 30 values ranging from -195.000000 to 1195.000000. 
##  * ISPresent : numeric predictor; set to the value(s): 1. 
##  * ISUngrammatical : numeric predictor; set to the value(s): 1. 
##  * ISPresentUngrammatical : numeric predictor; set to the value(s): 1. 
##  * Event : factor; set to the value(s): Frank01.Past_gram. (Might be canceled as random effect, check below.) 
##  * NOTE : The following random effects columns are canceled: s(Time,Event)
##
```

```
## Plot differences (i.e. where the grammatical vs ungrammatical lines differ in this case for present)
plot_diff(Mod2, view="Time", cond=list(ISPresent=1), 
          comp=list(ISUngrammatical=c(0,1), ISPresentUngrammatical=c(0,1)), 
          rm.ranef = TRUE,
          main="Difference in Present grammaticality")
```

```
## Summary:
##  * Time : numeric predictor; with 100 values ranging from -195.000000 to 1195.000000. 
##  * ISPresent : numeric predictor; set to the value(s): 1. 
##  * Event : factor; set to the value(s): Frank01.Past_gram. (Might be canceled as random effect, check below.) 
##  * NOTE : The following random effects columns are canceled: s(Time,Event)
##
```

```
## 
## Difference is not significant.
```

#Subsetting data with electrode: FC5

```
subdat <- droplevels(dat[dat$Channel== "FC5" & dat$Condition %in% c("Past_gram", "Past_ungram", "Present_gram", "Present_ungram"),]) 
head(subdat)
```

```
##      Subject Condition Grammaticality Channel Time      value Tense
## 1121 Frank01 Past_gram    Grammatical     FC5 -195 -0.2518308  Past
## 1122 Frank01 Past_gram    Grammatical     FC5 -185  0.6551218  Past
## 1123 Frank01 Past_gram    Grammatical     FC5 -175  0.9476250  Past
## 1124 Frank01 Past_gram    Grammatical     FC5 -165  0.3059769  Past
## 1125 Frank01 Past_gram    Grammatical     FC5 -155 -0.3273397  Past
## 1126 Frank01 Past_gram    Grammatical     FC5 -145 -0.3007308  Past
##      ISPresent ISUngrammatical ISPresentUngrammatical
## 1121         0               0                      0
## 1122         0               0                      0
## 1123         0               0                      0
## 1124         0               0                      0
## 1125         0               0                      0
## 1126         0               0                      0
```

## Create EVENT (unique combination of Subject and Items)for random effects

```
subdat$Event <- interaction(subdat$Subject, subdat$Condition)

#   Determine starting point for each time series
subdat <- start_event(subdat,column = "Time",event = "Event")

# Make sure variables are factors
subdat$Condition <- as.factor(subdat$Condition) 
subdat$Subject <- as.factor(subdat$Subject)
```

# GAMM ANALYSIS

```
Mod1 <- bam(value ~ s(Time, k=20) 
            + s(Time, by=ISPresent, k=20)
            + s(Time, by=ISUngrammatical, k=20)
            + s(Time, by=ISPresentUngrammatical, k=20)
            + s(Time,Event, bs='fs', m=1, k=20),
            data=subdat, discrete = TRUE, family="scat")
```

```
## Warning in gam.side(sm, X, tol = .Machine$double.eps^0.5): model has
## repeated 1-d smooths of same variable.
```

```
## Calculate rho value to account for autocorrelation in data.
myrho <- start_value_rho(Mod1)

#Fit a Model including rho
Mod2 <- bam(value ~ s(Time, k=20) 
            + s(Time, by=ISPresent, k=20)
            + s(Time, by=ISUngrammatical, k=20)
            + s(Time, by=ISPresentUngrammatical, k=20)
            + s(Time,Event, bs='fs', m=1, k=20),
            data=subdat, discrete = TRUE, family="scat", 
            AR.start=subdat$start.event, rho=myrho)
```

```
## Warning in gam.side(sm, X, tol = .Machine$double.eps^0.5): model has
## repeated 1-d smooths of same variable.
```

```
## Warning in estimate.theta(theta, family, y, mu, scale = scale1, wt = G$w, :
## step failure in theta estimation
```

```
#Plot model residuals
qqnorm(resid(Mod2))
qqline(resid(Mod2))
```

#PLOTTING OF GAM model

```
par(mfcol =c(2,2))

#Plotting Past
##Plot past grammatical
plot_smooth(Mod2, view="Time", rm.ranef=TRUE, 
            cond=list(ISPresent=0, ISUngrammatical=0,ISPresentUngrammatical=0),
            col=1, xpd=TRUE, eegAxis = TRUE, ylim = c(2, -2), main="Past: FC5")
```

```
## Summary:
##  * Time : numeric predictor; with 30 values ranging from -195.000000 to 1195.000000. 
##  * ISPresent : numeric predictor; set to the value(s): 0. 
##  * ISUngrammatical : numeric predictor; set to the value(s): 0. 
##  * ISPresentUngrammatical : numeric predictor; set to the value(s): 0. 
##  * Event : factor; set to the value(s): Frank01.Past_gram. (Might be canceled as random effect, check below.) 
##  * NOTE : The following random effects columns are canceled: s(Time,Event)
##
```

```
##Plot past ungrammatical
plot_smooth(Mod2, view="Time", rm.ranef=TRUE, 
            cond=list(ISPresent=0, ISUngrammatical=1,ISPresentUngrammatical=0),
            h0=0,add=TRUE, v0=0,col=2)
```

```
## Summary:
##  * Time : numeric predictor; with 30 values ranging from -195.000000 to 1195.000000. 
##  * ISPresent : numeric predictor; set to the value(s): 0. 
##  * ISUngrammatical : numeric predictor; set to the value(s): 1. 
##  * ISPresentUngrammatical : numeric predictor; set to the value(s): 0. 
##  * Event : factor; set to the value(s): Frank01.Past_gram. (Might be canceled as random effect, check below.) 
##  * NOTE : The following random effects columns are canceled: s(Time,Event)
##
```

```
legend_margin('bottomleft', legend=c("grammatical", "ungrammatical"),
       fill=c(alpha(1), alpha(2)), border= c(alpha(1), alpha(2)),
         lwd=1.5, col=c(1,2), seg.len=1, merge=TRUE,
         bty='n')


## Plot differences (i.e. where the grammatical vs ungrammatical lines differ in this case for past)
plot_diff(Mod2, view="Time", cond=list(ISPresent=0, ISPresentUngrammatical=0), 
          comp=list(ISUngrammatical=c(0,1)), 
          rm.ranef = TRUE,
          main="Difference in Past grammaticality")
```

```
## Summary:
##  * Time : numeric predictor; with 100 values ranging from -195.000000 to 1195.000000. 
##  * ISPresent : numeric predictor; set to the value(s): 0. 
##  * ISPresentUngrammatical : numeric predictor; set to the value(s): 0. 
##  * Event : factor; set to the value(s): Frank01.Past_gram. (Might be canceled as random effect, check below.) 
##  * NOTE : The following random effects columns are canceled: s(Time,Event)
##
```

```
## 
## Difference is not significant.
```

```
#Plotting for Present
##Plot present grammatical
plot_smooth(Mod2, view="Time", rm.ranef=TRUE, 
            cond=list(ISPresent=1, ISUngrammatical=0,ISPresentUngrammatical=0),
            col=1, xpd=TRUE, eegAxis = TRUE, ylim = c(2, -2), main="Present: FC5")
```

```
## Summary:
##  * Time : numeric predictor; with 30 values ranging from -195.000000 to 1195.000000. 
##  * ISPresent : numeric predictor; set to the value(s): 1. 
##  * ISUngrammatical : numeric predictor; set to the value(s): 0. 
##  * ISPresentUngrammatical : numeric predictor; set to the value(s): 0. 
##  * Event : factor; set to the value(s): Frank01.Past_gram. (Might be canceled as random effect, check below.) 
##  * NOTE : The following random effects columns are canceled: s(Time,Event)
##
```

```
##Plot present ungrammatical
plot_smooth(Mod2, view="Time", rm.ranef=TRUE, 
            cond=list(ISPresent=1, ISUngrammatical=1,ISPresentUngrammatical=1),
            add=TRUE, col=2, xpd=TRUE, main = "Present")
```

```
## Summary:
##  * Time : numeric predictor; with 30 values ranging from -195.000000 to 1195.000000. 
##  * ISPresent : numeric predictor; set to the value(s): 1. 
##  * ISUngrammatical : numeric predictor; set to the value(s): 1. 
##  * ISPresentUngrammatical : numeric predictor; set to the value(s): 1. 
##  * Event : factor; set to the value(s): Frank01.Past_gram. (Might be canceled as random effect, check below.) 
##  * NOTE : The following random effects columns are canceled: s(Time,Event)
##
```

```
## Plot differences (i.e. where the grammatical vs ungrammatical lines differ in this case for present)
plot_diff(Mod2, view="Time", cond=list(ISPresent=1), 
          comp=list(ISUngrammatical=c(0,1), ISPresentUngrammatical=c(0,1)), 
          rm.ranef = TRUE,
          main="Difference in Present grammaticality")
```

```
## Summary:
##  * Time : numeric predictor; with 100 values ranging from -195.000000 to 1195.000000. 
##  * ISPresent : numeric predictor; set to the value(s): 1. 
##  * Event : factor; set to the value(s): Frank01.Past_gram. (Might be canceled as random effect, check below.) 
##  * NOTE : The following random effects columns are canceled: s(Time,Event)
##
```

```
## 
## Difference is not significant.
```

#Subsetting data with electrode: FC1

```
subdat <- droplevels(dat[dat$Channel== "FC1" & dat$Condition %in% c("Past_gram", "Past_ungram", "Present_gram", "Present_ungram"),]) 
head(subdat)
```

```
##      Subject Condition Grammaticality Channel Time      value Tense
## 1261 Frank01 Past_gram    Grammatical     FC1 -195 -0.5042231  Past
## 1262 Frank01 Past_gram    Grammatical     FC1 -185  0.1630256  Past
## 1263 Frank01 Past_gram    Grammatical     FC1 -175  0.9557692  Past
## 1264 Frank01 Past_gram    Grammatical     FC1 -165  0.6088923  Past
## 1265 Frank01 Past_gram    Grammatical     FC1 -155 -0.2026218  Past
## 1266 Frank01 Past_gram    Grammatical     FC1 -145 -0.8088365  Past
##      ISPresent ISUngrammatical ISPresentUngrammatical
## 1261         0               0                      0
## 1262         0               0                      0
## 1263         0               0                      0
## 1264         0               0                      0
## 1265         0               0                      0
## 1266         0               0                      0
```

## Create EVENT (unique combination of Subject and Items)for random effects

```
subdat$Event <- interaction(subdat$Subject, subdat$Condition)

#   Determine starting point for each time series
subdat <- start_event(subdat,column = "Time",event = "Event")

# Make sure variables are factors
subdat$Condition <- as.factor(subdat$Condition) 
subdat$Subject <- as.factor(subdat$Subject)
```

# GAMM ANALYSIS

```
Mod1 <- bam(value ~ s(Time, k=20) 
            + s(Time, by=ISPresent, k=20)
            + s(Time, by=ISUngrammatical, k=20)
            + s(Time, by=ISPresentUngrammatical, k=20)
            + s(Time,Event, bs='fs', m=1, k=20),
            data=subdat, discrete = TRUE, family="scat")
```

```
## Warning in gam.side(sm, X, tol = .Machine$double.eps^0.5): model has
## repeated 1-d smooths of same variable.
```

```
## Calculate rho value to account for autocorrelation in data.
myrho <- start_value_rho(Mod1)

#Fit a Model including rho
Mod2 <- bam(value ~ s(Time, k=20) 
            + s(Time, by=ISPresent, k=20)
            + s(Time, by=ISUngrammatical, k=20)
            + s(Time, by=ISPresentUngrammatical, k=20)
            + s(Time,Event, bs='fs', m=1, k=20),
            data=subdat, discrete = TRUE, family="scat", 
            AR.start=subdat$start.event, rho=myrho)
```

```
## Warning in gam.side(sm, X, tol = .Machine$double.eps^0.5): model has
## repeated 1-d smooths of same variable.
```

```
#Plot model residuals
qqnorm(resid(Mod2))
qqline(resid(Mod2))
```

#PLOTTING OF GAM model

```
par(mfcol =c(2,2))

#Plotting Past
##Plot past grammatical
plot_smooth(Mod2, view="Time", rm.ranef=TRUE, 
            cond=list(ISPresent=0, ISUngrammatical=0,ISPresentUngrammatical=0),
            col=1, xpd=TRUE, eegAxis = TRUE, ylim = c(2, -2), main="Past: FC1")
```

```
## Summary:
##  * Time : numeric predictor; with 30 values ranging from -195.000000 to 1195.000000. 
##  * ISPresent : numeric predictor; set to the value(s): 0. 
##  * ISUngrammatical : numeric predictor; set to the value(s): 0. 
##  * ISPresentUngrammatical : numeric predictor; set to the value(s): 0. 
##  * Event : factor; set to the value(s): Frank01.Past_gram. (Might be canceled as random effect, check below.) 
##  * NOTE : The following random effects columns are canceled: s(Time,Event)
##
```

```
##Plot past ungrammatical
plot_smooth(Mod2, view="Time", rm.ranef=TRUE, 
            cond=list(ISPresent=0, ISUngrammatical=1,ISPresentUngrammatical=0),
            h0=0,add=TRUE, v0=0,col=2)
```

```
## Summary:
##  * Time : numeric predictor; with 30 values ranging from -195.000000 to 1195.000000. 
##  * ISPresent : numeric predictor; set to the value(s): 0. 
##  * ISUngrammatical : numeric predictor; set to the value(s): 1. 
##  * ISPresentUngrammatical : numeric predictor; set to the value(s): 0. 
##  * Event : factor; set to the value(s): Frank01.Past_gram. (Might be canceled as random effect, check below.) 
##  * NOTE : The following random effects columns are canceled: s(Time,Event)
##
```

```
legend_margin('bottomleft', legend=c("grammatical", "ungrammatical"),
       fill=c(alpha(1), alpha(2)), border= c(alpha(1), alpha(2)),
         lwd=1.5, col=c(1,2), seg.len=1, merge=TRUE,
         bty='n')


## Plot differences (i.e. where the grammatical vs ungrammatical lines differ in this case for past)
plot_diff(Mod2, view="Time", cond=list(ISPresent=0, ISPresentUngrammatical=0), 
          comp=list(ISUngrammatical=c(0,1)), 
          rm.ranef = TRUE,
          main="Difference in Past grammaticality")
```

```
## Summary:
##  * Time : numeric predictor; with 100 values ranging from -195.000000 to 1195.000000. 
##  * ISPresent : numeric predictor; set to the value(s): 0. 
##  * ISPresentUngrammatical : numeric predictor; set to the value(s): 0. 
##  * Event : factor; set to the value(s): Frank01.Past_gram. (Might be canceled as random effect, check below.) 
##  * NOTE : The following random effects columns are canceled: s(Time,Event)
##
```

```
## 
## Time window(s) of significant difference(s):
##  478.939394 - 1195.000000
```

```
#Plotting for Present
##Plot present grammatical
plot_smooth(Mod2, view="Time", rm.ranef=TRUE, 
            cond=list(ISPresent=1, ISUngrammatical=0,ISPresentUngrammatical=0),
            col=1, xpd=TRUE, eegAxis = TRUE, ylim = c(2, -2), main="Present: FC1")
```

```
## Summary:
##  * Time : numeric predictor; with 30 values ranging from -195.000000 to 1195.000000. 
##  * ISPresent : numeric predictor; set to the value(s): 1. 
##  * ISUngrammatical : numeric predictor; set to the value(s): 0. 
##  * ISPresentUngrammatical : numeric predictor; set to the value(s): 0. 
##  * Event : factor; set to the value(s): Frank01.Past_gram. (Might be canceled as random effect, check below.) 
##  * NOTE : The following random effects columns are canceled: s(Time,Event)
##
```

```
##Plot present ungrammatical
plot_smooth(Mod2, view="Time", rm.ranef=TRUE, 
            cond=list(ISPresent=1, ISUngrammatical=1,ISPresentUngrammatical=1),
            add=TRUE, col=2, xpd=TRUE, main = "Present")
```

```
## Summary:
##  * Time : numeric predictor; with 30 values ranging from -195.000000 to 1195.000000. 
##  * ISPresent : numeric predictor; set to the value(s): 1. 
##  * ISUngrammatical : numeric predictor; set to the value(s): 1. 
##  * ISPresentUngrammatical : numeric predictor; set to the value(s): 1. 
##  * Event : factor; set to the value(s): Frank01.Past_gram. (Might be canceled as random effect, check below.) 
##  * NOTE : The following random effects columns are canceled: s(Time,Event)
##
```

```
## Plot differences (i.e. where the grammatical vs ungrammatical lines differ in this case for present)
plot_diff(Mod2, view="Time", cond=list(ISPresent=1), 
          comp=list(ISUngrammatical=c(0,1), ISPresentUngrammatical=c(0,1)), 
          rm.ranef = TRUE,
          main="Difference in Present grammaticality")
```

```
## Summary:
##  * Time : numeric predictor; with 100 values ranging from -195.000000 to 1195.000000. 
##  * ISPresent : numeric predictor; set to the value(s): 1. 
##  * Event : factor; set to the value(s): Frank01.Past_gram. (Might be canceled as random effect, check below.) 
##  * NOTE : The following random effects columns are canceled: s(Time,Event)
##
```

```
## 
## Difference is not significant.
```

#Subsetting data with electrode: FC2

```
subdat <- droplevels(dat[dat$Channel== "FC2" & dat$Condition %in% c("Past_gram", "Past_ungram", "Present_gram", "Present_ungram"),]) 
head(subdat)
```

```
##      Subject Condition Grammaticality Channel Time      value Tense
## 1401 Frank01 Past_gram    Grammatical     FC2 -195 -0.2644538  Past
## 1402 Frank01 Past_gram    Grammatical     FC2 -185  0.6145000  Past
## 1403 Frank01 Past_gram    Grammatical     FC2 -175  1.2729712  Past
## 1404 Frank01 Past_gram    Grammatical     FC2 -165  0.6258000  Past
## 1405 Frank01 Past_gram    Grammatical     FC2 -155 -0.2099679  Past
## 1406 Frank01 Past_gram    Grammatical     FC2 -145 -0.8728942  Past
##      ISPresent ISUngrammatical ISPresentUngrammatical
## 1401         0               0                      0
## 1402         0               0                      0
## 1403         0               0                      0
## 1404         0               0                      0
## 1405         0               0                      0
## 1406         0               0                      0
```

## Create EVENT (unique combination of Subject and Items)for random effects

```
subdat$Event <- interaction(subdat$Subject, subdat$Condition)

#   Determine starting point for each time series
subdat <- start_event(subdat,column = "Time",event = "Event")

# Make sure variables are factors
subdat$Condition <- as.factor(subdat$Condition) 
subdat$Subject <- as.factor(subdat$Subject)
```

# GAMM ANALYSIS

```
Mod1 <- bam(value ~ s(Time, k=20) 
            + s(Time, by=ISPresent, k=20)
            + s(Time, by=ISUngrammatical, k=20)
            + s(Time, by=ISPresentUngrammatical, k=20)
            + s(Time,Event, bs='fs', m=1, k=20),
            data=subdat, discrete = TRUE, family="scat")
```

```
## Warning in gam.side(sm, X, tol = .Machine$double.eps^0.5): model has
## repeated 1-d smooths of same variable.
```

```
## Calculate rho value to account for autocorrelation in data.
myrho <- start_value_rho(Mod1)

#Fit a Model including rho
Mod2 <- bam(value ~ s(Time, k=20) 
            + s(Time, by=ISPresent, k=20)
            + s(Time, by=ISUngrammatical, k=20)
            + s(Time, by=ISPresentUngrammatical, k=20)
            + s(Time,Event, bs='fs', m=1, k=20),
            data=subdat, discrete = TRUE, family="scat", 
            AR.start=subdat$start.event, rho=myrho)
```

```
## Warning in gam.side(sm, X, tol = .Machine$double.eps^0.5): model has
## repeated 1-d smooths of same variable.
```

```
#Plot model residuals
qqnorm(resid(Mod2))
qqline(resid(Mod2))
```

#PLOTTING OF GAM model

```
par(mfcol =c(2,2))

#Plotting Past
##Plot past grammatical
plot_smooth(Mod2, view="Time", rm.ranef=TRUE, 
            cond=list(ISPresent=0, ISUngrammatical=0,ISPresentUngrammatical=0),
            col=1, xpd=TRUE, eegAxis = TRUE, ylim = c(2, -2), main="Past: FC2")
```

```
## Summary:
##  * Time : numeric predictor; with 30 values ranging from -195.000000 to 1195.000000. 
##  * ISPresent : numeric predictor; set to the value(s): 0. 
##  * ISUngrammatical : numeric predictor; set to the value(s): 0. 
##  * ISPresentUngrammatical : numeric predictor; set to the value(s): 0. 
##  * Event : factor; set to the value(s): Frank01.Past_gram. (Might be canceled as random effect, check below.) 
##  * NOTE : The following random effects columns are canceled: s(Time,Event)
##
```

```
##Plot past ungrammatical
plot_smooth(Mod2, view="Time", rm.ranef=TRUE, 
            cond=list(ISPresent=0, ISUngrammatical=1,ISPresentUngrammatical=0),
            h0=0,add=TRUE, v0=0,col=2)
```

```
## Summary:
##  * Time : numeric predictor; with 30 values ranging from -195.000000 to 1195.000000. 
##  * ISPresent : numeric predictor; set to the value(s): 0. 
##  * ISUngrammatical : numeric predictor; set to the value(s): 1. 
##  * ISPresentUngrammatical : numeric predictor; set to the value(s): 0. 
##  * Event : factor; set to the value(s): Frank01.Past_gram. (Might be canceled as random effect, check below.) 
##  * NOTE : The following random effects columns are canceled: s(Time,Event)
##
```

```
legend_margin('bottomleft', legend=c("grammatical", "ungrammatical"),
       fill=c(alpha(1), alpha(2)), border= c(alpha(1), alpha(2)),
         lwd=1.5, col=c(1,2), seg.len=1, merge=TRUE,
         bty='n')


## Plot differences (i.e. where the grammatical vs ungrammatical lines differ in this case for past)
plot_diff(Mod2, view="Time", cond=list(ISPresent=0, ISPresentUngrammatical=0), 
          comp=list(ISUngrammatical=c(0,1)), 
          rm.ranef = TRUE,
          main="Difference in Past grammaticality")
```

```
## Summary:
##  * Time : numeric predictor; with 100 values ranging from -195.000000 to 1195.000000. 
##  * ISPresent : numeric predictor; set to the value(s): 0. 
##  * ISPresentUngrammatical : numeric predictor; set to the value(s): 0. 
##  * Event : factor; set to the value(s): Frank01.Past_gram. (Might be canceled as random effect, check below.) 
##  * NOTE : The following random effects columns are canceled: s(Time,Event)
##
```

```
## 
## Time window(s) of significant difference(s):
##  633.383838 - 1195.000000
```

```
#Plotting for Present
##Plot present grammatical
plot_smooth(Mod2, view="Time", rm.ranef=TRUE, 
            cond=list(ISPresent=1, ISUngrammatical=0,ISPresentUngrammatical=0),
            col=1, xpd=TRUE, eegAxis = TRUE, ylim = c(2, -2), main="Present: FC2")
```

```
## Summary:
##  * Time : numeric predictor; with 30 values ranging from -195.000000 to 1195.000000. 
##  * ISPresent : numeric predictor; set to the value(s): 1. 
##  * ISUngrammatical : numeric predictor; set to the value(s): 0. 
##  * ISPresentUngrammatical : numeric predictor; set to the value(s): 0. 
##  * Event : factor; set to the value(s): Frank01.Past_gram. (Might be canceled as random effect, check below.) 
##  * NOTE : The following random effects columns are canceled: s(Time,Event)
##
```

```
##Plot present ungrammatical
plot_smooth(Mod2, view="Time", rm.ranef=TRUE, 
            cond=list(ISPresent=1, ISUngrammatical=1,ISPresentUngrammatical=1),
            add=TRUE, col=2, xpd=TRUE, main = "Present")
```

```
## Summary:
##  * Time : numeric predictor; with 30 values ranging from -195.000000 to 1195.000000. 
##  * ISPresent : numeric predictor; set to the value(s): 1. 
##  * ISUngrammatical : numeric predictor; set to the value(s): 1. 
##  * ISPresentUngrammatical : numeric predictor; set to the value(s): 1. 
##  * Event : factor; set to the value(s): Frank01.Past_gram. (Might be canceled as random effect, check below.) 
##  * NOTE : The following random effects columns are canceled: s(Time,Event)
##
```

```
## Plot differences (i.e. where the grammatical vs ungrammatical lines differ in this case for present)
plot_diff(Mod2, view="Time", cond=list(ISPresent=1), 
          comp=list(ISUngrammatical=c(0,1), ISPresentUngrammatical=c(0,1)), 
          rm.ranef = TRUE,
          main="Difference in Present grammaticality")
```

```
## Summary:
##  * Time : numeric predictor; with 100 values ranging from -195.000000 to 1195.000000. 
##  * ISPresent : numeric predictor; set to the value(s): 1. 
##  * Event : factor; set to the value(s): Frank01.Past_gram. (Might be canceled as random effect, check below.) 
##  * NOTE : The following random effects columns are canceled: s(Time,Event)
##
```

```
## 
## Difference is not significant.
```

#Subsetting data with electrode: FC6

```
subdat <- droplevels(dat[dat$Channel== "FC6" & dat$Condition %in% c("Past_gram", "Past_ungram", "Present_gram", "Present_ungram"),]) 
head(subdat)
```

```
##      Subject Condition Grammaticality Channel Time      value Tense
## 1541 Frank01 Past_gram    Grammatical     FC6 -195 -0.4230923  Past
## 1542 Frank01 Past_gram    Grammatical     FC6 -185  0.6584359  Past
## 1543 Frank01 Past_gram    Grammatical     FC6 -175  1.0729231  Past
## 1544 Frank01 Past_gram    Grammatical     FC6 -165  0.2195000  Past
## 1545 Frank01 Past_gram    Grammatical     FC6 -155 -0.4885897  Past
## 1546 Frank01 Past_gram    Grammatical     FC6 -145 -0.7782308  Past
##      ISPresent ISUngrammatical ISPresentUngrammatical
## 1541         0               0                      0
## 1542         0               0                      0
## 1543         0               0                      0
## 1544         0               0                      0
## 1545         0               0                      0
## 1546         0               0                      0
```

## Create EVENT (unique combination of Subject and Items)for random effects

```
subdat$Event <- interaction(subdat$Subject, subdat$Condition)

#   Determine starting point for each time series
subdat <- start_event(subdat,column = "Time",event = "Event")

# Make sure variables are factors
subdat$Condition <- as.factor(subdat$Condition) 
subdat$Subject <- as.factor(subdat$Subject)
```

# GAMM ANALYSIS

```
Mod1 <- bam(value ~ s(Time, k=20) 
            + s(Time, by=ISPresent, k=20)
            + s(Time, by=ISUngrammatical, k=20)
            + s(Time, by=ISPresentUngrammatical, k=20)
            + s(Time,Event, bs='fs', m=1, k=20),
            data=subdat, discrete = TRUE, family="scat")
```

```
## Warning in gam.side(sm, X, tol = .Machine$double.eps^0.5): model has
## repeated 1-d smooths of same variable.
```

```
## Calculate rho value to account for autocorrelation in data.
myrho <- start_value_rho(Mod1)

#Fit a Model including rho
Mod2 <- bam(value ~ s(Time, k=20) 
            + s(Time, by=ISPresent, k=20)
            + s(Time, by=ISUngrammatical, k=20)
            + s(Time, by=ISPresentUngrammatical, k=20)
            + s(Time,Event, bs='fs', m=1, k=20),
            data=subdat, discrete = TRUE, family="scat", 
            AR.start=subdat$start.event, rho=myrho)
```

```
## Warning in gam.side(sm, X, tol = .Machine$double.eps^0.5): model has
## repeated 1-d smooths of same variable.
```

```
## Warning in estimate.theta(theta, family, y, mu, scale = scale1, wt = G$w, :
## step failure in theta estimation

## Warning in estimate.theta(theta, family, y, mu, scale = scale1, wt = G$w, :
## step failure in theta estimation
```

```
#Plot model residuals
qqnorm(resid(Mod2))
qqline(resid(Mod2))
```

#PLOTTING OF GAM model

```
par(mfcol =c(2,2))

#Plotting Past
##Plot past grammatical
plot_smooth(Mod2, view="Time", rm.ranef=TRUE, 
            cond=list(ISPresent=0, ISUngrammatical=0,ISPresentUngrammatical=0),
            col=1, xpd=TRUE, eegAxis = TRUE, ylim = c(2, -2), main="Past: FC6")
```

```
## Summary:
##  * Time : numeric predictor; with 30 values ranging from -195.000000 to 1195.000000. 
##  * ISPresent : numeric predictor; set to the value(s): 0. 
##  * ISUngrammatical : numeric predictor; set to the value(s): 0. 
##  * ISPresentUngrammatical : numeric predictor; set to the value(s): 0. 
##  * Event : factor; set to the value(s): Frank01.Past_gram. (Might be canceled as random effect, check below.) 
##  * NOTE : The following random effects columns are canceled: s(Time,Event)
##
```

```
##Plot past ungrammatical
plot_smooth(Mod2, view="Time", rm.ranef=TRUE, 
            cond=list(ISPresent=0, ISUngrammatical=1,ISPresentUngrammatical=0),
            h0=0,add=TRUE, v0=0,col=2)
```

```
## Summary:
##  * Time : numeric predictor; with 30 values ranging from -195.000000 to 1195.000000. 
##  * ISPresent : numeric predictor; set to the value(s): 0. 
##  * ISUngrammatical : numeric predictor; set to the value(s): 1. 
##  * ISPresentUngrammatical : numeric predictor; set to the value(s): 0. 
##  * Event : factor; set to the value(s): Frank01.Past_gram. (Might be canceled as random effect, check below.) 
##  * NOTE : The following random effects columns are canceled: s(Time,Event)
##
```

```
legend_margin('bottomleft', legend=c("grammatical", "ungrammatical"),
       fill=c(alpha(1), alpha(2)), border= c(alpha(1), alpha(2)),
         lwd=1.5, col=c(1,2), seg.len=1, merge=TRUE,
         bty='n')


## Plot differences (i.e. where the grammatical vs ungrammatical lines differ in this case for past)
plot_diff(Mod2, view="Time", cond=list(ISPresent=0, ISPresentUngrammatical=0), 
          comp=list(ISUngrammatical=c(0,1)), 
          rm.ranef = TRUE,
          main="Difference in Past grammaticality")
```

```
## Summary:
##  * Time : numeric predictor; with 100 values ranging from -195.000000 to 1195.000000. 
##  * ISPresent : numeric predictor; set to the value(s): 0. 
##  * ISPresentUngrammatical : numeric predictor; set to the value(s): 0. 
##  * Event : factor; set to the value(s): Frank01.Past_gram. (Might be canceled as random effect, check below.) 
##  * NOTE : The following random effects columns are canceled: s(Time,Event)
##
```

```
## 
## Difference is not significant.
```

```
#Plotting for Present
##Plot present grammatical
plot_smooth(Mod2, view="Time", rm.ranef=TRUE, 
            cond=list(ISPresent=1, ISUngrammatical=0,ISPresentUngrammatical=0),
            col=1, xpd=TRUE, eegAxis = TRUE, ylim = c(2, -2), main="Present: FC6")
```

```
## Summary:
##  * Time : numeric predictor; with 30 values ranging from -195.000000 to 1195.000000. 
##  * ISPresent : numeric predictor; set to the value(s): 1. 
##  * ISUngrammatical : numeric predictor; set to the value(s): 0. 
##  * ISPresentUngrammatical : numeric predictor; set to the value(s): 0. 
##  * Event : factor; set to the value(s): Frank01.Past_gram. (Might be canceled as random effect, check below.) 
##  * NOTE : The following random effects columns are canceled: s(Time,Event)
##
```

```
##Plot present ungrammatical
plot_smooth(Mod2, view="Time", rm.ranef=TRUE, 
            cond=list(ISPresent=1, ISUngrammatical=1,ISPresentUngrammatical=1),
            add=TRUE, col=2, xpd=TRUE, main = "Present")
```

```
## Summary:
##  * Time : numeric predictor; with 30 values ranging from -195.000000 to 1195.000000. 
##  * ISPresent : numeric predictor; set to the value(s): 1. 
##  * ISUngrammatical : numeric predictor; set to the value(s): 1. 
##  * ISPresentUngrammatical : numeric predictor; set to the value(s): 1. 
##  * Event : factor; set to the value(s): Frank01.Past_gram. (Might be canceled as random effect, check below.) 
##  * NOTE : The following random effects columns are canceled: s(Time,Event)
##
```

```
## Plot differences (i.e. where the grammatical vs ungrammatical lines differ in this case for present)
plot_diff(Mod2, view="Time", cond=list(ISPresent=1), 
          comp=list(ISUngrammatical=c(0,1), ISPresentUngrammatical=c(0,1)), 
          rm.ranef = TRUE,
          main="Difference in Present grammaticality")
```

```
## Summary:
##  * Time : numeric predictor; with 100 values ranging from -195.000000 to 1195.000000. 
##  * ISPresent : numeric predictor; set to the value(s): 1. 
##  * Event : factor; set to the value(s): Frank01.Past_gram. (Might be canceled as random effect, check below.) 
##  * NOTE : The following random effects columns are canceled: s(Time,Event)
##
```

```
## 
## Difference is not significant.
```

#Subsetting data with electrode: T7

```
subdat <- droplevels(dat[dat$Channel== "T7" & dat$Condition %in% c("Past_gram", "Past_ungram", "Present_gram", "Present_ungram"),]) 
head(subdat)
```

```
##      Subject Condition Grammaticality Channel Time       value Tense
## 1681 Frank01 Past_gram    Grammatical      T7 -195 -0.08862308  Past
## 1682 Frank01 Past_gram    Grammatical      T7 -185  0.14988462  Past
## 1683 Frank01 Past_gram    Grammatical      T7 -175  0.05545192  Past
## 1684 Frank01 Past_gram    Grammatical      T7 -165 -0.05883077  Past
## 1685 Frank01 Past_gram    Grammatical      T7 -155 -0.11289744  Past
## 1686 Frank01 Past_gram    Grammatical      T7 -145 -0.17272115  Past
##      ISPresent ISUngrammatical ISPresentUngrammatical
## 1681         0               0                      0
## 1682         0               0                      0
## 1683         0               0                      0
## 1684         0               0                      0
## 1685         0               0                      0
## 1686         0               0                      0
```

## Create EVENT (unique combination of Subject and Items)for random effects

```
subdat$Event <- interaction(subdat$Subject, subdat$Condition)

#   Determine starting point for each time series
subdat <- start_event(subdat,column = "Time",event = "Event")

# Make sure variables are factors
subdat$Condition <- as.factor(subdat$Condition) 
subdat$Subject <- as.factor(subdat$Subject)
```

# GAMM ANALYSIS

```
Mod1 <- bam(value ~ s(Time, k=20) 
            + s(Time, by=ISPresent, k=20)
            + s(Time, by=ISUngrammatical, k=20)
            + s(Time, by=ISPresentUngrammatical, k=20)
            + s(Time,Event, bs='fs', m=1, k=20),
            data=subdat, discrete = TRUE, family="scat")
```

```
## Warning in gam.side(sm, X, tol = .Machine$double.eps^0.5): model has
## repeated 1-d smooths of same variable.
```

```
## Calculate rho value to account for autocorrelation in data.
myrho <- start_value_rho(Mod1)

#Fit a Model including rho
Mod2 <- bam(value ~ s(Time, k=20) 
            + s(Time, by=ISPresent, k=20)
            + s(Time, by=ISUngrammatical, k=20)
            + s(Time, by=ISPresentUngrammatical, k=20)
            + s(Time,Event, bs='fs', m=1, k=20),
            data=subdat, discrete = TRUE, family="scat", 
            AR.start=subdat$start.event, rho=myrho)
```

```
## Warning in gam.side(sm, X, tol = .Machine$double.eps^0.5): model has
## repeated 1-d smooths of same variable.
```

```
#Plot model residuals
qqnorm(resid(Mod2))
qqline(resid(Mod2))
```

#PLOTTING OF GAM model

```
par(mfcol =c(2,2))

#Plotting Past
##Plot past grammatical
plot_smooth(Mod2, view="Time", rm.ranef=TRUE, 
            cond=list(ISPresent=0, ISUngrammatical=0,ISPresentUngrammatical=0),
            col=1, xpd=TRUE, eegAxis = TRUE, ylim = c(2, -2), main="Past: T7")
```

```
## Summary:
##  * Time : numeric predictor; with 30 values ranging from -195.000000 to 1195.000000. 
##  * ISPresent : numeric predictor; set to the value(s): 0. 
##  * ISUngrammatical : numeric predictor; set to the value(s): 0. 
##  * ISPresentUngrammatical : numeric predictor; set to the value(s): 0. 
##  * Event : factor; set to the value(s): Frank01.Past_gram. (Might be canceled as random effect, check below.) 
##  * NOTE : The following random effects columns are canceled: s(Time,Event)
##
```

```
##Plot past ungrammatical
plot_smooth(Mod2, view="Time", rm.ranef=TRUE, 
            cond=list(ISPresent=0, ISUngrammatical=1,ISPresentUngrammatical=0),
            h0=0,add=TRUE, v0=0,col=2)
```

```
## Summary:
##  * Time : numeric predictor; with 30 values ranging from -195.000000 to 1195.000000. 
##  * ISPresent : numeric predictor; set to the value(s): 0. 
##  * ISUngrammatical : numeric predictor; set to the value(s): 1. 
##  * ISPresentUngrammatical : numeric predictor; set to the value(s): 0. 
##  * Event : factor; set to the value(s): Frank01.Past_gram. (Might be canceled as random effect, check below.) 
##  * NOTE : The following random effects columns are canceled: s(Time,Event)
##
```

```
legend_margin('bottomleft', legend=c("grammatical", "ungrammatical"),
       fill=c(alpha(1), alpha(2)), border= c(alpha(1), alpha(2)),
         lwd=1.5, col=c(1,2), seg.len=1, merge=TRUE,
         bty='n')


## Plot differences (i.e. where the grammatical vs ungrammatical lines differ in this case for past)
plot_diff(Mod2, view="Time", cond=list(ISPresent=0, ISPresentUngrammatical=0), 
          comp=list(ISUngrammatical=c(0,1)), 
          rm.ranef = TRUE,
          main="Difference in Past grammaticality")
```

```
## Summary:
##  * Time : numeric predictor; with 100 values ranging from -195.000000 to 1195.000000. 
##  * ISPresent : numeric predictor; set to the value(s): 0. 
##  * ISPresentUngrammatical : numeric predictor; set to the value(s): 0. 
##  * Event : factor; set to the value(s): Frank01.Past_gram. (Might be canceled as random effect, check below.) 
##  * NOTE : The following random effects columns are canceled: s(Time,Event)
##
```

```
## 
## Difference is not significant.
```

```
#Plotting for Present
##Plot present grammatical
plot_smooth(Mod2, view="Time", rm.ranef=TRUE, 
            cond=list(ISPresent=1, ISUngrammatical=0,ISPresentUngrammatical=0),
            col=1, xpd=TRUE, eegAxis = TRUE, ylim = c(2, -2), main="Present: T7")
```

```
## Summary:
##  * Time : numeric predictor; with 30 values ranging from -195.000000 to 1195.000000. 
##  * ISPresent : numeric predictor; set to the value(s): 1. 
##  * ISUngrammatical : numeric predictor; set to the value(s): 0. 
##  * ISPresentUngrammatical : numeric predictor; set to the value(s): 0. 
##  * Event : factor; set to the value(s): Frank01.Past_gram. (Might be canceled as random effect, check below.) 
##  * NOTE : The following random effects columns are canceled: s(Time,Event)
##
```

```
##Plot present ungrammatical
plot_smooth(Mod2, view="Time", rm.ranef=TRUE, 
            cond=list(ISPresent=1, ISUngrammatical=1,ISPresentUngrammatical=1),
            add=TRUE, col=2, xpd=TRUE, main = "Present")
```

```
## Summary:
##  * Time : numeric predictor; with 30 values ranging from -195.000000 to 1195.000000. 
##  * ISPresent : numeric predictor; set to the value(s): 1. 
##  * ISUngrammatical : numeric predictor; set to the value(s): 1. 
##  * ISPresentUngrammatical : numeric predictor; set to the value(s): 1. 
##  * Event : factor; set to the value(s): Frank01.Past_gram. (Might be canceled as random effect, check below.) 
##  * NOTE : The following random effects columns are canceled: s(Time,Event)
##
```

```
## Plot differences (i.e. where the grammatical vs ungrammatical lines differ in this case for present)
plot_diff(Mod2, view="Time", cond=list(ISPresent=1), 
          comp=list(ISUngrammatical=c(0,1), ISPresentUngrammatical=c(0,1)), 
          rm.ranef = TRUE,
          main="Difference in Present grammaticality")
```

```
## Summary:
##  * Time : numeric predictor; with 100 values ranging from -195.000000 to 1195.000000. 
##  * ISPresent : numeric predictor; set to the value(s): 1. 
##  * Event : factor; set to the value(s): Frank01.Past_gram. (Might be canceled as random effect, check below.) 
##  * NOTE : The following random effects columns are canceled: s(Time,Event)
##
```

```
## 
## Difference is not significant.
```

#Subsetting data with electrode: C4

```
subdat <- droplevels(dat[dat$Channel== "C4" & dat$Condition %in% c("Past_gram", "Past_ungram", "Present_gram", "Present_ungram"),]) 
head(subdat)
```

```
##      Subject Condition Grammaticality Channel Time       value Tense
## 2101 Frank01 Past_gram    Grammatical      C4 -195  0.09855385  Past
## 2102 Frank01 Past_gram    Grammatical      C4 -185  0.77789744  Past
## 2103 Frank01 Past_gram    Grammatical      C4 -175  1.00086538  Past
## 2104 Frank01 Past_gram    Grammatical      C4 -165  0.36100000  Past
## 2105 Frank01 Past_gram    Grammatical      C4 -155 -0.17734615  Past
## 2106 Frank01 Past_gram    Grammatical      C4 -145 -0.75882692  Past
##      ISPresent ISUngrammatical ISPresentUngrammatical
## 2101         0               0                      0
## 2102         0               0                      0
## 2103         0               0                      0
## 2104         0               0                      0
## 2105         0               0                      0
## 2106         0               0                      0
```

## Create EVENT (unique combination of Subject and Items)for random effects

```
subdat$Event <- interaction(subdat$Subject, subdat$Condition)

#   Determine starting point for each time series
subdat <- start_event(subdat,column = "Time",event = "Event")

# Make sure variables are factors
subdat$Condition <- as.factor(subdat$Condition) 
subdat$Subject <- as.factor(subdat$Subject)
```

# GAMM ANALYSIS

```
Mod1 <- bam(value ~ s(Time, k=20) 
            + s(Time, by=ISPresent, k=20)
            + s(Time, by=ISUngrammatical, k=20)
            + s(Time, by=ISPresentUngrammatical, k=20)
            + s(Time,Event, bs='fs', m=1, k=20),
            data=subdat, discrete = TRUE, family="scat")
```

```
## Warning in gam.side(sm, X, tol = .Machine$double.eps^0.5): model has
## repeated 1-d smooths of same variable.
```

```
## Calculate rho value to account for autocorrelation in data.
myrho <- start_value_rho(Mod1)

#Fit a Model including rho
Mod2 <- bam(value ~ s(Time, k=20) 
            + s(Time, by=ISPresent, k=20)
            + s(Time, by=ISUngrammatical, k=20)
            + s(Time, by=ISPresentUngrammatical, k=20)
            + s(Time,Event, bs='fs', m=1, k=20),
            data=subdat, discrete = TRUE, family="scat", 
            AR.start=subdat$start.event, rho=myrho)
```

```
## Warning in gam.side(sm, X, tol = .Machine$double.eps^0.5): model has
## repeated 1-d smooths of same variable.
```

```
#Plot model residuals
qqnorm(resid(Mod2))
qqline(resid(Mod2))
```

#PLOTTING OF GAM model

```
par(mfcol =c(2,2))

#Plotting Past
##Plot past grammatical
plot_smooth(Mod2, view="Time", rm.ranef=TRUE, 
            cond=list(ISPresent=0, ISUngrammatical=0,ISPresentUngrammatical=0),
            col=1, xpd=TRUE, eegAxis = TRUE, ylim = c(2, -2), main="Past: C4")
```

```
## Summary:
##  * Time : numeric predictor; with 30 values ranging from -195.000000 to 1195.000000. 
##  * ISPresent : numeric predictor; set to the value(s): 0. 
##  * ISUngrammatical : numeric predictor; set to the value(s): 0. 
##  * ISPresentUngrammatical : numeric predictor; set to the value(s): 0. 
##  * Event : factor; set to the value(s): Frank01.Past_gram. (Might be canceled as random effect, check below.) 
##  * NOTE : The following random effects columns are canceled: s(Time,Event)
##
```

```
##Plot past ungrammatical
plot_smooth(Mod2, view="Time", rm.ranef=TRUE, 
            cond=list(ISPresent=0, ISUngrammatical=1,ISPresentUngrammatical=0),
            h0=0,add=TRUE, v0=0,col=2)
```

```
## Summary:
##  * Time : numeric predictor; with 30 values ranging from -195.000000 to 1195.000000. 
##  * ISPresent : numeric predictor; set to the value(s): 0. 
##  * ISUngrammatical : numeric predictor; set to the value(s): 1. 
##  * ISPresentUngrammatical : numeric predictor; set to the value(s): 0. 
##  * Event : factor; set to the value(s): Frank01.Past_gram. (Might be canceled as random effect, check below.) 
##  * NOTE : The following random effects columns are canceled: s(Time,Event)
##
```

```
legend_margin('bottomleft', legend=c("grammatical", "ungrammatical"),
       fill=c(alpha(1), alpha(2)), border= c(alpha(1), alpha(2)),
         lwd=1.5, col=c(1,2), seg.len=1, merge=TRUE,
         bty='n')


## Plot differences (i.e. where the grammatical vs ungrammatical lines differ in this case for past)
plot_diff(Mod2, view="Time", cond=list(ISPresent=0, ISPresentUngrammatical=0), 
          comp=list(ISUngrammatical=c(0,1)), 
          rm.ranef = TRUE,
          main="Difference in Past grammaticality")
```

```
## Summary:
##  * Time : numeric predictor; with 100 values ranging from -195.000000 to 1195.000000. 
##  * ISPresent : numeric predictor; set to the value(s): 0. 
##  * ISPresentUngrammatical : numeric predictor; set to the value(s): 0. 
##  * Event : factor; set to the value(s): Frank01.Past_gram. (Might be canceled as random effect, check below.) 
##  * NOTE : The following random effects columns are canceled: s(Time,Event)
##
```

```
## 
## Difference is not significant.
```

```
#Plotting for Present
##Plot present grammatical
plot_smooth(Mod2, view="Time", rm.ranef=TRUE, 
            cond=list(ISPresent=1, ISUngrammatical=0,ISPresentUngrammatical=0),
            col=1, xpd=TRUE, eegAxis = TRUE, ylim = c(2, -2), main="Present: C4")
```

```
## Summary:
##  * Time : numeric predictor; with 30 values ranging from -195.000000 to 1195.000000. 
##  * ISPresent : numeric predictor; set to the value(s): 1. 
##  * ISUngrammatical : numeric predictor; set to the value(s): 0. 
##  * ISPresentUngrammatical : numeric predictor; set to the value(s): 0. 
##  * Event : factor; set to the value(s): Frank01.Past_gram. (Might be canceled as random effect, check below.) 
##  * NOTE : The following random effects columns are canceled: s(Time,Event)
##
```

```
##Plot present ungrammatical
plot_smooth(Mod2, view="Time", rm.ranef=TRUE, 
            cond=list(ISPresent=1, ISUngrammatical=1,ISPresentUngrammatical=1),
            add=TRUE, col=2, xpd=TRUE, main = "Present")
```

```
## Summary:
##  * Time : numeric predictor; with 30 values ranging from -195.000000 to 1195.000000. 
##  * ISPresent : numeric predictor; set to the value(s): 1. 
##  * ISUngrammatical : numeric predictor; set to the value(s): 1. 
##  * ISPresentUngrammatical : numeric predictor; set to the value(s): 1. 
##  * Event : factor; set to the value(s): Frank01.Past_gram. (Might be canceled as random effect, check below.) 
##  * NOTE : The following random effects columns are canceled: s(Time,Event)
##
```

```
## Plot differences (i.e. where the grammatical vs ungrammatical lines differ in this case for present)
plot_diff(Mod2, view="Time", cond=list(ISPresent=1), 
          comp=list(ISUngrammatical=c(0,1), ISPresentUngrammatical=c(0,1)), 
          rm.ranef = TRUE,
          main="Difference in Present grammaticality")
```

```
## Summary:
##  * Time : numeric predictor; with 100 values ranging from -195.000000 to 1195.000000. 
##  * ISPresent : numeric predictor; set to the value(s): 1. 
##  * Event : factor; set to the value(s): Frank01.Past_gram. (Might be canceled as random effect, check below.) 
##  * NOTE : The following random effects columns are canceled: s(Time,Event)
##
```

```
## 
## Difference is not significant.
```

#Subsetting data with electrode: T8

```
subdat <- droplevels(dat[dat$Channel== "T8" & dat$Condition %in% c("Past_gram", "Past_ungram", "Present_gram", "Present_ungram"),]) 
head(subdat)
```

```
##      Subject Condition Grammaticality Channel Time      value Tense
## 2241 Frank01 Past_gram    Grammatical      T8 -195  0.4827000  Past
## 2242 Frank01 Past_gram    Grammatical      T8 -185  1.0796218  Past
## 2243 Frank01 Past_gram    Grammatical      T8 -175  0.7911442  Past
## 2244 Frank01 Past_gram    Grammatical      T8 -165  0.5321154  Past
## 2245 Frank01 Past_gram    Grammatical      T8 -155  0.2923718  Past
## 2246 Frank01 Past_gram    Grammatical      T8 -145 -0.5704231  Past
##      ISPresent ISUngrammatical ISPresentUngrammatical
## 2241         0               0                      0
## 2242         0               0                      0
## 2243         0               0                      0
## 2244         0               0                      0
## 2245         0               0                      0
## 2246         0               0                      0
```

## Create EVENT (unique combination of Subject and Items)for random effects

```
subdat$Event <- interaction(subdat$Subject, subdat$Condition)

#   Determine starting point for each time series
subdat <- start_event(subdat,column = "Time",event = "Event")

# Make sure variables are factors
subdat$Condition <- as.factor(subdat$Condition) 
subdat$Subject <- as.factor(subdat$Subject)
```

# GAMM ANALYSIS

```
Mod1 <- bam(value ~ s(Time, k=20) 
            + s(Time, by=ISPresent, k=20)
            + s(Time, by=ISUngrammatical, k=20)
            + s(Time, by=ISPresentUngrammatical, k=20)
            + s(Time,Event, bs='fs', m=1, k=20),
            data=subdat, discrete = TRUE, family="scat")
```

```
## Warning in gam.side(sm, X, tol = .Machine$double.eps^0.5): model has
## repeated 1-d smooths of same variable.
```

```
## Calculate rho value to account for autocorrelation in data.
myrho <- start_value_rho(Mod1)

#Fit a Model including rho
Mod2 <- bam(value ~ s(Time, k=20) 
            + s(Time, by=ISPresent, k=20)
            + s(Time, by=ISUngrammatical, k=20)
            + s(Time, by=ISPresentUngrammatical, k=20)
            + s(Time,Event, bs='fs', m=1, k=20),
            data=subdat, discrete = TRUE, family="scat", 
            AR.start=subdat$start.event, rho=myrho)
```

```
## Warning in gam.side(sm, X, tol = .Machine$double.eps^0.5): model has
## repeated 1-d smooths of same variable.
```

```
#Plot model residuals
qqnorm(resid(Mod2))
qqline(resid(Mod2))
```

#PLOTTING OF GAM model

```
par(mfcol =c(2,2))

#Plotting Past
##Plot past grammatical
plot_smooth(Mod2, view="Time", rm.ranef=TRUE, 
            cond=list(ISPresent=0, ISUngrammatical=0,ISPresentUngrammatical=0),
            col=1, xpd=TRUE, eegAxis = TRUE, ylim = c(2, -2), main="Past: T8")
```

```
## Summary:
##  * Time : numeric predictor; with 30 values ranging from -195.000000 to 1195.000000. 
##  * ISPresent : numeric predictor; set to the value(s): 0. 
##  * ISUngrammatical : numeric predictor; set to the value(s): 0. 
##  * ISPresentUngrammatical : numeric predictor; set to the value(s): 0. 
##  * Event : factor; set to the value(s): Frank01.Past_gram. (Might be canceled as random effect, check below.) 
##  * NOTE : The following random effects columns are canceled: s(Time,Event)
##
```

```
##Plot past ungrammatical
plot_smooth(Mod2, view="Time", rm.ranef=TRUE, 
            cond=list(ISPresent=0, ISUngrammatical=1,ISPresentUngrammatical=0),
            h0=0,add=TRUE, v0=0,col=2)
```

```
## Summary:
##  * Time : numeric predictor; with 30 values ranging from -195.000000 to 1195.000000. 
##  * ISPresent : numeric predictor; set to the value(s): 0. 
##  * ISUngrammatical : numeric predictor; set to the value(s): 1. 
##  * ISPresentUngrammatical : numeric predictor; set to the value(s): 0. 
##  * Event : factor; set to the value(s): Frank01.Past_gram. (Might be canceled as random effect, check below.) 
##  * NOTE : The following random effects columns are canceled: s(Time,Event)
##
```

```
legend_margin('bottomleft', legend=c("grammatical", "ungrammatical"),
       fill=c(alpha(1), alpha(2)), border= c(alpha(1), alpha(2)),
         lwd=1.5, col=c(1,2), seg.len=1, merge=TRUE,
         bty='n')


## Plot differences (i.e. where the grammatical vs ungrammatical lines differ in this case for past)
plot_diff(Mod2, view="Time", cond=list(ISPresent=0, ISPresentUngrammatical=0), 
          comp=list(ISUngrammatical=c(0,1)), 
          rm.ranef = TRUE,
          main="Difference in Past grammaticality")
```

```
## Summary:
##  * Time : numeric predictor; with 100 values ranging from -195.000000 to 1195.000000. 
##  * ISPresent : numeric predictor; set to the value(s): 0. 
##  * ISPresentUngrammatical : numeric predictor; set to the value(s): 0. 
##  * Event : factor; set to the value(s): Frank01.Past_gram. (Might be canceled as random effect, check below.) 
##  * NOTE : The following random effects columns are canceled: s(Time,Event)
##
```

```
## 
## Difference is not significant.
```

```
#Plotting for Present
##Plot present grammatical
plot_smooth(Mod2, view="Time", rm.ranef=TRUE, 
            cond=list(ISPresent=1, ISUngrammatical=0,ISPresentUngrammatical=0),
            col=1, xpd=TRUE, eegAxis = TRUE, ylim = c(2, -2), main="Present: T8")
```

```
## Summary:
##  * Time : numeric predictor; with 30 values ranging from -195.000000 to 1195.000000. 
##  * ISPresent : numeric predictor; set to the value(s): 1. 
##  * ISUngrammatical : numeric predictor; set to the value(s): 0. 
##  * ISPresentUngrammatical : numeric predictor; set to the value(s): 0. 
##  * Event : factor; set to the value(s): Frank01.Past_gram. (Might be canceled as random effect, check below.) 
##  * NOTE : The following random effects columns are canceled: s(Time,Event)
##
```

```
##Plot present ungrammatical
plot_smooth(Mod2, view="Time", rm.ranef=TRUE, 
            cond=list(ISPresent=1, ISUngrammatical=1,ISPresentUngrammatical=1),
            add=TRUE, col=2, xpd=TRUE, main = "Present")
```

```
## Summary:
##  * Time : numeric predictor; with 30 values ranging from -195.000000 to 1195.000000. 
##  * ISPresent : numeric predictor; set to the value(s): 1. 
##  * ISUngrammatical : numeric predictor; set to the value(s): 1. 
##  * ISPresentUngrammatical : numeric predictor; set to the value(s): 1. 
##  * Event : factor; set to the value(s): Frank01.Past_gram. (Might be canceled as random effect, check below.) 
##  * NOTE : The following random effects columns are canceled: s(Time,Event)
##
```

```
## Plot differences (i.e. where the grammatical vs ungrammatical lines differ in this case for present)
plot_diff(Mod2, view="Time", cond=list(ISPresent=1), 
          comp=list(ISUngrammatical=c(0,1), ISPresentUngrammatical=c(0,1)), 
          rm.ranef = TRUE,
          main="Difference in Present grammaticality")
```

```
## Summary:
##  * Time : numeric predictor; with 100 values ranging from -195.000000 to 1195.000000. 
##  * ISPresent : numeric predictor; set to the value(s): 1. 
##  * Event : factor; set to the value(s): Frank01.Past_gram. (Might be canceled as random effect, check below.) 
##  * NOTE : The following random effects columns are canceled: s(Time,Event)
##
```

```
## 
## Difference is not significant.
```

#Subsetting data with electrode: CP5

```
subdat <- droplevels(dat[dat$Channel== "CP5" & dat$Condition %in% c("Past_gram", "Past_ungram", "Present_gram", "Present_ungram"),]) 
head(subdat)
```

```
##      Subject Condition Grammaticality Channel Time       value Tense
## 2381 Frank01 Past_gram    Grammatical     CP5 -195 -0.15870769  Past
## 2382 Frank01 Past_gram    Grammatical     CP5 -185 -0.10407692  Past
## 2383 Frank01 Past_gram    Grammatical     CP5 -175  0.11010577  Past
## 2384 Frank01 Past_gram    Grammatical     CP5 -165  0.36991538  Past
## 2385 Frank01 Past_gram    Grammatical     CP5 -155  0.05132692  Past
## 2386 Frank01 Past_gram    Grammatical     CP5 -145 -0.50370192  Past
##      ISPresent ISUngrammatical ISPresentUngrammatical
## 2381         0               0                      0
## 2382         0               0                      0
## 2383         0               0                      0
## 2384         0               0                      0
## 2385         0               0                      0
## 2386         0               0                      0
```

## Create EVENT (unique combination of Subject and Items)for random effects

```
subdat$Event <- interaction(subdat$Subject, subdat$Condition)

#   Determine starting point for each time series
subdat <- start_event(subdat,column = "Time",event = "Event")

# Make sure variables are factors
subdat$Condition <- as.factor(subdat$Condition) 
subdat$Subject <- as.factor(subdat$Subject)
```

# GAMM ANALYSIS

```
Mod1 <- bam(value ~ s(Time, k=20) 
            + s(Time, by=ISPresent, k=20)
            + s(Time, by=ISUngrammatical, k=20)
            + s(Time, by=ISPresentUngrammatical, k=20)
            + s(Time,Event, bs='fs', m=1, k=20),
            data=subdat, discrete = TRUE, family="scat")
```

```
## Warning in gam.side(sm, X, tol = .Machine$double.eps^0.5): model has
## repeated 1-d smooths of same variable.
```

```
## Calculate rho value to account for autocorrelation in data.
myrho <- start_value_rho(Mod1)

#Fit a Model including rho
Mod2 <- bam(value ~ s(Time, k=20) 
            + s(Time, by=ISPresent, k=20)
            + s(Time, by=ISUngrammatical, k=20)
            + s(Time, by=ISPresentUngrammatical, k=20)
            + s(Time,Event, bs='fs', m=1, k=20),
            data=subdat, discrete = TRUE, family="scat", 
            AR.start=subdat$start.event, rho=myrho)
```

```
## Warning in gam.side(sm, X, tol = .Machine$double.eps^0.5): model has
## repeated 1-d smooths of same variable.
```

```
#Plot model residuals
qqnorm(resid(Mod2))
qqline(resid(Mod2))
```

#PLOTTING OF GAM model

```
par(mfcol =c(2,2))

#Plotting Past
##Plot past grammatical
plot_smooth(Mod2, view="Time", rm.ranef=TRUE, 
            cond=list(ISPresent=0, ISUngrammatical=0,ISPresentUngrammatical=0),
            col=1, xpd=TRUE, eegAxis = TRUE, ylim = c(2, -2), main="Past: CP5")
```

```
## Summary:
##  * Time : numeric predictor; with 30 values ranging from -195.000000 to 1195.000000. 
##  * ISPresent : numeric predictor; set to the value(s): 0. 
##  * ISUngrammatical : numeric predictor; set to the value(s): 0. 
##  * ISPresentUngrammatical : numeric predictor; set to the value(s): 0. 
##  * Event : factor; set to the value(s): Frank01.Past_gram. (Might be canceled as random effect, check below.) 
##  * NOTE : The following random effects columns are canceled: s(Time,Event)
##
```

```
##Plot past ungrammatical
plot_smooth(Mod2, view="Time", rm.ranef=TRUE, 
            cond=list(ISPresent=0, ISUngrammatical=1,ISPresentUngrammatical=0),
            h0=0,add=TRUE, v0=0,col=2)
```

```
## Summary:
##  * Time : numeric predictor; with 30 values ranging from -195.000000 to 1195.000000. 
##  * ISPresent : numeric predictor; set to the value(s): 0. 
##  * ISUngrammatical : numeric predictor; set to the value(s): 1. 
##  * ISPresentUngrammatical : numeric predictor; set to the value(s): 0. 
##  * Event : factor; set to the value(s): Frank01.Past_gram. (Might be canceled as random effect, check below.) 
##  * NOTE : The following random effects columns are canceled: s(Time,Event)
##
```

```
legend_margin('bottomleft', legend=c("grammatical", "ungrammatical"),
       fill=c(alpha(1), alpha(2)), border= c(alpha(1), alpha(2)),
         lwd=1.5, col=c(1,2), seg.len=1, merge=TRUE,
         bty='n')


## Plot differences (i.e. where the grammatical vs ungrammatical lines differ in this case for past)
plot_diff(Mod2, view="Time", cond=list(ISPresent=0, ISPresentUngrammatical=0), 
          comp=list(ISUngrammatical=c(0,1)), 
          rm.ranef = TRUE,
          main="Difference in Past grammaticality")
```

```
## Summary:
##  * Time : numeric predictor; with 100 values ranging from -195.000000 to 1195.000000. 
##  * ISPresent : numeric predictor; set to the value(s): 0. 
##  * ISPresentUngrammatical : numeric predictor; set to the value(s): 0. 
##  * Event : factor; set to the value(s): Frank01.Past_gram. (Might be canceled as random effect, check below.) 
##  * NOTE : The following random effects columns are canceled: s(Time,Event)
##
```

```
## 
## Time window(s) of significant difference(s):
##  886.111111 - 1152.878788
```

```
#Plotting for Present
##Plot present grammatical
plot_smooth(Mod2, view="Time", rm.ranef=TRUE, 
            cond=list(ISPresent=1, ISUngrammatical=0,ISPresentUngrammatical=0),
            col=1, xpd=TRUE, eegAxis = TRUE, ylim = c(2, -2), main="Present: CP5")
```

```
## Summary:
##  * Time : numeric predictor; with 30 values ranging from -195.000000 to 1195.000000. 
##  * ISPresent : numeric predictor; set to the value(s): 1. 
##  * ISUngrammatical : numeric predictor; set to the value(s): 0. 
##  * ISPresentUngrammatical : numeric predictor; set to the value(s): 0. 
##  * Event : factor; set to the value(s): Frank01.Past_gram. (Might be canceled as random effect, check below.) 
##  * NOTE : The following random effects columns are canceled: s(Time,Event)
##
```

```
##Plot present ungrammatical
plot_smooth(Mod2, view="Time", rm.ranef=TRUE, 
            cond=list(ISPresent=1, ISUngrammatical=1,ISPresentUngrammatical=1),
            add=TRUE, col=2, xpd=TRUE, main = "Present")
```

```
## Summary:
##  * Time : numeric predictor; with 30 values ranging from -195.000000 to 1195.000000. 
##  * ISPresent : numeric predictor; set to the value(s): 1. 
##  * ISUngrammatical : numeric predictor; set to the value(s): 1. 
##  * ISPresentUngrammatical : numeric predictor; set to the value(s): 1. 
##  * Event : factor; set to the value(s): Frank01.Past_gram. (Might be canceled as random effect, check below.) 
##  * NOTE : The following random effects columns are canceled: s(Time,Event)
##
```

```
## Plot differences (i.e. where the grammatical vs ungrammatical lines differ in this case for present)
plot_diff(Mod2, view="Time", cond=list(ISPresent=1), 
          comp=list(ISUngrammatical=c(0,1), ISPresentUngrammatical=c(0,1)), 
          rm.ranef = TRUE,
          main="Difference in Present grammaticality")
```

```
## Summary:
##  * Time : numeric predictor; with 100 values ranging from -195.000000 to 1195.000000. 
##  * ISPresent : numeric predictor; set to the value(s): 1. 
##  * Event : factor; set to the value(s): Frank01.Past_gram. (Might be canceled as random effect, check below.) 
##  * NOTE : The following random effects columns are canceled: s(Time,Event)
##
```

```
## 
## Difference is not significant.
```

#Subsetting data with electrode: CP1

```
subdat <- droplevels(dat[dat$Channel== "CP1" & dat$Condition %in% c("Past_gram", "Past_ungram", "Present_gram", "Present_ungram"),]) 
head(subdat)
```

```
##      Subject Condition Grammaticality Channel Time      value Tense
## 2521 Frank01 Past_gram    Grammatical     CP1 -195 -0.3449154  Past
## 2522 Frank01 Past_gram    Grammatical     CP1 -185  0.3341410  Past
## 2523 Frank01 Past_gram    Grammatical     CP1 -175  0.8001923  Past
## 2524 Frank01 Past_gram    Grammatical     CP1 -165  0.5819308  Past
## 2525 Frank01 Past_gram    Grammatical     CP1 -155  0.1760769  Past
## 2526 Frank01 Past_gram    Grammatical     CP1 -145 -0.2139038  Past
##      ISPresent ISUngrammatical ISPresentUngrammatical
## 2521         0               0                      0
## 2522         0               0                      0
## 2523         0               0                      0
## 2524         0               0                      0
## 2525         0               0                      0
## 2526         0               0                      0
```

## Create EVENT (unique combination of Subject and Items)for random effects

```
subdat$Event <- interaction(subdat$Subject, subdat$Condition)

#   Determine starting point for each time series
subdat <- start_event(subdat,column = "Time",event = "Event")

# Make sure variables are factors
subdat$Condition <- as.factor(subdat$Condition) 
subdat$Subject <- as.factor(subdat$Subject)
```

# GAMM ANALYSIS

```
Mod1 <- bam(value ~ s(Time, k=20) 
            + s(Time, by=ISPresent, k=20)
            + s(Time, by=ISUngrammatical, k=20)
            + s(Time, by=ISPresentUngrammatical, k=20)
            + s(Time,Event, bs='fs', m=1, k=20),
            data=subdat, discrete = TRUE, family="scat")
```

```
## Warning in gam.side(sm, X, tol = .Machine$double.eps^0.5): model has
## repeated 1-d smooths of same variable.
```

```
## Calculate rho value to account for autocorrelation in data.
myrho <- start_value_rho(Mod1)

#Fit a Model including rho
Mod2 <- bam(value ~ s(Time, k=20) 
            + s(Time, by=ISPresent, k=20)
            + s(Time, by=ISUngrammatical, k=20)
            + s(Time, by=ISPresentUngrammatical, k=20)
            + s(Time,Event, bs='fs', m=1, k=20),
            data=subdat, discrete = TRUE, family="scat", 
            AR.start=subdat$start.event, rho=myrho)
```

```
## Warning in gam.side(sm, X, tol = .Machine$double.eps^0.5): model has
## repeated 1-d smooths of same variable.
```

```
#Plot model residuals
qqnorm(resid(Mod2))
qqline(resid(Mod2))
```

#PLOTTING OF GAM model

```
par(mfcol =c(2,2))

#Plotting Past
##Plot past grammatical
plot_smooth(Mod2, view="Time", rm.ranef=TRUE, 
            cond=list(ISPresent=0, ISUngrammatical=0,ISPresentUngrammatical=0),
            col=1, xpd=TRUE, eegAxis = TRUE, ylim = c(2, -2), main="Past: CP1")
```

```
## Summary:
##  * Time : numeric predictor; with 30 values ranging from -195.000000 to 1195.000000. 
##  * ISPresent : numeric predictor; set to the value(s): 0. 
##  * ISUngrammatical : numeric predictor; set to the value(s): 0. 
##  * ISPresentUngrammatical : numeric predictor; set to the value(s): 0. 
##  * Event : factor; set to the value(s): Frank01.Past_gram. (Might be canceled as random effect, check below.) 
##  * NOTE : The following random effects columns are canceled: s(Time,Event)
##
```

```
##Plot past ungrammatical
plot_smooth(Mod2, view="Time", rm.ranef=TRUE, 
            cond=list(ISPresent=0, ISUngrammatical=1,ISPresentUngrammatical=0),
            h0=0,add=TRUE, v0=0,col=2)
```

```
## Summary:
##  * Time : numeric predictor; with 30 values ranging from -195.000000 to 1195.000000. 
##  * ISPresent : numeric predictor; set to the value(s): 0. 
##  * ISUngrammatical : numeric predictor; set to the value(s): 1. 
##  * ISPresentUngrammatical : numeric predictor; set to the value(s): 0. 
##  * Event : factor; set to the value(s): Frank01.Past_gram. (Might be canceled as random effect, check below.) 
##  * NOTE : The following random effects columns are canceled: s(Time,Event)
##
```

```
legend_margin('bottomleft', legend=c("grammatical", "ungrammatical"),
       fill=c(alpha(1), alpha(2)), border= c(alpha(1), alpha(2)),
         lwd=1.5, col=c(1,2), seg.len=1, merge=TRUE,
         bty='n')


## Plot differences (i.e. where the grammatical vs ungrammatical lines differ in this case for past)
plot_diff(Mod2, view="Time", cond=list(ISPresent=0, ISPresentUngrammatical=0), 
          comp=list(ISUngrammatical=c(0,1)), 
          rm.ranef = TRUE,
          main="Difference in Past grammaticality")
```

```
## Summary:
##  * Time : numeric predictor; with 100 values ranging from -195.000000 to 1195.000000. 
##  * ISPresent : numeric predictor; set to the value(s): 0. 
##  * ISPresentUngrammatical : numeric predictor; set to the value(s): 0. 
##  * Event : factor; set to the value(s): Frank01.Past_gram. (Might be canceled as random effect, check below.) 
##  * NOTE : The following random effects columns are canceled: s(Time,Event)
##
```

```
## 
## Time window(s) of significant difference(s):
##  450.858586 - 1195.000000
```

```
#Plotting for Present
##Plot present grammatical
plot_smooth(Mod2, view="Time", rm.ranef=TRUE, 
            cond=list(ISPresent=1, ISUngrammatical=0,ISPresentUngrammatical=0),
            col=1, xpd=TRUE, eegAxis = TRUE, ylim = c(2, -2), main="Present: CP1")
```

```
## Summary:
##  * Time : numeric predictor; with 30 values ranging from -195.000000 to 1195.000000. 
##  * ISPresent : numeric predictor; set to the value(s): 1. 
##  * ISUngrammatical : numeric predictor; set to the value(s): 0. 
##  * ISPresentUngrammatical : numeric predictor; set to the value(s): 0. 
##  * Event : factor; set to the value(s): Frank01.Past_gram. (Might be canceled as random effect, check below.) 
##  * NOTE : The following random effects columns are canceled: s(Time,Event)
##
```

```
##Plot present ungrammatical
plot_smooth(Mod2, view="Time", rm.ranef=TRUE, 
            cond=list(ISPresent=1, ISUngrammatical=1,ISPresentUngrammatical=1),
            add=TRUE, col=2, xpd=TRUE, main = "Present")
```

```
## Summary:
##  * Time : numeric predictor; with 30 values ranging from -195.000000 to 1195.000000. 
##  * ISPresent : numeric predictor; set to the value(s): 1. 
##  * ISUngrammatical : numeric predictor; set to the value(s): 1. 
##  * ISPresentUngrammatical : numeric predictor; set to the value(s): 1. 
##  * Event : factor; set to the value(s): Frank01.Past_gram. (Might be canceled as random effect, check below.) 
##  * NOTE : The following random effects columns are canceled: s(Time,Event)
##
```

```
## Plot differences (i.e. where the grammatical vs ungrammatical lines differ in this case for present)
plot_diff(Mod2, view="Time", cond=list(ISPresent=1), 
          comp=list(ISUngrammatical=c(0,1), ISPresentUngrammatical=c(0,1)), 
          rm.ranef = TRUE,
          main="Difference in Present grammaticality")
```

```
## Summary:
##  * Time : numeric predictor; with 100 values ranging from -195.000000 to 1195.000000. 
##  * ISPresent : numeric predictor; set to the value(s): 1. 
##  * Event : factor; set to the value(s): Frank01.Past_gram. (Might be canceled as random effect, check below.) 
##  * NOTE : The following random effects columns are canceled: s(Time,Event)
##
```

```
## 
## Difference is not significant.
```

#Subsetting data with electrode: CP2

```
subdat <- droplevels(dat[dat$Channel== "CP2" & dat$Condition %in% c("Past_gram", "Past_ungram", "Present_gram", "Present_ungram"),]) 
head(subdat)
```

```
##      Subject Condition Grammaticality Channel Time       value Tense
## 2661 Frank01 Past_gram    Grammatical     CP2 -195 -0.40298462  Past
## 2662 Frank01 Past_gram    Grammatical     CP2 -185  0.04812821  Past
## 2663 Frank01 Past_gram    Grammatical     CP2 -175  0.21527885  Past
## 2664 Frank01 Past_gram    Grammatical     CP2 -165 -0.23216154  Past
## 2665 Frank01 Past_gram    Grammatical     CP2 -155 -0.51369872  Past
## 2666 Frank01 Past_gram    Grammatical     CP2 -145 -0.66396154  Past
##      ISPresent ISUngrammatical ISPresentUngrammatical
## 2661         0               0                      0
## 2662         0               0                      0
## 2663         0               0                      0
## 2664         0               0                      0
## 2665         0               0                      0
## 2666         0               0                      0
```

## Create EVENT (unique combination of Subject and Items)for random effects

```
subdat$Event <- interaction(subdat$Subject, subdat$Condition)

#   Determine starting point for each time series
subdat <- start_event(subdat,column = "Time",event = "Event")

# Make sure variables are factors
subdat$Condition <- as.factor(subdat$Condition) 
subdat$Subject <- as.factor(subdat$Subject)
```

# GAMM ANALYSIS

```
Mod1 <- bam(value ~ s(Time, k=20) 
            + s(Time, by=ISPresent, k=20)
            + s(Time, by=ISUngrammatical, k=20)
            + s(Time, by=ISPresentUngrammatical, k=20)
            + s(Time,Event, bs='fs', m=1, k=20),
            data=subdat, discrete = TRUE, family="scat")
```

```
## Warning in gam.side(sm, X, tol = .Machine$double.eps^0.5): model has
## repeated 1-d smooths of same variable.
```

```
## Calculate rho value to account for autocorrelation in data.
myrho <- start_value_rho(Mod1)

#Fit a Model including rho
Mod2 <- bam(value ~ s(Time, k=20) 
            + s(Time, by=ISPresent, k=20)
            + s(Time, by=ISUngrammatical, k=20)
            + s(Time, by=ISPresentUngrammatical, k=20)
            + s(Time,Event, bs='fs', m=1, k=20),
            data=subdat, discrete = TRUE, family="scat", 
            AR.start=subdat$start.event, rho=myrho)
```

```
## Warning in gam.side(sm, X, tol = .Machine$double.eps^0.5): model has
## repeated 1-d smooths of same variable.
```

```
#Plot model residuals
qqnorm(resid(Mod2))
qqline(resid(Mod2))
```

#PLOTTING OF GAM model

```
par(mfcol =c(2,2))

#Plotting Past
##Plot past grammatical
plot_smooth(Mod2, view="Time", rm.ranef=TRUE, 
            cond=list(ISPresent=0, ISUngrammatical=0,ISPresentUngrammatical=0),
            col=1, xpd=TRUE, eegAxis = TRUE, ylim = c(2, -2), main="Past: CP2")
```

```
## Summary:
##  * Time : numeric predictor; with 30 values ranging from -195.000000 to 1195.000000. 
##  * ISPresent : numeric predictor; set to the value(s): 0. 
##  * ISUngrammatical : numeric predictor; set to the value(s): 0. 
##  * ISPresentUngrammatical : numeric predictor; set to the value(s): 0. 
##  * Event : factor; set to the value(s): Frank01.Past_gram. (Might be canceled as random effect, check below.) 
##  * NOTE : The following random effects columns are canceled: s(Time,Event)
##
```

```
##Plot past ungrammatical
plot_smooth(Mod2, view="Time", rm.ranef=TRUE, 
            cond=list(ISPresent=0, ISUngrammatical=1,ISPresentUngrammatical=0),
            h0=0,add=TRUE, v0=0,col=2)
```

```
## Summary:
##  * Time : numeric predictor; with 30 values ranging from -195.000000 to 1195.000000. 
##  * ISPresent : numeric predictor; set to the value(s): 0. 
##  * ISUngrammatical : numeric predictor; set to the value(s): 1. 
##  * ISPresentUngrammatical : numeric predictor; set to the value(s): 0. 
##  * Event : factor; set to the value(s): Frank01.Past_gram. (Might be canceled as random effect, check below.) 
##  * NOTE : The following random effects columns are canceled: s(Time,Event)
##
```

```
legend_margin('bottomleft', legend=c("grammatical", "ungrammatical"),
       fill=c(alpha(1), alpha(2)), border= c(alpha(1), alpha(2)),
         lwd=1.5, col=c(1,2), seg.len=1, merge=TRUE,
         bty='n')


## Plot differences (i.e. where the grammatical vs ungrammatical lines differ in this case for past)
plot_diff(Mod2, view="Time", cond=list(ISPresent=0, ISPresentUngrammatical=0), 
          comp=list(ISUngrammatical=c(0,1)), 
          rm.ranef = TRUE,
          main="Difference in Past grammaticality")
```

```
## Summary:
##  * Time : numeric predictor; with 100 values ranging from -195.000000 to 1195.000000. 
##  * ISPresent : numeric predictor; set to the value(s): 0. 
##  * ISPresentUngrammatical : numeric predictor; set to the value(s): 0. 
##  * Event : factor; set to the value(s): Frank01.Past_gram. (Might be canceled as random effect, check below.) 
##  * NOTE : The following random effects columns are canceled: s(Time,Event)
##
```

```
## 
## Time window(s) of significant difference(s):
##  464.898990 - 1195.000000
```

```
#Plotting for Present
##Plot present grammatical
plot_smooth(Mod2, view="Time", rm.ranef=TRUE, 
            cond=list(ISPresent=1, ISUngrammatical=0,ISPresentUngrammatical=0),
            col=1, xpd=TRUE, eegAxis = TRUE, ylim = c(2, -2), main="Present: CP2")
```

```
## Summary:
##  * Time : numeric predictor; with 30 values ranging from -195.000000 to 1195.000000. 
##  * ISPresent : numeric predictor; set to the value(s): 1. 
##  * ISUngrammatical : numeric predictor; set to the value(s): 0. 
##  * ISPresentUngrammatical : numeric predictor; set to the value(s): 0. 
##  * Event : factor; set to the value(s): Frank01.Past_gram. (Might be canceled as random effect, check below.) 
##  * NOTE : The following random effects columns are canceled: s(Time,Event)
##
```

```
##Plot present ungrammatical
plot_smooth(Mod2, view="Time", rm.ranef=TRUE, 
            cond=list(ISPresent=1, ISUngrammatical=1,ISPresentUngrammatical=1),
            add=TRUE, col=2, xpd=TRUE, main = "Present")
```

```
## Summary:
##  * Time : numeric predictor; with 30 values ranging from -195.000000 to 1195.000000. 
##  * ISPresent : numeric predictor; set to the value(s): 1. 
##  * ISUngrammatical : numeric predictor; set to the value(s): 1. 
##  * ISPresentUngrammatical : numeric predictor; set to the value(s): 1. 
##  * Event : factor; set to the value(s): Frank01.Past_gram. (Might be canceled as random effect, check below.) 
##  * NOTE : The following random effects columns are canceled: s(Time,Event)
##
```

```
## Plot differences (i.e. where the grammatical vs ungrammatical lines differ in this case for present)
plot_diff(Mod2, view="Time", cond=list(ISPresent=1), 
          comp=list(ISUngrammatical=c(0,1), ISPresentUngrammatical=c(0,1)), 
          rm.ranef = TRUE,
          main="Difference in Present grammaticality")
```

```
## Summary:
##  * Time : numeric predictor; with 100 values ranging from -195.000000 to 1195.000000. 
##  * ISPresent : numeric predictor; set to the value(s): 1. 
##  * Event : factor; set to the value(s): Frank01.Past_gram. (Might be canceled as random effect, check below.) 
##  * NOTE : The following random effects columns are canceled: s(Time,Event)
##
```

```
## 
## Difference is not significant.
```

#Subsetting data with electrode: CP6

```
subdat <- droplevels(dat[dat$Channel== "CP6" & dat$Condition %in% c("Past_gram", "Past_ungram", "Present_gram", "Present_ungram"),]) 
head(subdat)
```

```
##      Subject Condition Grammaticality Channel Time        value Tense
## 2801 Frank01 Past_gram    Grammatical     CP6 -195  0.265061538  Past
## 2802 Frank01 Past_gram    Grammatical     CP6 -185  1.311974359  Past
## 2803 Frank01 Past_gram    Grammatical     CP6 -175  1.538000000  Past
## 2804 Frank01 Past_gram    Grammatical     CP6 -165  0.865823077  Past
## 2805 Frank01 Past_gram    Grammatical     CP6 -155 -0.008897436  Past
## 2806 Frank01 Past_gram    Grammatical     CP6 -145 -0.850663462  Past
##      ISPresent ISUngrammatical ISPresentUngrammatical
## 2801         0               0                      0
## 2802         0               0                      0
## 2803         0               0                      0
## 2804         0               0                      0
## 2805         0               0                      0
## 2806         0               0                      0
```

## Create EVENT (unique combination of Subject and Items)for random effects

```
subdat$Event <- interaction(subdat$Subject, subdat$Condition)

#   Determine starting point for each time series
subdat <- start_event(subdat,column = "Time",event = "Event")

# Make sure variables are factors
subdat$Condition <- as.factor(subdat$Condition) 
subdat$Subject <- as.factor(subdat$Subject)
```

# GAMM ANALYSIS

```
Mod1 <- bam(value ~ s(Time, k=20) 
            + s(Time, by=ISPresent, k=20)
            + s(Time, by=ISUngrammatical, k=20)
            + s(Time, by=ISPresentUngrammatical, k=20)
            + s(Time,Event, bs='fs', m=1, k=20),
            data=subdat, discrete = TRUE, family="scat")
```

```
## Warning in gam.side(sm, X, tol = .Machine$double.eps^0.5): model has
## repeated 1-d smooths of same variable.
```

```
## Calculate rho value to account for autocorrelation in data.
myrho <- start_value_rho(Mod1)

#Fit a Model including rho
Mod2 <- bam(value ~ s(Time, k=20) 
            + s(Time, by=ISPresent, k=20)
            + s(Time, by=ISUngrammatical, k=20)
            + s(Time, by=ISPresentUngrammatical, k=20)
            + s(Time,Event, bs='fs', m=1, k=20),
            data=subdat, discrete = TRUE, family="scat", 
            AR.start=subdat$start.event, rho=myrho)
```

```
## Warning in gam.side(sm, X, tol = .Machine$double.eps^0.5): model has
## repeated 1-d smooths of same variable.
```

```
#Plot model residuals
qqnorm(resid(Mod2))
qqline(resid(Mod2))
```

#PLOTTING OF GAM model

```
par(mfcol =c(2,2))

#Plotting Past
##Plot past grammatical
plot_smooth(Mod2, view="Time", rm.ranef=TRUE, 
            cond=list(ISPresent=0, ISUngrammatical=0,ISPresentUngrammatical=0),
            col=1, xpd=TRUE, eegAxis = TRUE, ylim = c(2, -2), main="Past: CP6")
```

```
## Summary:
##  * Time : numeric predictor; with 30 values ranging from -195.000000 to 1195.000000. 
##  * ISPresent : numeric predictor; set to the value(s): 0. 
##  * ISUngrammatical : numeric predictor; set to the value(s): 0. 
##  * ISPresentUngrammatical : numeric predictor; set to the value(s): 0. 
##  * Event : factor; set to the value(s): Frank01.Past_gram. (Might be canceled as random effect, check below.) 
##  * NOTE : The following random effects columns are canceled: s(Time,Event)
##
```

```
##Plot past ungrammatical
plot_smooth(Mod2, view="Time", rm.ranef=TRUE, 
            cond=list(ISPresent=0, ISUngrammatical=1,ISPresentUngrammatical=0),
            h0=0,add=TRUE, v0=0,col=2)
```

```
## Summary:
##  * Time : numeric predictor; with 30 values ranging from -195.000000 to 1195.000000. 
##  * ISPresent : numeric predictor; set to the value(s): 0. 
##  * ISUngrammatical : numeric predictor; set to the value(s): 1. 
##  * ISPresentUngrammatical : numeric predictor; set to the value(s): 0. 
##  * Event : factor; set to the value(s): Frank01.Past_gram. (Might be canceled as random effect, check below.) 
##  * NOTE : The following random effects columns are canceled: s(Time,Event)
##
```

```
legend_margin('bottomleft', legend=c("grammatical", "ungrammatical"),
       fill=c(alpha(1), alpha(2)), border= c(alpha(1), alpha(2)),
         lwd=1.5, col=c(1,2), seg.len=1, merge=TRUE,
         bty='n')


## Plot differences (i.e. where the grammatical vs ungrammatical lines differ in this case for past)
plot_diff(Mod2, view="Time", cond=list(ISPresent=0, ISPresentUngrammatical=0), 
          comp=list(ISUngrammatical=c(0,1)), 
          rm.ranef = TRUE,
          main="Difference in Past grammaticality")
```

```
## Summary:
##  * Time : numeric predictor; with 100 values ranging from -195.000000 to 1195.000000. 
##  * ISPresent : numeric predictor; set to the value(s): 0. 
##  * ISPresentUngrammatical : numeric predictor; set to the value(s): 0. 
##  * Event : factor; set to the value(s): Frank01.Past_gram. (Might be canceled as random effect, check below.) 
##  * NOTE : The following random effects columns are canceled: s(Time,Event)
##
```

```
## 
## Difference is not significant.
```

```
#Plotting for Present
##Plot present grammatical
plot_smooth(Mod2, view="Time", rm.ranef=TRUE, 
            cond=list(ISPresent=1, ISUngrammatical=0,ISPresentUngrammatical=0),
            col=1, xpd=TRUE, eegAxis = TRUE, ylim = c(2, -2), main="Present: CP6")
```

```
## Summary:
##  * Time : numeric predictor; with 30 values ranging from -195.000000 to 1195.000000. 
##  * ISPresent : numeric predictor; set to the value(s): 1. 
##  * ISUngrammatical : numeric predictor; set to the value(s): 0. 
##  * ISPresentUngrammatical : numeric predictor; set to the value(s): 0. 
##  * Event : factor; set to the value(s): Frank01.Past_gram. (Might be canceled as random effect, check below.) 
##  * NOTE : The following random effects columns are canceled: s(Time,Event)
##
```

```
##Plot present ungrammatical
plot_smooth(Mod2, view="Time", rm.ranef=TRUE, 
            cond=list(ISPresent=1, ISUngrammatical=1,ISPresentUngrammatical=1),
            add=TRUE, col=2, xpd=TRUE, main = "Present")
```

```
## Summary:
##  * Time : numeric predictor; with 30 values ranging from -195.000000 to 1195.000000. 
##  * ISPresent : numeric predictor; set to the value(s): 1. 
##  * ISUngrammatical : numeric predictor; set to the value(s): 1. 
##  * ISPresentUngrammatical : numeric predictor; set to the value(s): 1. 
##  * Event : factor; set to the value(s): Frank01.Past_gram. (Might be canceled as random effect, check below.) 
##  * NOTE : The following random effects columns are canceled: s(Time,Event)
##
```

```
## Plot differences (i.e. where the grammatical vs ungrammatical lines differ in this case for present)
plot_diff(Mod2, view="Time", cond=list(ISPresent=1), 
          comp=list(ISUngrammatical=c(0,1), ISPresentUngrammatical=c(0,1)), 
          rm.ranef = TRUE,
          main="Difference in Present grammaticality")
```

```
## Summary:
##  * Time : numeric predictor; with 100 values ranging from -195.000000 to 1195.000000. 
##  * ISPresent : numeric predictor; set to the value(s): 1. 
##  * Event : factor; set to the value(s): Frank01.Past_gram. (Might be canceled as random effect, check below.) 
##  * NOTE : The following random effects columns are canceled: s(Time,Event)
##
```

```
## 
## Difference is not significant.
```

#Subsetting data with electrode: P7

```
subdat <- droplevels(dat[dat$Channel== "P7" & dat$Condition %in% c("Past_gram", "Past_ungram", "Present_gram", "Present_ungram"),]) 
head(subdat)
```

```
##      Subject Condition Grammaticality Channel Time       value Tense
## 2941 Frank01 Past_gram    Grammatical      P7 -195  0.53855385  Past
## 2942 Frank01 Past_gram    Grammatical      P7 -185 -0.02655128  Past
## 2943 Frank01 Past_gram    Grammatical      P7 -175 -0.43590385  Past
## 2944 Frank01 Past_gram    Grammatical      P7 -165  0.36793077  Past
## 2945 Frank01 Past_gram    Grammatical      P7 -155  0.38423077  Past
## 2946 Frank01 Past_gram    Grammatical      P7 -145 -0.60423077  Past
##      ISPresent ISUngrammatical ISPresentUngrammatical
## 2941         0               0                      0
## 2942         0               0                      0
## 2943         0               0                      0
## 2944         0               0                      0
## 2945         0               0                      0
## 2946         0               0                      0
```

## Create EVENT (unique combination of Subject and Items)for random effects

```
subdat$Event <- interaction(subdat$Subject, subdat$Condition)

#   Determine starting point for each time series
subdat <- start_event(subdat,column = "Time",event = "Event")

# Make sure variables are factors
subdat$Condition <- as.factor(subdat$Condition) 
subdat$Subject <- as.factor(subdat$Subject)
```

# GAMM ANALYSIS

```
Mod1 <- bam(value ~ s(Time, k=20) 
            + s(Time, by=ISPresent, k=20)
            + s(Time, by=ISUngrammatical, k=20)
            + s(Time, by=ISPresentUngrammatical, k=20)
            + s(Time,Event, bs='fs', m=1, k=20),
            data=subdat, discrete = TRUE, family="scat")
```

```
## Warning in gam.side(sm, X, tol = .Machine$double.eps^0.5): model has
## repeated 1-d smooths of same variable.
```

```
## Calculate rho value to account for autocorrelation in data.
myrho <- start_value_rho(Mod1)

#Fit a Model including rho
Mod2 <- bam(value ~ s(Time, k=20) 
            + s(Time, by=ISPresent, k=20)
            + s(Time, by=ISUngrammatical, k=20)
            + s(Time, by=ISPresentUngrammatical, k=20)
            + s(Time,Event, bs='fs', m=1, k=20),
            data=subdat, discrete = TRUE, family="scat", 
            AR.start=subdat$start.event, rho=myrho)
```

```
## Warning in gam.side(sm, X, tol = .Machine$double.eps^0.5): model has
## repeated 1-d smooths of same variable.
```

```
#Plot model residuals
qqnorm(resid(Mod2))
qqline(resid(Mod2))
```

#PLOTTING OF GAM model

```
par(mfcol =c(2,2))

#Plotting Past
##Plot past grammatical
plot_smooth(Mod2, view="Time", rm.ranef=TRUE, 
            cond=list(ISPresent=0, ISUngrammatical=0,ISPresentUngrammatical=0),
            col=1, xpd=TRUE, eegAxis = TRUE, ylim = c(2, -2), main="Past: P7")
```

```
## Summary:
##  * Time : numeric predictor; with 30 values ranging from -195.000000 to 1195.000000. 
##  * ISPresent : numeric predictor; set to the value(s): 0. 
##  * ISUngrammatical : numeric predictor; set to the value(s): 0. 
##  * ISPresentUngrammatical : numeric predictor; set to the value(s): 0. 
##  * Event : factor; set to the value(s): Frank01.Past_gram. (Might be canceled as random effect, check below.) 
##  * NOTE : The following random effects columns are canceled: s(Time,Event)
##
```

```
##Plot past ungrammatical
plot_smooth(Mod2, view="Time", rm.ranef=TRUE, 
            cond=list(ISPresent=0, ISUngrammatical=1,ISPresentUngrammatical=0),
            h0=0,add=TRUE, v0=0,col=2)
```

```
## Summary:
##  * Time : numeric predictor; with 30 values ranging from -195.000000 to 1195.000000. 
##  * ISPresent : numeric predictor; set to the value(s): 0. 
##  * ISUngrammatical : numeric predictor; set to the value(s): 1. 
##  * ISPresentUngrammatical : numeric predictor; set to the value(s): 0. 
##  * Event : factor; set to the value(s): Frank01.Past_gram. (Might be canceled as random effect, check below.) 
##  * NOTE : The following random effects columns are canceled: s(Time,Event)
##
```

```
legend_margin('bottomleft', legend=c("grammatical", "ungrammatical"),
       fill=c(alpha(1), alpha(2)), border= c(alpha(1), alpha(2)),
         lwd=1.5, col=c(1,2), seg.len=1, merge=TRUE,
         bty='n')


## Plot differences (i.e. where the grammatical vs ungrammatical lines differ in this case for past)
plot_diff(Mod2, view="Time", cond=list(ISPresent=0, ISPresentUngrammatical=0), 
          comp=list(ISUngrammatical=c(0,1)), 
          rm.ranef = TRUE,
          main="Difference in Past grammaticality")
```

```
## Summary:
##  * Time : numeric predictor; with 100 values ranging from -195.000000 to 1195.000000. 
##  * ISPresent : numeric predictor; set to the value(s): 0. 
##  * ISPresentUngrammatical : numeric predictor; set to the value(s): 0. 
##  * Event : factor; set to the value(s): Frank01.Past_gram. (Might be canceled as random effect, check below.) 
##  * NOTE : The following random effects columns are canceled: s(Time,Event)
##
```

```
## 
## Time window(s) of significant difference(s):
##  661.464646 - 1195.000000
```

```
#Plotting for Present
##Plot present grammatical
plot_smooth(Mod2, view="Time", rm.ranef=TRUE, 
            cond=list(ISPresent=1, ISUngrammatical=0,ISPresentUngrammatical=0),
            col=1, xpd=TRUE, eegAxis = TRUE, ylim = c(2, -2), main="Present: P7")
```

```
## Summary:
##  * Time : numeric predictor; with 30 values ranging from -195.000000 to 1195.000000. 
##  * ISPresent : numeric predictor; set to the value(s): 1. 
##  * ISUngrammatical : numeric predictor; set to the value(s): 0. 
##  * ISPresentUngrammatical : numeric predictor; set to the value(s): 0. 
##  * Event : factor; set to the value(s): Frank01.Past_gram. (Might be canceled as random effect, check below.) 
##  * NOTE : The following random effects columns are canceled: s(Time,Event)
##
```

```
##Plot present ungrammatical
plot_smooth(Mod2, view="Time", rm.ranef=TRUE, 
            cond=list(ISPresent=1, ISUngrammatical=1,ISPresentUngrammatical=1),
            add=TRUE, col=2, xpd=TRUE, main = "Present")
```

```
## Summary:
##  * Time : numeric predictor; with 30 values ranging from -195.000000 to 1195.000000. 
##  * ISPresent : numeric predictor; set to the value(s): 1. 
##  * ISUngrammatical : numeric predictor; set to the value(s): 1. 
##  * ISPresentUngrammatical : numeric predictor; set to the value(s): 1. 
##  * Event : factor; set to the value(s): Frank01.Past_gram. (Might be canceled as random effect, check below.) 
##  * NOTE : The following random effects columns are canceled: s(Time,Event)
##
```

```
## Plot differences (i.e. where the grammatical vs ungrammatical lines differ in this case for present)
plot_diff(Mod2, view="Time", cond=list(ISPresent=1), 
          comp=list(ISUngrammatical=c(0,1), ISPresentUngrammatical=c(0,1)), 
          rm.ranef = TRUE,
          main="Difference in Present grammaticality")
```

```
## Summary:
##  * Time : numeric predictor; with 100 values ranging from -195.000000 to 1195.000000. 
##  * ISPresent : numeric predictor; set to the value(s): 1. 
##  * Event : factor; set to the value(s): Frank01.Past_gram. (Might be canceled as random effect, check below.) 
##  * NOTE : The following random effects columns are canceled: s(Time,Event)
##
```

```
## 
## Difference is not significant.
```

#Subsetting data with electrode: P3

```
subdat <- droplevels(dat[dat$Channel== "P3" & dat$Condition %in% c("Past_gram", "Past_ungram", "Present_gram", "Present_ungram"),]) 
head(subdat)
```

```
##      Subject Condition Grammaticality Channel Time       value Tense
## 3081 Frank01 Past_gram    Grammatical      P3 -195 -0.04900000  Past
## 3082 Frank01 Past_gram    Grammatical      P3 -185  0.14799359  Past
## 3083 Frank01 Past_gram    Grammatical      P3 -175  0.12288462  Past
## 3084 Frank01 Past_gram    Grammatical      P3 -165  0.16774615  Past
## 3085 Frank01 Past_gram    Grammatical      P3 -155  0.05007051  Past
## 3086 Frank01 Past_gram    Grammatical      P3 -145 -0.34439423  Past
##      ISPresent ISUngrammatical ISPresentUngrammatical
## 3081         0               0                      0
## 3082         0               0                      0
## 3083         0               0                      0
## 3084         0               0                      0
## 3085         0               0                      0
## 3086         0               0                      0
```

## Create EVENT (unique combination of Subject and Items)for random effects

```
subdat$Event <- interaction(subdat$Subject, subdat$Condition)

#   Determine starting point for each time series
subdat <- start_event(subdat,column = "Time",event = "Event")

# Make sure variables are factors
subdat$Condition <- as.factor(subdat$Condition) 
subdat$Subject <- as.factor(subdat$Subject)
```

# GAMM ANALYSIS

```
Mod1 <- bam(value ~ s(Time, k=20) 
            + s(Time, by=ISPresent, k=20)
            + s(Time, by=ISUngrammatical, k=20)
            + s(Time, by=ISPresentUngrammatical, k=20)
            + s(Time,Event, bs='fs', m=1, k=20),
            data=subdat, discrete = TRUE, family="scat")
```

```
## Warning in gam.side(sm, X, tol = .Machine$double.eps^0.5): model has
## repeated 1-d smooths of same variable.
```

```
## Calculate rho value to account for autocorrelation in data.
myrho <- start_value_rho(Mod1)

#Fit a Model including rho
Mod2 <- bam(value ~ s(Time, k=20) 
            + s(Time, by=ISPresent, k=20)
            + s(Time, by=ISUngrammatical, k=20)
            + s(Time, by=ISPresentUngrammatical, k=20)
            + s(Time,Event, bs='fs', m=1, k=20),
            data=subdat, discrete = TRUE, family="scat", 
            AR.start=subdat$start.event, rho=myrho)
```

```
## Warning in gam.side(sm, X, tol = .Machine$double.eps^0.5): model has
## repeated 1-d smooths of same variable.
```

```
#Plot model residuals
qqnorm(resid(Mod2))
qqline(resid(Mod2))
```

#PLOTTING OF GAM model

```
par(mfcol =c(2,2))

#Plotting Past
##Plot past grammatical
plot_smooth(Mod2, view="Time", rm.ranef=TRUE, 
            cond=list(ISPresent=0, ISUngrammatical=0,ISPresentUngrammatical=0),
            col=1, xpd=TRUE, eegAxis = TRUE, ylim = c(2, -2), main="Past: P3")
```

```
## Summary:
##  * Time : numeric predictor; with 30 values ranging from -195.000000 to 1195.000000. 
##  * ISPresent : numeric predictor; set to the value(s): 0. 
##  * ISUngrammatical : numeric predictor; set to the value(s): 0. 
##  * ISPresentUngrammatical : numeric predictor; set to the value(s): 0. 
##  * Event : factor; set to the value(s): Frank01.Past_gram. (Might be canceled as random effect, check below.) 
##  * NOTE : The following random effects columns are canceled: s(Time,Event)
##
```

```
##Plot past ungrammatical
plot_smooth(Mod2, view="Time", rm.ranef=TRUE, 
            cond=list(ISPresent=0, ISUngrammatical=1,ISPresentUngrammatical=0),
            h0=0,add=TRUE, v0=0,col=2)
```

```
## Summary:
##  * Time : numeric predictor; with 30 values ranging from -195.000000 to 1195.000000. 
##  * ISPresent : numeric predictor; set to the value(s): 0. 
##  * ISUngrammatical : numeric predictor; set to the value(s): 1. 
##  * ISPresentUngrammatical : numeric predictor; set to the value(s): 0. 
##  * Event : factor; set to the value(s): Frank01.Past_gram. (Might be canceled as random effect, check below.) 
##  * NOTE : The following random effects columns are canceled: s(Time,Event)
##
```

```
legend_margin('bottomleft', legend=c("grammatical", "ungrammatical"),
       fill=c(alpha(1), alpha(2)), border= c(alpha(1), alpha(2)),
         lwd=1.5, col=c(1,2), seg.len=1, merge=TRUE,
         bty='n')


## Plot differences (i.e. where the grammatical vs ungrammatical lines differ in this case for past)
plot_diff(Mod2, view="Time", cond=list(ISPresent=0, ISPresentUngrammatical=0), 
          comp=list(ISUngrammatical=c(0,1)), 
          rm.ranef = TRUE,
          main="Difference in Past grammaticality")
```

```
## Summary:
##  * Time : numeric predictor; with 100 values ranging from -195.000000 to 1195.000000. 
##  * ISPresent : numeric predictor; set to the value(s): 0. 
##  * ISPresentUngrammatical : numeric predictor; set to the value(s): 0. 
##  * Event : factor; set to the value(s): Frank01.Past_gram. (Might be canceled as random effect, check below.) 
##  * NOTE : The following random effects columns are canceled: s(Time,Event)
##
```

```
## 
## Difference is not significant.
```

```
#Plotting for Present
##Plot present grammatical
plot_smooth(Mod2, view="Time", rm.ranef=TRUE, 
            cond=list(ISPresent=1, ISUngrammatical=0,ISPresentUngrammatical=0),
            col=1, xpd=TRUE, eegAxis = TRUE, ylim = c(2, -2), main="Present: P3")
```

```
## Summary:
##  * Time : numeric predictor; with 30 values ranging from -195.000000 to 1195.000000. 
##  * ISPresent : numeric predictor; set to the value(s): 1. 
##  * ISUngrammatical : numeric predictor; set to the value(s): 0. 
##  * ISPresentUngrammatical : numeric predictor; set to the value(s): 0. 
##  * Event : factor; set to the value(s): Frank01.Past_gram. (Might be canceled as random effect, check below.) 
##  * NOTE : The following random effects columns are canceled: s(Time,Event)
##
```

```
##Plot present ungrammatical
plot_smooth(Mod2, view="Time", rm.ranef=TRUE, 
            cond=list(ISPresent=1, ISUngrammatical=1,ISPresentUngrammatical=1),
            add=TRUE, col=2, xpd=TRUE, main = "Present")
```

```
## Summary:
##  * Time : numeric predictor; with 30 values ranging from -195.000000 to 1195.000000. 
##  * ISPresent : numeric predictor; set to the value(s): 1. 
##  * ISUngrammatical : numeric predictor; set to the value(s): 1. 
##  * ISPresentUngrammatical : numeric predictor; set to the value(s): 1. 
##  * Event : factor; set to the value(s): Frank01.Past_gram. (Might be canceled as random effect, check below.) 
##  * NOTE : The following random effects columns are canceled: s(Time,Event)
##
```

```
## Plot differences (i.e. where the grammatical vs ungrammatical lines differ in this case for present)
plot_diff(Mod2, view="Time", cond=list(ISPresent=1), 
          comp=list(ISUngrammatical=c(0,1), ISPresentUngrammatical=c(0,1)), 
          rm.ranef = TRUE,
          main="Difference in Present grammaticality")
```

```
## Summary:
##  * Time : numeric predictor; with 100 values ranging from -195.000000 to 1195.000000. 
##  * ISPresent : numeric predictor; set to the value(s): 1. 
##  * Event : factor; set to the value(s): Frank01.Past_gram. (Might be canceled as random effect, check below.) 
##  * NOTE : The following random effects columns are canceled: s(Time,Event)
##
```

```
## 
## Difference is not significant.
```

#Subsetting data with electrode: P4

```
subdat <- droplevels(dat[dat$Channel== "P4" & dat$Condition %in% c("Past_gram", "Past_ungram", "Present_gram", "Present_ungram"),]) 
head(subdat)
```

```
##      Subject Condition Grammaticality Channel Time      value Tense
## 3361 Frank01 Past_gram    Grammatical      P4 -195  0.2568308  Past
## 3362 Frank01 Past_gram    Grammatical      P4 -185  1.1269551  Past
## 3363 Frank01 Past_gram    Grammatical      P4 -175  1.2482019  Past
## 3364 Frank01 Past_gram    Grammatical      P4 -165  0.6609846  Past
## 3365 Frank01 Past_gram    Grammatical      P4 -155 -0.1645577  Past
## 3366 Frank01 Past_gram    Grammatical      P4 -145 -0.8608942  Past
##      ISPresent ISUngrammatical ISPresentUngrammatical
## 3361         0               0                      0
## 3362         0               0                      0
## 3363         0               0                      0
## 3364         0               0                      0
## 3365         0               0                      0
## 3366         0               0                      0
```

## Create EVENT (unique combination of Subject and Items)for random effects

```
subdat$Event <- interaction(subdat$Subject, subdat$Condition)

#   Determine starting point for each time series
subdat <- start_event(subdat,column = "Time",event = "Event")

# Make sure variables are factors
subdat$Condition <- as.factor(subdat$Condition) 
subdat$Subject <- as.factor(subdat$Subject)
```

# GAMM ANALYSIS

```
Mod1 <- bam(value ~ s(Time, k=20) 
            + s(Time, by=ISPresent, k=20)
            + s(Time, by=ISUngrammatical, k=20)
            + s(Time, by=ISPresentUngrammatical, k=20)
            + s(Time,Event, bs='fs', m=1, k=20),
            data=subdat, discrete = TRUE, family="scat")
```

```
## Warning in gam.side(sm, X, tol = .Machine$double.eps^0.5): model has
## repeated 1-d smooths of same variable.
```

```
## Calculate rho value to account for autocorrelation in data.
myrho <- start_value_rho(Mod1)

#Fit a Model including rho
Mod2 <- bam(value ~ s(Time, k=20) 
            + s(Time, by=ISPresent, k=20)
            + s(Time, by=ISUngrammatical, k=20)
            + s(Time, by=ISPresentUngrammatical, k=20)
            + s(Time,Event, bs='fs', m=1, k=20),
            data=subdat, discrete = TRUE, family="scat", 
            AR.start=subdat$start.event, rho=myrho)
```

```
## Warning in gam.side(sm, X, tol = .Machine$double.eps^0.5): model has
## repeated 1-d smooths of same variable.
```

```
#Plot model residuals
qqnorm(resid(Mod2))
qqline(resid(Mod2))
```

#PLOTTING OF GAM model

```
par(mfcol =c(2,2))

#Plotting Past
##Plot past grammatical
plot_smooth(Mod2, view="Time", rm.ranef=TRUE, 
            cond=list(ISPresent=0, ISUngrammatical=0,ISPresentUngrammatical=0),
            col=1, xpd=TRUE, eegAxis = TRUE, ylim = c(2, -2), main="Past: P4")
```

```
## Summary:
##  * Time : numeric predictor; with 30 values ranging from -195.000000 to 1195.000000. 
##  * ISPresent : numeric predictor; set to the value(s): 0. 
##  * ISUngrammatical : numeric predictor; set to the value(s): 0. 
##  * ISPresentUngrammatical : numeric predictor; set to the value(s): 0. 
##  * Event : factor; set to the value(s): Frank01.Past_gram. (Might be canceled as random effect, check below.) 
##  * NOTE : The following random effects columns are canceled: s(Time,Event)
##
```

```
##Plot past ungrammatical
plot_smooth(Mod2, view="Time", rm.ranef=TRUE, 
            cond=list(ISPresent=0, ISUngrammatical=1,ISPresentUngrammatical=0),
            h0=0,add=TRUE, v0=0,col=2)
```

```
## Summary:
##  * Time : numeric predictor; with 30 values ranging from -195.000000 to 1195.000000. 
##  * ISPresent : numeric predictor; set to the value(s): 0. 
##  * ISUngrammatical : numeric predictor; set to the value(s): 1. 
##  * ISPresentUngrammatical : numeric predictor; set to the value(s): 0. 
##  * Event : factor; set to the value(s): Frank01.Past_gram. (Might be canceled as random effect, check below.) 
##  * NOTE : The following random effects columns are canceled: s(Time,Event)
##
```

```
legend_margin('bottomleft', legend=c("grammatical", "ungrammatical"),
       fill=c(alpha(1), alpha(2)), border= c(alpha(1), alpha(2)),
         lwd=1.5, col=c(1,2), seg.len=1, merge=TRUE,
         bty='n')


## Plot differences (i.e. where the grammatical vs ungrammatical lines differ in this case for past)
plot_diff(Mod2, view="Time", cond=list(ISPresent=0, ISPresentUngrammatical=0), 
          comp=list(ISUngrammatical=c(0,1)), 
          rm.ranef = TRUE,
          main="Difference in Past grammaticality")
```

```
## Summary:
##  * Time : numeric predictor; with 100 values ranging from -195.000000 to 1195.000000. 
##  * ISPresent : numeric predictor; set to the value(s): 0. 
##  * ISPresentUngrammatical : numeric predictor; set to the value(s): 0. 
##  * Event : factor; set to the value(s): Frank01.Past_gram. (Might be canceled as random effect, check below.) 
##  * NOTE : The following random effects columns are canceled: s(Time,Event)
##
```

```
## 
## Difference is not significant.
```

```
#Plotting for Present
##Plot present grammatical
plot_smooth(Mod2, view="Time", rm.ranef=TRUE, 
            cond=list(ISPresent=1, ISUngrammatical=0,ISPresentUngrammatical=0),
            col=1, xpd=TRUE, eegAxis = TRUE, ylim = c(2, -2), main="Present: P4")
```

```
## Summary:
##  * Time : numeric predictor; with 30 values ranging from -195.000000 to 1195.000000. 
##  * ISPresent : numeric predictor; set to the value(s): 1. 
##  * ISUngrammatical : numeric predictor; set to the value(s): 0. 
##  * ISPresentUngrammatical : numeric predictor; set to the value(s): 0. 
##  * Event : factor; set to the value(s): Frank01.Past_gram. (Might be canceled as random effect, check below.) 
##  * NOTE : The following random effects columns are canceled: s(Time,Event)
##
```

```
##Plot present ungrammatical
plot_smooth(Mod2, view="Time", rm.ranef=TRUE, 
            cond=list(ISPresent=1, ISUngrammatical=1,ISPresentUngrammatical=1),
            add=TRUE, col=2, xpd=TRUE, main = "Present")
```

```
## Summary:
##  * Time : numeric predictor; with 30 values ranging from -195.000000 to 1195.000000. 
##  * ISPresent : numeric predictor; set to the value(s): 1. 
##  * ISUngrammatical : numeric predictor; set to the value(s): 1. 
##  * ISPresentUngrammatical : numeric predictor; set to the value(s): 1. 
##  * Event : factor; set to the value(s): Frank01.Past_gram. (Might be canceled as random effect, check below.) 
##  * NOTE : The following random effects columns are canceled: s(Time,Event)
##
```

```
## Plot differences (i.e. where the grammatical vs ungrammatical lines differ in this case for present)
plot_diff(Mod2, view="Time", cond=list(ISPresent=1), 
          comp=list(ISUngrammatical=c(0,1), ISPresentUngrammatical=c(0,1)), 
          rm.ranef = TRUE,
          main="Difference in Present grammaticality")
```

```
## Summary:
##  * Time : numeric predictor; with 100 values ranging from -195.000000 to 1195.000000. 
##  * ISPresent : numeric predictor; set to the value(s): 1. 
##  * Event : factor; set to the value(s): Frank01.Past_gram. (Might be canceled as random effect, check below.) 
##  * NOTE : The following random effects columns are canceled: s(Time,Event)
##
```

```
## 
## Difference is not significant.
```

#Subsetting data with electrode: P8

```
subdat <- droplevels(dat[dat$Channel== "P8" & dat$Condition %in% c("Past_gram", "Past_ungram", "Present_gram", "Present_ungram"),]) 
head(subdat)
```

```
##      Subject Condition Grammaticality Channel Time      value Tense
## 3501 Frank01 Past_gram    Grammatical      P8 -195  0.4079923  Past
## 3502 Frank01 Past_gram    Grammatical      P8 -185  0.7892500  Past
## 3503 Frank01 Past_gram    Grammatical      P8 -175  1.0440096  Past
## 3504 Frank01 Past_gram    Grammatical      P8 -165  0.9002231  Past
## 3505 Frank01 Past_gram    Grammatical      P8 -155  0.2656474  Past
## 3506 Frank01 Past_gram    Grammatical      P8 -145 -0.1616827  Past
##      ISPresent ISUngrammatical ISPresentUngrammatical
## 3501         0               0                      0
## 3502         0               0                      0
## 3503         0               0                      0
## 3504         0               0                      0
## 3505         0               0                      0
## 3506         0               0                      0
```

## Create EVENT (unique combination of Subject and Items)for random effects

```
subdat$Event <- interaction(subdat$Subject, subdat$Condition)

#   Determine starting point for each time series
subdat <- start_event(subdat,column = "Time",event = "Event")

# Make sure variables are factors
subdat$Condition <- as.factor(subdat$Condition) 
subdat$Subject <- as.factor(subdat$Subject)
```

# GAMM ANALYSIS

```
Mod1 <- bam(value ~ s(Time, k=20) 
            + s(Time, by=ISPresent, k=20)
            + s(Time, by=ISUngrammatical, k=20)
            + s(Time, by=ISPresentUngrammatical, k=20)
            + s(Time,Event, bs='fs', m=1, k=20),
            data=subdat, discrete = TRUE, family="scat")
```

```
## Warning in gam.side(sm, X, tol = .Machine$double.eps^0.5): model has
## repeated 1-d smooths of same variable.
```

```
## Calculate rho value to account for autocorrelation in data.
myrho <- start_value_rho(Mod1)

#Fit a Model including rho
Mod2 <- bam(value ~ s(Time, k=20) 
            + s(Time, by=ISPresent, k=20)
            + s(Time, by=ISUngrammatical, k=20)
            + s(Time, by=ISPresentUngrammatical, k=20)
            + s(Time,Event, bs='fs', m=1, k=20),
            data=subdat, discrete = TRUE, family="scat", 
            AR.start=subdat$start.event, rho=myrho)
```

```
## Warning in gam.side(sm, X, tol = .Machine$double.eps^0.5): model has
## repeated 1-d smooths of same variable.
```

```
#Plot model residuals
qqnorm(resid(Mod2))
qqline(resid(Mod2))
```

#PLOTTING OF GAM model

```
par(mfcol =c(2,2))

#Plotting Past
##Plot past grammatical
plot_smooth(Mod2, view="Time", rm.ranef=TRUE, 
            cond=list(ISPresent=0, ISUngrammatical=0,ISPresentUngrammatical=0),
            col=1, xpd=TRUE, eegAxis = TRUE, ylim = c(2, -2), main="Past: P8")
```

```
## Summary:
##  * Time : numeric predictor; with 30 values ranging from -195.000000 to 1195.000000. 
##  * ISPresent : numeric predictor; set to the value(s): 0. 
##  * ISUngrammatical : numeric predictor; set to the value(s): 0. 
##  * ISPresentUngrammatical : numeric predictor; set to the value(s): 0. 
##  * Event : factor; set to the value(s): Frank01.Past_gram. (Might be canceled as random effect, check below.) 
##  * NOTE : The following random effects columns are canceled: s(Time,Event)
##
```

```
##Plot past ungrammatical
plot_smooth(Mod2, view="Time", rm.ranef=TRUE, 
            cond=list(ISPresent=0, ISUngrammatical=1,ISPresentUngrammatical=0),
            h0=0,add=TRUE, v0=0,col=2)
```

```
## Summary:
##  * Time : numeric predictor; with 30 values ranging from -195.000000 to 1195.000000. 
##  * ISPresent : numeric predictor; set to the value(s): 0. 
##  * ISUngrammatical : numeric predictor; set to the value(s): 1. 
##  * ISPresentUngrammatical : numeric predictor; set to the value(s): 0. 
##  * Event : factor; set to the value(s): Frank01.Past_gram. (Might be canceled as random effect, check below.) 
##  * NOTE : The following random effects columns are canceled: s(Time,Event)
##
```

```
legend_margin('bottomleft', legend=c("grammatical", "ungrammatical"),
       fill=c(alpha(1), alpha(2)), border= c(alpha(1), alpha(2)),
         lwd=1.5, col=c(1,2), seg.len=1, merge=TRUE,
         bty='n')


## Plot differences (i.e. where the grammatical vs ungrammatical lines differ in this case for past)
plot_diff(Mod2, view="Time", cond=list(ISPresent=0, ISPresentUngrammatical=0), 
          comp=list(ISUngrammatical=c(0,1)), 
          rm.ranef = TRUE,
          main="Difference in Past grammaticality")
```

```
## Summary:
##  * Time : numeric predictor; with 100 values ranging from -195.000000 to 1195.000000. 
##  * ISPresent : numeric predictor; set to the value(s): 0. 
##  * ISPresentUngrammatical : numeric predictor; set to the value(s): 0. 
##  * Event : factor; set to the value(s): Frank01.Past_gram. (Might be canceled as random effect, check below.) 
##  * NOTE : The following random effects columns are canceled: s(Time,Event)
##
```

```
## 
## Time window(s) of significant difference(s):
##  521.060606 - 1195.000000
```

```
#Plotting for Present
##Plot present grammatical
plot_smooth(Mod2, view="Time", rm.ranef=TRUE, 
            cond=list(ISPresent=1, ISUngrammatical=0,ISPresentUngrammatical=0),
            col=1, xpd=TRUE, eegAxis = TRUE, ylim = c(2, -2), main="Present: P8")
```

```
## Summary:
##  * Time : numeric predictor; with 30 values ranging from -195.000000 to 1195.000000. 
##  * ISPresent : numeric predictor; set to the value(s): 1. 
##  * ISUngrammatical : numeric predictor; set to the value(s): 0. 
##  * ISPresentUngrammatical : numeric predictor; set to the value(s): 0. 
##  * Event : factor; set to the value(s): Frank01.Past_gram. (Might be canceled as random effect, check below.) 
##  * NOTE : The following random effects columns are canceled: s(Time,Event)
##
```

```
##Plot present ungrammatical
plot_smooth(Mod2, view="Time", rm.ranef=TRUE, 
            cond=list(ISPresent=1, ISUngrammatical=1,ISPresentUngrammatical=1),
            add=TRUE, col=2, xpd=TRUE, main = "Present")
```

```
## Summary:
##  * Time : numeric predictor; with 30 values ranging from -195.000000 to 1195.000000. 
##  * ISPresent : numeric predictor; set to the value(s): 1. 
##  * ISUngrammatical : numeric predictor; set to the value(s): 1. 
##  * ISPresentUngrammatical : numeric predictor; set to the value(s): 1. 
##  * Event : factor; set to the value(s): Frank01.Past_gram. (Might be canceled as random effect, check below.) 
##  * NOTE : The following random effects columns are canceled: s(Time,Event)
##
```

```
## Plot differences (i.e. where the grammatical vs ungrammatical lines differ in this case for present)
plot_diff(Mod2, view="Time", cond=list(ISPresent=1), 
          comp=list(ISUngrammatical=c(0,1), ISPresentUngrammatical=c(0,1)), 
          rm.ranef = TRUE,
          main="Difference in Present grammaticality")
```

```
## Summary:
##  * Time : numeric predictor; with 100 values ranging from -195.000000 to 1195.000000. 
##  * ISPresent : numeric predictor; set to the value(s): 1. 
##  * Event : factor; set to the value(s): Frank01.Past_gram. (Might be canceled as random effect, check below.) 
##  * NOTE : The following random effects columns are canceled: s(Time,Event)
##
```

```
## 
## Difference is not significant.
```

#Subsetting data with electrode: POz

```
subdat <- droplevels(dat[dat$Channel== "POz" & dat$Condition %in% c("Past_gram", "Past_ungram", "Present_gram", "Present_ungram"),]) 
head(subdat)
```

```
##      Subject Condition Grammaticality Channel Time         value Tense
## 3641 Frank01 Past_gram    Grammatical     POz -195  0.6643538462  Past
## 3642 Frank01 Past_gram    Grammatical     POz -185  1.2208974359  Past
## 3643 Frank01 Past_gram    Grammatical     POz -175  0.7529038462  Past
## 3644 Frank01 Past_gram    Grammatical     POz -165  0.2761000000  Past
## 3645 Frank01 Past_gram    Grammatical     POz -155  0.0006602564  Past
## 3646 Frank01 Past_gram    Grammatical     POz -145 -0.1567884615  Past
##      ISPresent ISUngrammatical ISPresentUngrammatical
## 3641         0               0                      0
## 3642         0               0                      0
## 3643         0               0                      0
## 3644         0               0                      0
## 3645         0               0                      0
## 3646         0               0                      0
```

## Create EVENT (unique combination of Subject and Items)for random effects

```
subdat$Event <- interaction(subdat$Subject, subdat$Condition)

#   Determine starting point for each time series
subdat <- start_event(subdat,column = "Time",event = "Event")

# Make sure variables are factors
subdat$Condition <- as.factor(subdat$Condition) 
subdat$Subject <- as.factor(subdat$Subject)
```

# GAMM ANALYSIS

```
Mod1 <- bam(value ~ s(Time, k=20) 
            + s(Time, by=ISPresent, k=20)
            + s(Time, by=ISUngrammatical, k=20)
            + s(Time, by=ISPresentUngrammatical, k=20)
            + s(Time,Event, bs='fs', m=1, k=20),
            data=subdat, discrete = TRUE, family="scat")
```

```
## Warning in gam.side(sm, X, tol = .Machine$double.eps^0.5): model has
## repeated 1-d smooths of same variable.
```

```
## Calculate rho value to account for autocorrelation in data.
myrho <- start_value_rho(Mod1)

#Fit a Model including rho
Mod2 <- bam(value ~ s(Time, k=20) 
            + s(Time, by=ISPresent, k=20)
            + s(Time, by=ISUngrammatical, k=20)
            + s(Time, by=ISPresentUngrammatical, k=20)
            + s(Time,Event, bs='fs', m=1, k=20),
            data=subdat, discrete = TRUE, family="scat", 
            AR.start=subdat$start.event, rho=myrho)
```

```
## Warning in gam.side(sm, X, tol = .Machine$double.eps^0.5): model has
## repeated 1-d smooths of same variable.
```

```
#Plot model residuals
qqnorm(resid(Mod2))
qqline(resid(Mod2))
```

#PLOTTING OF GAM model

```
par(mfcol =c(2,2))

#Plotting Past
##Plot past grammatical
plot_smooth(Mod2, view="Time", rm.ranef=TRUE, 
            cond=list(ISPresent=0, ISUngrammatical=0,ISPresentUngrammatical=0),
            col=1, xpd=TRUE, eegAxis = TRUE, ylim = c(2, -2), main="Past: POz")
```

```
## Summary:
##  * Time : numeric predictor; with 30 values ranging from -195.000000 to 1195.000000. 
##  * ISPresent : numeric predictor; set to the value(s): 0. 
##  * ISUngrammatical : numeric predictor; set to the value(s): 0. 
##  * ISPresentUngrammatical : numeric predictor; set to the value(s): 0. 
##  * Event : factor; set to the value(s): Frank01.Past_gram. (Might be canceled as random effect, check below.) 
##  * NOTE : The following random effects columns are canceled: s(Time,Event)
##
```

```
##Plot past ungrammatical
plot_smooth(Mod2, view="Time", rm.ranef=TRUE, 
            cond=list(ISPresent=0, ISUngrammatical=1,ISPresentUngrammatical=0),
            h0=0,add=TRUE, v0=0,col=2)
```

```
## Summary:
##  * Time : numeric predictor; with 30 values ranging from -195.000000 to 1195.000000. 
##  * ISPresent : numeric predictor; set to the value(s): 0. 
##  * ISUngrammatical : numeric predictor; set to the value(s): 1. 
##  * ISPresentUngrammatical : numeric predictor; set to the value(s): 0. 
##  * Event : factor; set to the value(s): Frank01.Past_gram. (Might be canceled as random effect, check below.) 
##  * NOTE : The following random effects columns are canceled: s(Time,Event)
##
```

```
legend_margin('bottomleft', legend=c("grammatical", "ungrammatical"),
       fill=c(alpha(1), alpha(2)), border= c(alpha(1), alpha(2)),
         lwd=1.5, col=c(1,2), seg.len=1, merge=TRUE,
         bty='n')


## Plot differences (i.e. where the grammatical vs ungrammatical lines differ in this case for past)
plot_diff(Mod2, view="Time", cond=list(ISPresent=0, ISPresentUngrammatical=0), 
          comp=list(ISUngrammatical=c(0,1)), 
          rm.ranef = TRUE,
          main="Difference in Past grammaticality")
```

```
## Summary:
##  * Time : numeric predictor; with 100 values ranging from -195.000000 to 1195.000000. 
##  * ISPresent : numeric predictor; set to the value(s): 0. 
##  * ISPresentUngrammatical : numeric predictor; set to the value(s): 0. 
##  * Event : factor; set to the value(s): Frank01.Past_gram. (Might be canceled as random effect, check below.) 
##  * NOTE : The following random effects columns are canceled: s(Time,Event)
##
```

```
## 
## Difference is not significant.
```

```
#Plotting for Present
##Plot present grammatical
plot_smooth(Mod2, view="Time", rm.ranef=TRUE, 
            cond=list(ISPresent=1, ISUngrammatical=0,ISPresentUngrammatical=0),
            col=1, xpd=TRUE, eegAxis = TRUE, ylim = c(2, -2), main="Present: POz")
```

```
## Summary:
##  * Time : numeric predictor; with 30 values ranging from -195.000000 to 1195.000000. 
##  * ISPresent : numeric predictor; set to the value(s): 1. 
##  * ISUngrammatical : numeric predictor; set to the value(s): 0. 
##  * ISPresentUngrammatical : numeric predictor; set to the value(s): 0. 
##  * Event : factor; set to the value(s): Frank01.Past_gram. (Might be canceled as random effect, check below.) 
##  * NOTE : The following random effects columns are canceled: s(Time,Event)
##
```

```
##Plot present ungrammatical
plot_smooth(Mod2, view="Time", rm.ranef=TRUE, 
            cond=list(ISPresent=1, ISUngrammatical=1,ISPresentUngrammatical=1),
            add=TRUE, col=2, xpd=TRUE, main = "Present")
```

```
## Summary:
##  * Time : numeric predictor; with 30 values ranging from -195.000000 to 1195.000000. 
##  * ISPresent : numeric predictor; set to the value(s): 1. 
##  * ISUngrammatical : numeric predictor; set to the value(s): 1. 
##  * ISPresentUngrammatical : numeric predictor; set to the value(s): 1. 
##  * Event : factor; set to the value(s): Frank01.Past_gram. (Might be canceled as random effect, check below.) 
##  * NOTE : The following random effects columns are canceled: s(Time,Event)
##
```

```
## Plot differences (i.e. where the grammatical vs ungrammatical lines differ in this case for present)
plot_diff(Mod2, view="Time", cond=list(ISPresent=1), 
          comp=list(ISUngrammatical=c(0,1), ISPresentUngrammatical=c(0,1)), 
          rm.ranef = TRUE,
          main="Difference in Present grammaticality")
```

```
## Summary:
##  * Time : numeric predictor; with 100 values ranging from -195.000000 to 1195.000000. 
##  * ISPresent : numeric predictor; set to the value(s): 1. 
##  * Event : factor; set to the value(s): Frank01.Past_gram. (Might be canceled as random effect, check below.) 
##  * NOTE : The following random effects columns are canceled: s(Time,Event)
##
```

```
## 
## Difference is not significant.
```

#Subsetting data with electrode: O1

```
subdat <- droplevels(dat[dat$Channel== "O1" & dat$Condition %in% c("Past_gram", "Past_ungram", "Present_gram", "Present_ungram"),]) 
head(subdat)
```

```
##      Subject Condition Grammaticality Channel Time       value Tense
## 3781 Frank01 Past_gram    Grammatical      O1 -195  0.44163077  Past
## 3782 Frank01 Past_gram    Grammatical      O1 -185  0.33092949  Past
## 3783 Frank01 Past_gram    Grammatical      O1 -175  0.05048077  Past
## 3784 Frank01 Past_gram    Grammatical      O1 -165  0.77892308  Past
## 3785 Frank01 Past_gram    Grammatical      O1 -155  0.49139744  Past
## 3786 Frank01 Past_gram    Grammatical      O1 -145 -0.56695192  Past
##      ISPresent ISUngrammatical ISPresentUngrammatical
## 3781         0               0                      0
## 3782         0               0                      0
## 3783         0               0                      0
## 3784         0               0                      0
## 3785         0               0                      0
## 3786         0               0                      0
```

## Create EVENT (unique combination of Subject and Items)for random effects

```
subdat$Event <- interaction(subdat$Subject, subdat$Condition)

#   Determine starting point for each time series
subdat <- start_event(subdat,column = "Time",event = "Event")

# Make sure variables are factors
subdat$Condition <- as.factor(subdat$Condition) 
subdat$Subject <- as.factor(subdat$Subject)
```

# GAMM ANALYSIS

```
Mod1 <- bam(value ~ s(Time, k=20) 
            + s(Time, by=ISPresent, k=20)
            + s(Time, by=ISUngrammatical, k=20)
            + s(Time, by=ISPresentUngrammatical, k=20)
            + s(Time,Event, bs='fs', m=1, k=20),
            data=subdat, discrete = TRUE, family="scat")
```

```
## Warning in gam.side(sm, X, tol = .Machine$double.eps^0.5): model has
## repeated 1-d smooths of same variable.
```

```
## Calculate rho value to account for autocorrelation in data.
myrho <- start_value_rho(Mod1)

#Fit a Model including rho
Mod2 <- bam(value ~ s(Time, k=20) 
            + s(Time, by=ISPresent, k=20)
            + s(Time, by=ISUngrammatical, k=20)
            + s(Time, by=ISPresentUngrammatical, k=20)
            + s(Time,Event, bs='fs', m=1, k=20),
            data=subdat, discrete = TRUE, family="scat", 
            AR.start=subdat$start.event, rho=myrho)
```

```
## Warning in gam.side(sm, X, tol = .Machine$double.eps^0.5): model has
## repeated 1-d smooths of same variable.
```

```
#Plot model residuals
qqnorm(resid(Mod2))
qqline(resid(Mod2))
```

#PLOTTING OF GAM model

```
par(mfcol =c(2,2))

#Plotting Past
##Plot past grammatical
plot_smooth(Mod2, view="Time", rm.ranef=TRUE, 
            cond=list(ISPresent=0, ISUngrammatical=0,ISPresentUngrammatical=0),
            col=1, xpd=TRUE, eegAxis = TRUE, ylim = c(2, -2), main="Past: O1")
```

```
## Summary:
##  * Time : numeric predictor; with 30 values ranging from -195.000000 to 1195.000000. 
##  * ISPresent : numeric predictor; set to the value(s): 0. 
##  * ISUngrammatical : numeric predictor; set to the value(s): 0. 
##  * ISPresentUngrammatical : numeric predictor; set to the value(s): 0. 
##  * Event : factor; set to the value(s): Frank01.Past_gram. (Might be canceled as random effect, check below.) 
##  * NOTE : The following random effects columns are canceled: s(Time,Event)
##
```

```
##Plot past ungrammatical
plot_smooth(Mod2, view="Time", rm.ranef=TRUE, 
            cond=list(ISPresent=0, ISUngrammatical=1,ISPresentUngrammatical=0),
            h0=0,add=TRUE, v0=0,col=2)
```

```
## Summary:
##  * Time : numeric predictor; with 30 values ranging from -195.000000 to 1195.000000. 
##  * ISPresent : numeric predictor; set to the value(s): 0. 
##  * ISUngrammatical : numeric predictor; set to the value(s): 1. 
##  * ISPresentUngrammatical : numeric predictor; set to the value(s): 0. 
##  * Event : factor; set to the value(s): Frank01.Past_gram. (Might be canceled as random effect, check below.) 
##  * NOTE : The following random effects columns are canceled: s(Time,Event)
##
```

```
legend_margin('bottomleft', legend=c("grammatical", "ungrammatical"),
       fill=c(alpha(1), alpha(2)), border= c(alpha(1), alpha(2)),
         lwd=1.5, col=c(1,2), seg.len=1, merge=TRUE,
         bty='n')


## Plot differences (i.e. where the grammatical vs ungrammatical lines differ in this case for past)
plot_diff(Mod2, view="Time", cond=list(ISPresent=0, ISPresentUngrammatical=0), 
          comp=list(ISUngrammatical=c(0,1)), 
          rm.ranef = TRUE,
          main="Difference in Past grammaticality")
```

```
## Summary:
##  * Time : numeric predictor; with 100 values ranging from -195.000000 to 1195.000000. 
##  * ISPresent : numeric predictor; set to the value(s): 0. 
##  * ISPresentUngrammatical : numeric predictor; set to the value(s): 0. 
##  * Event : factor; set to the value(s): Frank01.Past_gram. (Might be canceled as random effect, check below.) 
##  * NOTE : The following random effects columns are canceled: s(Time,Event)
##
```

```
## 
## Difference is not significant.
```

```
#Plotting for Present
##Plot present grammatical
plot_smooth(Mod2, view="Time", rm.ranef=TRUE, 
            cond=list(ISPresent=1, ISUngrammatical=0,ISPresentUngrammatical=0),
            col=1, xpd=TRUE, eegAxis = TRUE, ylim = c(2, -2), main="Present: O1")
```

```
## Summary:
##  * Time : numeric predictor; with 30 values ranging from -195.000000 to 1195.000000. 
##  * ISPresent : numeric predictor; set to the value(s): 1. 
##  * ISUngrammatical : numeric predictor; set to the value(s): 0. 
##  * ISPresentUngrammatical : numeric predictor; set to the value(s): 0. 
##  * Event : factor; set to the value(s): Frank01.Past_gram. (Might be canceled as random effect, check below.) 
##  * NOTE : The following random effects columns are canceled: s(Time,Event)
##
```

```
##Plot present ungrammatical
plot_smooth(Mod2, view="Time", rm.ranef=TRUE, 
            cond=list(ISPresent=1, ISUngrammatical=1,ISPresentUngrammatical=1),
            add=TRUE, col=2, xpd=TRUE, main = "Present")
```

```
## Summary:
##  * Time : numeric predictor; with 30 values ranging from -195.000000 to 1195.000000. 
##  * ISPresent : numeric predictor; set to the value(s): 1. 
##  * ISUngrammatical : numeric predictor; set to the value(s): 1. 
##  * ISPresentUngrammatical : numeric predictor; set to the value(s): 1. 
##  * Event : factor; set to the value(s): Frank01.Past_gram. (Might be canceled as random effect, check below.) 
##  * NOTE : The following random effects columns are canceled: s(Time,Event)
##
```

```
## Plot differences (i.e. where the grammatical vs ungrammatical lines differ in this case for present)
plot_diff(Mod2, view="Time", cond=list(ISPresent=1), 
          comp=list(ISUngrammatical=c(0,1), ISPresentUngrammatical=c(0,1)), 
          rm.ranef = TRUE,
          main="Difference in Present grammaticality")
```

```
## Summary:
##  * Time : numeric predictor; with 100 values ranging from -195.000000 to 1195.000000. 
##  * ISPresent : numeric predictor; set to the value(s): 1. 
##  * Event : factor; set to the value(s): Frank01.Past_gram. (Might be canceled as random effect, check below.) 
##  * NOTE : The following random effects columns are canceled: s(Time,Event)
##
```

```
## 
## Difference is not significant.
```

#Subsetting data with electrode: O2

```
subdat <- droplevels(dat[dat$Channel== "O2" & dat$Condition %in% c("Past_gram", "Past_ungram", "Present_gram", "Present_ungram"),]) 
head(subdat)
```

```
##      Subject Condition Grammaticality Channel Time      value Tense
## 3921 Frank01 Past_gram    Grammatical      O2 -195 0.79652308  Past
## 3922 Frank01 Past_gram    Grammatical      O2 -185 1.18416667  Past
## 3923 Frank01 Past_gram    Grammatical      O2 -175 1.24950000  Past
## 3924 Frank01 Past_gram    Grammatical      O2 -165 1.27829231  Past
## 3925 Frank01 Past_gram    Grammatical      O2 -155 0.53687821  Past
## 3926 Frank01 Past_gram    Grammatical      O2 -145 0.06741346  Past
##      ISPresent ISUngrammatical ISPresentUngrammatical
## 3921         0               0                      0
## 3922         0               0                      0
## 3923         0               0                      0
## 3924         0               0                      0
## 3925         0               0                      0
## 3926         0               0                      0
```

## Create EVENT (unique combination of Subject and Items)for random effects

```
subdat$Event <- interaction(subdat$Subject, subdat$Condition)

#   Determine starting point for each time series
subdat <- start_event(subdat,column = "Time",event = "Event")

# Make sure variables are factors
subdat$Condition <- as.factor(subdat$Condition) 
subdat$Subject <- as.factor(subdat$Subject)
```

# GAMM ANALYSIS

```
Mod1 <- bam(value ~ s(Time, k=20) 
            + s(Time, by=ISPresent, k=20)
            + s(Time, by=ISUngrammatical, k=20)
            + s(Time, by=ISPresentUngrammatical, k=20)
            + s(Time,Event, bs='fs', m=1, k=20),
            data=subdat, discrete = TRUE, family="scat")
```

```
## Warning in gam.side(sm, X, tol = .Machine$double.eps^0.5): model has
## repeated 1-d smooths of same variable.
```

```
## Calculate rho value to account for autocorrelation in data.
myrho <- start_value_rho(Mod1)

#Fit a Model including rho
Mod2 <- bam(value ~ s(Time, k=20) 
            + s(Time, by=ISPresent, k=20)
            + s(Time, by=ISUngrammatical, k=20)
            + s(Time, by=ISPresentUngrammatical, k=20)
            + s(Time,Event, bs='fs', m=1, k=20),
            data=subdat, discrete = TRUE, family="scat", 
            AR.start=subdat$start.event, rho=myrho)
```

```
## Warning in gam.side(sm, X, tol = .Machine$double.eps^0.5): model has
## repeated 1-d smooths of same variable.
```

```
#Plot model residuals
qqnorm(resid(Mod2))
qqline(resid(Mod2))
```

#PLOTTING OF GAM model

```
par(mfcol =c(2,2))

#Plotting Past
##Plot past grammatical
plot_smooth(Mod2, view="Time", rm.ranef=TRUE, 
            cond=list(ISPresent=0, ISUngrammatical=0,ISPresentUngrammatical=0),
            col=1, xpd=TRUE, eegAxis = TRUE, ylim = c(2, -2), main="Past: O2")
```

```
## Summary:
##  * Time : numeric predictor; with 30 values ranging from -195.000000 to 1195.000000. 
##  * ISPresent : numeric predictor; set to the value(s): 0. 
##  * ISUngrammatical : numeric predictor; set to the value(s): 0. 
##  * ISPresentUngrammatical : numeric predictor; set to the value(s): 0. 
##  * Event : factor; set to the value(s): Frank01.Past_gram. (Might be canceled as random effect, check below.) 
##  * NOTE : The following random effects columns are canceled: s(Time,Event)
##
```

```
##Plot past ungrammatical
plot_smooth(Mod2, view="Time", rm.ranef=TRUE, 
            cond=list(ISPresent=0, ISUngrammatical=1,ISPresentUngrammatical=0),
            h0=0,add=TRUE, v0=0,col=2)
```

```
## Summary:
##  * Time : numeric predictor; with 30 values ranging from -195.000000 to 1195.000000. 
##  * ISPresent : numeric predictor; set to the value(s): 0. 
##  * ISUngrammatical : numeric predictor; set to the value(s): 1. 
##  * ISPresentUngrammatical : numeric predictor; set to the value(s): 0. 
##  * Event : factor; set to the value(s): Frank01.Past_gram. (Might be canceled as random effect, check below.) 
##  * NOTE : The following random effects columns are canceled: s(Time,Event)
##
```

```
legend_margin('bottomleft', legend=c("grammatical", "ungrammatical"),
       fill=c(alpha(1), alpha(2)), border= c(alpha(1), alpha(2)),
         lwd=1.5, col=c(1,2), seg.len=1, merge=TRUE,
         bty='n')


## Plot differences (i.e. where the grammatical vs ungrammatical lines differ in this case for past)
plot_diff(Mod2, view="Time", cond=list(ISPresent=0, ISPresentUngrammatical=0), 
          comp=list(ISUngrammatical=c(0,1)), 
          rm.ranef = TRUE,
          main="Difference in Past grammaticality")
```

```
## Summary:
##  * Time : numeric predictor; with 100 values ranging from -195.000000 to 1195.000000. 
##  * ISPresent : numeric predictor; set to the value(s): 0. 
##  * ISPresentUngrammatical : numeric predictor; set to the value(s): 0. 
##  * Event : factor; set to the value(s): Frank01.Past_gram. (Might be canceled as random effect, check below.) 
##  * NOTE : The following random effects columns are canceled: s(Time,Event)
##
```

```
## 
## Difference is not significant.
```

```
#Plotting for Present
##Plot present grammatical
plot_smooth(Mod2, view="Time", rm.ranef=TRUE, 
            cond=list(ISPresent=1, ISUngrammatical=0,ISPresentUngrammatical=0),
            col=1, xpd=TRUE, eegAxis = TRUE, ylim = c(2, -2), main="Present: O2")
```

```
## Summary:
##  * Time : numeric predictor; with 30 values ranging from -195.000000 to 1195.000000. 
##  * ISPresent : numeric predictor; set to the value(s): 1. 
##  * ISUngrammatical : numeric predictor; set to the value(s): 0. 
##  * ISPresentUngrammatical : numeric predictor; set to the value(s): 0. 
##  * Event : factor; set to the value(s): Frank01.Past_gram. (Might be canceled as random effect, check below.) 
##  * NOTE : The following random effects columns are canceled: s(Time,Event)
##
```

```
##Plot present ungrammatical
plot_smooth(Mod2, view="Time", rm.ranef=TRUE, 
            cond=list(ISPresent=1, ISUngrammatical=1,ISPresentUngrammatical=1),
            add=TRUE, col=2, xpd=TRUE, main = "Present")
```

```
## Summary:
##  * Time : numeric predictor; with 30 values ranging from -195.000000 to 1195.000000. 
##  * ISPresent : numeric predictor; set to the value(s): 1. 
##  * ISUngrammatical : numeric predictor; set to the value(s): 1. 
##  * ISPresentUngrammatical : numeric predictor; set to the value(s): 1. 
##  * Event : factor; set to the value(s): Frank01.Past_gram. (Might be canceled as random effect, check below.) 
##  * NOTE : The following random effects columns are canceled: s(Time,Event)
##
```

```
## Plot differences (i.e. where the grammatical vs ungrammatical lines differ in this case for present)
plot_diff(Mod2, view="Time", cond=list(ISPresent=1), 
          comp=list(ISUngrammatical=c(0,1), ISPresentUngrammatical=c(0,1)), 
          rm.ranef = TRUE,
          main="Difference in Present grammaticality")
```

```
## Summary:
##  * Time : numeric predictor; with 100 values ranging from -195.000000 to 1195.000000. 
##  * ISPresent : numeric predictor; set to the value(s): 1. 
##  * Event : factor; set to the value(s): Frank01.Past_gram. (Might be canceled as random effect, check below.) 
##  * NOTE : The following random effects columns are canceled: s(Time,Event)
##
```

```
## 
## Difference is not significant.
```
