# Supplementary material for: Online Processing of Temporal Agreement in a Grammatical Tone Language: An ERP Study
Source: Front Psychol. 2021 May 21;12:638716. doi: 10.3389/fpsyg.2021.638716 (PMC8176019; doi:10.3389/fpsyg.2021.638716)
Supplement: Supplementary file 1 [file Data_Sheet_1.ZIP › Supplementary files/Supplementary Material 3.pdf]

Supplementary Material 3

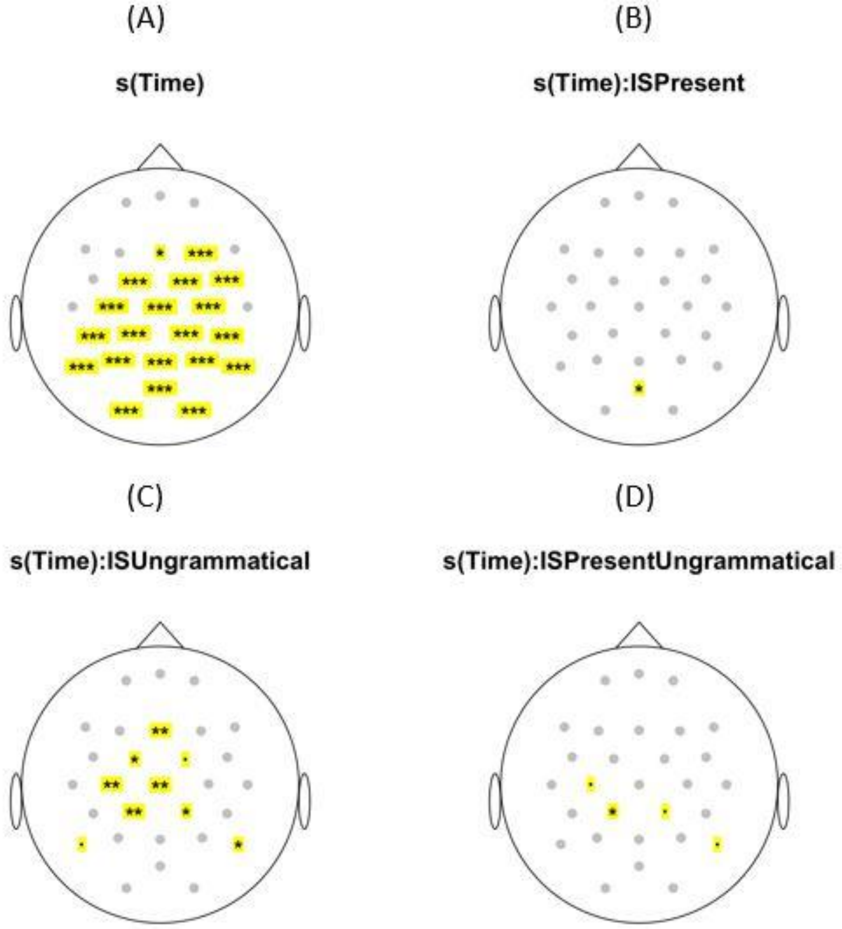

Figure 2. Summary Statistics: Overview of summary statistics of nonlinear fixed-effects. Each GAMM modelled the interaction between Time, Verb form, and Grammaticality with four nonlinear terms: s(Time) + s(Time, by=ISPresent) + s(Time, by=ISUngrammatical) + s(Time, by=ISPresentUngrammatical). Each panel below shows the summary statistics for one term in each GAMM.

(A): Summary statistics for s(Time), representing the time course of the reference level Past Grammatical. Significance here means that the time course is somewhere in the time window significantly different from an amplitude of 0.

(B): Summary statistics for s(Time, by=ISPresent), representing the difference between Past Grammatical (reference level) and Present Grammatical. Significance here means that the difference between these conditions is significant somewhere in the time window.
(C): Summary statistics for s(Time, by=ISUngrammatical), representing the difference between Past Grammatical (reference level) and Past Ungrammatical. Significance here means that the difference between these conditions is significant somewhere in the time window.
(D): Summary statistics for s(Time, by=ISPresentUngrammatical), representing the additive difference between the difference between Present Grammatical and Present Ungrammatical in comparison to the difference between Past Grammatical and Past Ungrammatical. Significance here means that the difference between these differences is significant somewhere in the time window.
Signif. codes: '***' = $p<0.001$; '**' = $p<0.01$; '*' = $p<0.05$; '.' = $p<0.1$.
